# The use of automated insulin delivery around physical activity and exercise in type 1 diabetes: a position statement of the European Association for the Study of Diabetes (EASD) and the International Society for Pediatric and Adolescent Diabetes (ISPAD)

**DOI:** 10.1007/s00125-024-06308-z

**Published:** 2024-12-10

**Authors:** Othmar Moser, Dessi P. Zaharieva, Peter Adolfsson, Tadej Battelino, Richard M. Bracken, Bruce A. Buckingham, Thomas Danne, Elizabeth A. Davis, Klemen Dovč, Gregory P. Forlenza, Pieter Gillard, Sabine E. Hofer, Roman Hovorka, Peter G. Jacobs, Julia K. Mader, Chantal Mathieu, Kirsten Nørgaard, Nick S. Oliver, David N. O’Neal, John Pemberton, Rémi Rabasa-Lhoret, Jennifer L. Sherr, Harald Sourij, Martin Tauschmann, Jane E. Yardley, Michael C. Riddell

**Affiliations:** 1 Department of Exercise Physiology and Metabolism (Sportsmedicine), University of Bayreuth, Bayreuth, Germany; 2 Division of Endocrinology and Diabetology, Medical University of Graz, Graz, Austria; 3 Interdisciplinary Metabolic Medicine Trials Unit, Medical University of Graz, Graz, Austria; 4 Division of Pediatric Endocrinology, Department of Pediatrics, Stanford University, Stanford, CA USA; 5 Department of Pediatrics, Kungsbacka Hospital, Kungsbacka, Sweden; 6 Institute of Clinical Sciences, Sahlgrenska Academy, University of Gothenburg, Gothenburg, Sweden; 7 Department of Endocrinology, Diabetes and Metabolism, University Medical Center, University Children’s Hospital, Ljubljana, Slovenia; 8 Faculty of Medicine, University of Ljubljana, Ljubljana, Slovenia; 9 Applied Sport, Technology, Exercise and Medicine Research Centre, Swansea University, Swansea, UK; 10 Breakthrough T1D (formerly JDRF), New York, NY USA; 11 Centre for Paediatric Endocrinology, Diabetology and Clinical Research, Auf Der Bult Children’s Hospital, Hannover, Germany; 12 Department of Endocrinology and Diabetes, Perth Children’s Hospital, Nedlands, WA Australia; 13 Telethon Kids Institute, University of Western Australia, Perth, WA Australia; 14 Centre for Child Health Research, University of Western Australia, Perth, WA Australia; 15 Barbara Davis Center for Childhood Diabetes, University of Colorado Denver, Denver, CO USA; 16 Department of Endocrinology, University Hospitals Leuven, KU Leuven, Leuven, Belgium; 17 Department of Pediatrics, Medical University of Innsbruck, Innsbruck, Austria; 18 Department of Paediatrics, University of Cambridge, Cambridge, UK; 19 Wellcome Trust-MRC Institute of Metabolic Science, University of Cambridge, Cambridge, UK; 20 Artificial Intelligence for Medical Systems, Department of Biomedical Engineering, Oregon Health & Science University, Portland, OR USA; 21 Department of Clinical Research, Steno Diabetes Center Copenhagen, Herlev, Denmark; 22 Department of Clinical Medicine, Faculty of Health and Medical Sciences, University of Copenhagen, Copenhagen, Denmark; 23 Division of Diabetes, Endocrinology and Metabolism, Department of Medicine, Faculty of Medicine, Imperial College London, London, UK; 24 Department of Medicine, University of Melbourne, Melbourne, VIC Australia; 25 Department of Endocrinology and Diabetes, St Vincent’s Hospital Melbourne, Melbourne, VIC Australia; 26 Australian Centre for Accelerating Diabetes Innovations, Melbourne, VIC Australia; 27 Department of Endocrinology and Diabetes, Birmingham Children’s Hospital, Birmingham Women’s and Children’s NHS Foundation Trust, Birmingham, UK; 28 Montreal Clinical Research Institute (IRCM), Montreal, QC Canada; 29 Department of Nutrition, Faculty of Medicine, Université de Montréal, Montréal, QC Canada; 30 Centre Hospitalier de l‘Université de Montréal Endocrinology Division and CHUM Research Center, Montréal, QC Canada; 31 Division of Pediatric Endocrinology, Department of Pediatrics, Yale University School of Medicine, New Haven, CT USA; 32 Department of Pediatrics and Adolescent Medicine, Medical University of Vienna, Vienna, Austria; 33 School of Kinesiology and Physical Activity Sciences, Faculty of Medicine, University of Montreal, Montreal, QC Canada; 34 School of Kinesiology and Health Science, York University, Toronto, ON Canada

**Keywords:** Automated insulin delivery, CGM, Exercise, Glucose, Insulin pump, Physical activity, Position statement, Type 1 diabetes

## Abstract

Regular physical activity and exercise (PA) are cornerstones of diabetes care for individuals with type 1 diabetes. In recent years, the availability of automated insulin delivery (AID) systems has improved the ability of people with type 1 diabetes to achieve the recommended glucose target ranges. PA provide additional health benefits but can cause glucose fluctuations, which challenges current AID systems. While an increasing number of clinical trials and reviews are being published on different AID systems and PA, it seems prudent at this time to collate this information and develop a position statement on the topic. This joint European Association for the Study of Diabetes (EASD)/International Society for Pediatric and Adolescent Diabetes (ISPAD) position statement reviews current evidence on AID systems and provides detailed clinical practice points for managing PA in children, adolescents and adults with type 1 diabetes using AID technology. It discusses each commercially available AID system individually and provides guidance on their use in PA. Additionally, it addresses different glucose responses to PA and provides stratified therapy options to maintain glucose levels within the target ranges for these age groups.

**Graphical Abstract:**

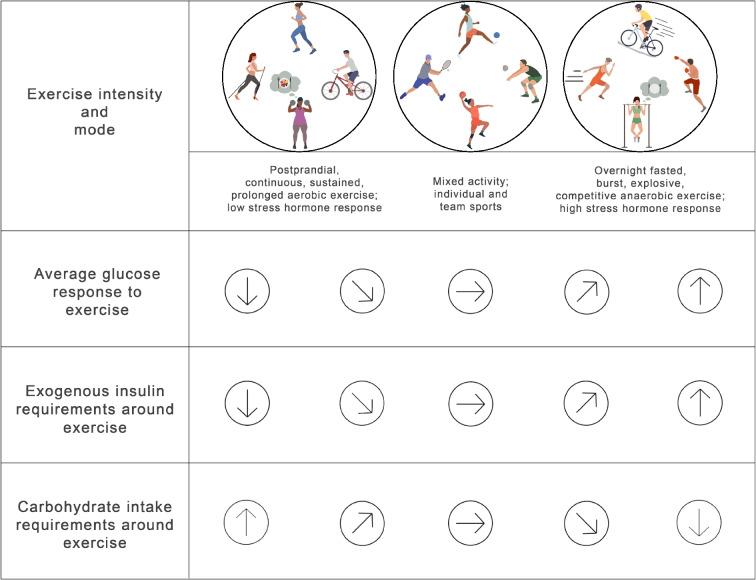

**Supplementary Information:**

The online version contains peer-reviewed but unedited supplementary material including a slideset of the figures for download, which is available to authorised users at 10.1007/s00125-024-06308-z.

## Introduction

**Fig. 1:**
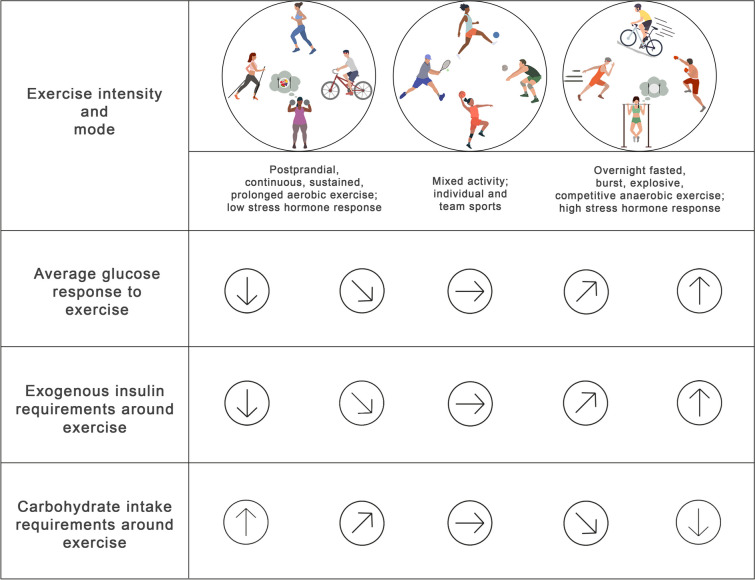
This figure provides a general overview of glucose trends and exogenous insulin and carbohydrate intake requirements in response to PA in people with type 1 diabetes and does not completely reflect the variability that may exist within and between each individual and in different PA types. The average glucose responses to exercise (top row) are highly variable based on several factors including insulin on board, baseline glucose, glucose rate of change, time of day, fitness level, prandial state, fasted state and menstrual cycle phase [21, 67, 69]. People with type 1 diabetes should understand their individual responses to different types of activity [69] and in different settings (e.g. morning vs afternoon [22], practice vs competition [70]). Strategies can then be individualised based on their average glucose responses. No one PA can be associated with one glucose trend; however, activities shown in the upper left panel tend to result in the glucose trends in the first two columns; activities shown in the upper right panel tend to result in the glucose trends in the last two columns; and activities shown in the middle panel can result in the glucose trends in the middle three columns. When considering an increase in insulin dose around PA, this should be discussed with the healthcare professional and care team, as only a few studies have investigated higher insulin doses for exercise. This figure is available as part of a downloadable slideset

Regular moderate-to-vigorous physical activity and exercise (PA) can be beneficial for managing type 1 diabetes [1, 2]. While previous position statements have provided guidance on glucose management during exercise based on glycaemic trends and continuous glucose monitoring (CGM) (Fig. 1 [1–4]), recommendations on using current commercially available automated insulin delivery (AID) systems for PA are limited [5–8]. The general glucose responses to PA and considerations for insulin dose changes and carbohydrate (CHO) intake, as shown in Fig. 1, lay the foundation for the general principles of AID use described below.

In general, people with type 1 diabetes with lower incomes often face numerous challenges that limit their opportunities to adopt technology, including access to insulin pump therapy and CGM systems, not to mention AID systems [9]. Although the prevalence of type 1 diabetes is increasing globally, it is estimated that only 800,000 individuals are currently using AID systems [10], with significant regional differences in access and insurance support. People with type 1 diabetes using AID technology often face significant challenges around meals [11, 12] and PA, both planned and unplanned [8]. Furthermore, several barriers to PA exist (e.g. fear of hypoglycaemia) that may increase the risk of diabetes distress [13]. At present, AID users are required to manually announce meals and adjust for anticipated PA. Some of the technical limitations include a CGM ‘lag time’ between blood and interstitial glucose concentrations with rapid changes in glucose levels [14–19]. In this joint European Association for the Study of Diabetes (EASD)/International Society for Pediatric and Adolescent Diabetes (ISPAD) position statement, we discuss the existing evidence on commercially available AID systems around PA and provide recommendations for managing a physically active lifestyle in children, adolescents and adults with type 1 diabetes. For additional information on emerging AID technology for use in type 1 diabetes, see electronic supplementary material (ESM 1). This position statement is intended for both healthcare professionals and individuals with type 1 diabetes and aims to provide strategies for effective glucose management around planned and unplanned PA. Self-management of PA is often challenging for individuals with type 1 diabetes, no matter what type of insulin therapy they are using. This document provides a comprehensive overview of PA and current AID systems that will serve as a starting point to better manage PA safely and effectively.

## Methods used for group consensus

The writing group members were selected by OM and MCR (4 October 2023) based on publication record and/or clinical experience in the field of AID and PA and approved by the EASD (7 May 2024; see ESM 2). Following initial discussions with specific members of the writing group (OM, DPZ, SEH, JKM, CM, HS, MCR), a first draft was produced by the co-first authors (OM and DPZ) and circulated to the writing group for further discussions and feedback (5 April 2024). Consensus meetings were held online on 21 and 22 May 2024 and consensus was obtained by means of the Delphi technique. After consensus feedback from co-authors was addressed and consensus was met, an updated version of the position statement was sent to three individuals with type 1 diabetes, three parents of children/adolescents with type 1 diabetes and three experts working in the field of PA and type 1 diabetes (14 June 2024). After consideration of their comments, the final version of the joint EASD/ISPAD position statement was sent to the writing group for approval (30 August 2024). The document was then reviewed by EASD’s Committee on Clinical Affairs (CCA) and endorsed by the Boards of EASD and ISPAD.

## Data sources, searches and study selection

The writing group used previous position statements as guidance for the current position statement [1–4]. A literature search was conducted by two independent researchers (OM and DPZ) in PubMed, EMBASE and The Cochrane Library for publications involving AID systems and PA in children/adolescents and/or adults with type 1 diabetes. Details on the keywords and the search strategies are available in ESM 3.

The strengths of the recommendations in this position statement are categorised as A–D. Additionally, ‘consensus D’ reflects clinical experience of respected authorities (see ESM 1 for further details).

## Consensus recommendations

We recommend five key strategies for PA and diabetes management when using AID technology (see Text box, ‘Consensus recommendations for PA and AID in type 1 diabetes’). The authors generalise that these consensus recommendations will work for a majority but not all types of PA [20] **(consensus D)**.


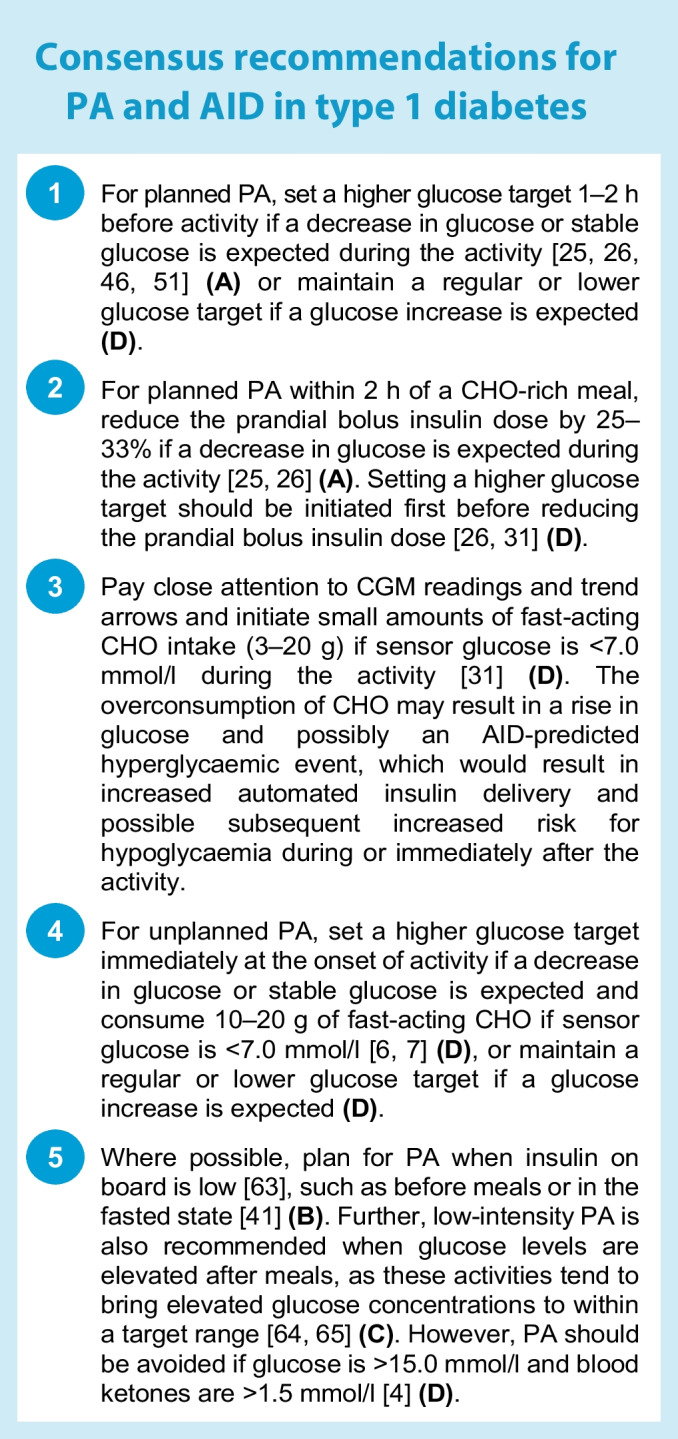


## General principles of AID, physical activity and exercise

As a wide range of exercise types (and activity settings) exist, different recommendations for PA are necessary depending on the type of activity performed and whether that activity is planned or unplanned (Table 1) [6–8]. These general recommendations can serve as starting points that can be incorporated into each specific AID system. Most AID systems have the option to alter (i.e. raise and/or lower) the target glucose value before, during and after PA, which can help maintain glucose levels in the target range. Depending on the AID system being used, the feature that raises the glucose target (or range) is sometimes called exercise mode, activity mode, activity feature, physical activity mode or temporary target; however, for simplicity, we use the term ‘higher glucose target’ for this feature in this position statement.

**Table 1:** General considerations for PA with AID systems

Information	Considerations for PA
CGM trend arrow	• Try to start an activity under stable glucose conditions (e.g. horizontal trend arrow) [63] **(D)** • Immediate postprandial PA increases hypoglycaemia risk [26, 31, 50, 66]; pay close attention to CGM trend arrows and initiate CHO intake if glucose drops <7.0 mmol/l **(C)**
Modification of glucose target (i.e. exercise announcement)	• Aim to set a higher glucose target 1–2 h before PA if a decrease in glucose is expected [25, 26, 46, 51] **(A)** • Setting a higher glucose target at activity onset is less likely to be effective at reducing hypoglycaemia risk, but is still encouraged, particularly when activity is prolonged (e.g. >30 min) **(consensus D)** • Exercise announcement (i.e. higher glucose target) may not be needed in all situations (i.e. if an increase in glucose is expected) [22, 23, 41] **(D)**
Insulin on board (IOB)	• Higher IOB is a predictor of hypoglycaemia risk during PA [63, 67, 68]; however, the calculation and display of IOB depends on the AID system used and various user settings • IOB displayed on an AID system does not accurately reflect peak insulin action, which typically occurs 1–2 h after the prandial bolus dose (see ‘Exercise time of day and prandial status’ below) • In general, aim for low IOB at onset of PA if a decrease in glucose is expected during activity [63, 67, 68] **(C)** • Consider the unadjusted/regular prandial dose and/or insulin delivery settings if an increase in glucose is expected [23, 41] **(D)**
Carbohydrates (CHO)	• Optimise the type of CHO that works best for an individual to maintain glucose levels between 7.0 and 10.0 mmol/l (e.g. fast-acting CHO to treat or prevent hypoglycaemia; low glycaemic index CHO for meal pre exercise) **(consensus D)** • Consume CHO in small amounts (10–20 g) just before PA if sensor glucose is <7.0 mmol/l before unplanned PA, or as needed during PA (e.g. every 30 min) • Determine the threshold at which to consume CHO (e.g. <7.0 mmol/l) [4, 7, 28, 30, 31]. Overconsumption of CHO to treat hypoglycaemia may result in rebound hyperglycaemia followed by AID-induced hypoglycaemia during activity; consider consuming around 12–20 g CHO if hypoglycaemia occurs during activity **(consensus D)** • Always have fast-acting CHO available to prevent or treat hypoglycaemia • Consider carrying emergency supplies (e.g. glucagon) to treat severe hypoglycaemia • Various factors can impact the intra-individual amount of CHO intake needed around PA (e.g. menstrual cycle, hormones, puberty)
Exercise time of day and prandial status	• Reduce the prandial bolus insulin dose (by 25–33%) for meals consumed <2 h prior to PA when a decrease in glucose is expected during the activity [25, 26, 50] **(A)** • Employ the usual prandial bolus insulin dose for meals before PA when an increase in glucose is expected during the activity [22, 23, 41] **(D)** • In general, morning fasted PA is not associated with a large drop in glucose levels and may promote a rise compared with other times of the day (i.e. may be safer for hypoglycaemia risk mitigation) [67] **(C)**
Placement of insulin pump and CGM devices	• Based on activity type and user experience, consider location of the infusion set and CGM placement in areas less likely to fail (e.g. fall off) [4] **(D)** • Consider additional adhesives or overlay tape to protect devices during activity [4] **(D)** • Consider placing the insulin infusion set further away from actively working skeletal muscle **(consensus D)**
Blood glucose and blood ketone monitoring	• Have a glucose meter readily available (e.g. in case of CGM failure or malfunction, to confirm sensor glucose level) [4, 14, 15, 17, 19] **(D)** • Have a blood ketone meter (or urine ketone strips) available if the PA is prolonged or intense [4] **(D)**

Overall, a challenge of AID systems around PA is preventing an increase in algorithm-derived automated insulin delivery before the onset of exercise when the sensor glucose value is rising or already elevated because of a CHO snack or a reduction in prandial insulin delivery before the activity. However, if a higher glucose target is set prior to performing a manual prandial (bolus) insulin reduction and/or consuming an ‘uncovered snack’ (i.e. a snack without prandial insulin administered), this effect is likely to be attenuated. Another challenge during PA and instances of hyperglycaemia is that some AID systems do not restrain insulin delivery effectively enough and/or they continue to give automatic insulin correction doses during PA, even if a higher glucose target is set. While we recommend keeping the AID system in automated mode during PA, in cases where the device still results in PA-related hypoglycaemia, we acknowledge that placing the AID system in manual mode before the activity begins may be necessary. We also acknowledge that suspending with or without disconnecting the AID system may be required in some settings, which may require an alternative insulin delivery method (such as taking insulin by injection or by reconnecting the insulin pump and giving a manual bolus intermittently).

Strategies for glucose management around PA with AID systems may also differ based on the timing and nature of the activity and whether that activity is planned or unplanned (Tables 1, 2 and 3). For example, following an overnight fast or before a high-intensity sprint activity, a higher glucose target may be set close to the start of PA or may not be necessary at all [21–24] **(D)**; however, more research in this area is warranted [21]. For planned PA after a meal (up to 2 h after a meal), a higher glucose target should be set first, where possible, followed by performing a prandial bolus insulin reduction (e.g. around 25–33% reduction) to help reduce prandial insulin on board (IOB) and the risk of hypoglycaemia (see ESM 1 for more details on IOB). In situations where the planned activity occurs more than 2 h after a meal, the higher glucose target should be set between 1–2 h beforehand and maintained until the end of the activity [25, 26] **(A)**. For unplanned activity, AID systems may provide some protection against exercise-induced hypoglycaemia relative to other insulin delivery modalities when basal insulin delivery is fixed, but CHO intake is typically required, and to a greater extent, compared with planned activity [27]. As such, a recommendation for unplanned activity is still to set a higher glucose target from the start until the end of activity.

**Table 2:** General AID strategies for planned PA based on pre-exercise starting glucose concentrations

Before PA (1–2 h before)		During PA (every 20–30 min)	After PA^a^
Glucose level	Strategy		
>15.0 mmol/l or Previous history where increase in glucose expected during PA	• May not need higher glucose target • Usual prandial insulin dose before PA • If glucose >15.0 mmol/l (and symptoms of DKA exist), check infusion site for any noticeable causes of blockages, such as kinked tubing or pressure/discomfort at infusion site, and test for ketones	• If glucose <7.0 mmol/l, consume 3–20 g CHO depending on CGM trend arrow	• If glucose >15.0 mmol/l after PA, resume usual settings and continue to monitor ketones and glucose levels
5.0–15.0 mmol/l	• Start higher glucose target^b^ • If PA occurs <2 h after meal, perform 25–33% prandial bolus reduction		• If glucose 5.0–15.0 mmol/l after PA, resume usual settings
<5.0 mmol/l or Previous history where decrease in glucose expected during PA	• Start higher glucose target^b^ • If PA occurs <2 h after meal, perform 25–33% prandial bolus reduction • CHO (10–20 g) snack at PA onset with no prandial insulin		• If glucose <5.0 mmol/l, consider 3–20 g CHO and maintain higher glucose target for up to 2 h post PA

^a^Consider 25–33% reduction in prandial bolus insulin with the following meal after PA. For unplanned PA, the frequency of CHO intake is generally expected to be higher and closer to the upper limit of CHO amount recommendations than that for planned PA

^b^If PA occurs after a meal where a prandial bolus dose is delivered, set the higher glucose target before performing the prandial bolus reduction

CGM, continuous glucose monitoring **(A–D)**; DKA, diabetic ketoacidosis

**Table 3:** General AID strategies for unplanned PA based on pre-exercise starting glucose concentrations

At PA onset		During PA (every 20–30 min)	After PA^a^
Glucose level	Strategy		
>15.0 mmol/l or Previous history where increase in glucose expected during PA	• May not need higher glucose target • If glucose >15.0 mmol/l (and symptoms of DKA exist), check infusion site for any noticeable causes of blockages, such as kinked tubing or pressure/discomfort at infusion site, and consider testing for ketones	• If glucose <7.0 mmol/l, consume 3–20 g CHO depending on CGM trend arrow	• If glucose >15.0 mmol/l after PA, resume usual settings and continue to monitor ketones and glucose levels
5.0–15.0 mmol/l	• Start higher glucose target until end of PA^b^ • May consider CHO (10–20 g) snack at PA onset with no prandial bolus insulin		• If glucose 5.0–15.0 mmol/l after PA, resume usual settings
<5.0 mmol/l or Previous history where decrease in glucose expected during PA	• Start higher glucose target until end of PA^b^ • CHO (10–20 g) snack at PA onset with no prandial bolus insulin		• If glucose <5.0 mmol/l, consider 3–20 g CHO and maintain higher glucose target for up to 2 h post PA

^a^Consider 25–33% reduction in prandial bolus insulin with the following meal after PA. For unplanned PA, the frequency of CHO intake is generally expected to be higher and closer to the upper limit of CHO amount recommendations than that for planned PA

^b^If PA occurs after a meal where a prandial bolus dose is delivered, set the higher glucose target before performing the prandial bolus reduction

CGM, continuous glucose monitoring **(A–D)**; DKA, diabetic ketoacidosis

If glucose levels drop below 7.0 mmol/l during PA, even with a higher glucose target set, we recommend that small amounts of fast-acting CHO be consumed based on the CGM trend arrow (see below), without announcing it to the AID system [7, 28–31] **(C)**:

- 3–6 g for a horizontal trend arrow
- 6–9 g for a slightly decreasing trend arrow
- 9–12 g for a decreasing trend arrow
- 12–20 g for two or three decreasing trend arrows

We also recommend checking the sensor glucose around 20–30 min after CHO consumption and repeating treatment if necessary [4] **(D)**. For the post-exercise period, the higher glucose target should be stopped at the end of PA [32, 33], however, exceptions to this rule may exist, such as after an unusually active day or when post-exercise late onset hypoglycaemia persists [31, 34] **(C)**.

## AID-specific recommendations

AID-specific recommendations are listed alphabetically by company.

### Beta Bionics iLet Bionic Pancreas

The iLet insulin-only Bionic Pancreas system’s glucose targets can be set to 6.1 (lower), 6.7 (usual) or 7.2 mmol/l (higher). Unlike other AID systems, the iLet is initialised based only on bodyweight and does not require discrete CHO input for meals; instead, users employ a qualitative approach to meals indicating if meal sizes are ‘Usual for me’, ‘More’ or ‘Less’. The system delivers ~75% of the estimated insulin needs for a meal immediately and will automatically increase or decrease additional basal or correction insulin dosing in the postprandial period as needed. Correction doses are provided by the system and the user cannot override it to give a manual dose of insulin. In this AID system, IOB is estimated using a fixed model of insulin absorption into the blood and clearance from the blood that considers all correction boluses and meal boluses. IOB is computed every 5 min based on an assumed peak time of insulin in the blood after administration (tmax) of 65 min. Unlike most other AID systems, the duration of insulin action cannot be adjusted by the user.

#### Evidence on glucose management around PA with the iLet system

**Fig. 2:**
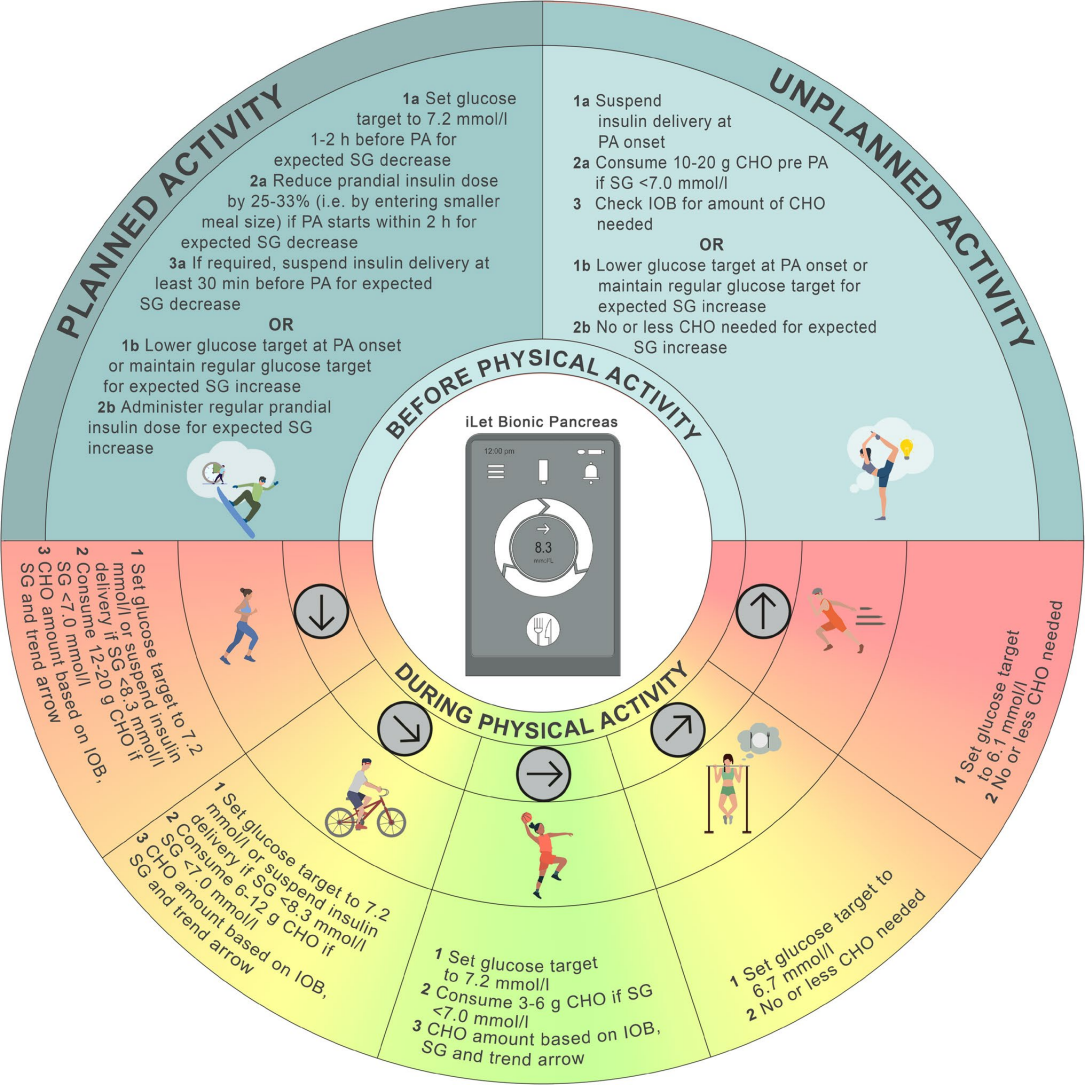
Recommendations for use of the iLet Bionic Pancreas system to manage glucose outcomes during PA. Consider insulin delivery suspension with or without disconnecting the iLet system 30 min prior to activity to help mitigate hypoglycaemia risk. If ingesting undeclared CHO and disconnecting the iLet before activity, ensure that the device is already suspended and disconnected prior to CHO ingestion. The prandial bolus insulin dose can be reduced only by ‘underestimating’ CHO (i.e. entering a smaller meal size). SG, sensor glucose. Glucose values: 6.1 mmol/l = 110 mg/dl, 6.7 mmol/l = 120 mg/dl, 7.0 mmol/l = 126 mg/dl, 7.2 mmol/l = 130 mg/dl, 8.3 mmol/l = 150 mg/dl. See ESM 1 for version of this figure with glucose concentrations in mg/dl. This figure is available as part of a downloadable slideset

To date, the iLet system has been tested only in clinical trials of physically active youth and adults with type 1 diabetes, with no formal evaluation of how it performs during and after PA [35, 36]. Nonetheless, several exercise studies on earlier system designs, including dual-hormone (i.e. glucagon and insulin) configurations, have been conducted [37–39]. Therefore, recommendations (Fig. 2) are given based primarily on studies from other AID systems with considerations on how the iLet system may be adjusted for PA (all recommendations are level **D**).

#### Recommendations for glucose management around PA with the iLet system

The iLet system currently does not have a feature to allow a higher glucose target to be set prior to PA to help reduce the risk of hypoglycaemia during an activity. However, if using a glucose target of 6.1 or 6.7 mmol/l as the ‘usual target’, one option for PA may be to switch the glucose target to 7.2 mmol/l, ideally 1–2 h before the activity **(consensus D)**. Users must remember to return the glucose target back to the usual target following PA. The prandial bolus insulin dose can be reduced for a pre-exercise meal bolus only by entering a smaller meal size into the device (i.e. select ‘Less’ rather than ‘Usual for me’), which effectively reduces the bolus insulin dose by 50%.

One other point to consider is whether to leave the iLet connected during PA or whether it should be suspended with or without disconnecting the pump during some PA where the risk for hypoglycaemia is elevated. This approach may need to be personalised to the individual and the PA type and intensity **(consensus D)**. Without the current option of setting a glucose target >7.2 mmol/l, in instances of increased hypoglycaemia risk, key strategies for this AID system around PA include (1) frequent checking and monitoring of real-time CGM values and trends pre, during and post exercise; (2) having fast-acting CHO readily available to prevent or treat hypoglycaemia; and (3) aiming to limit the amount of CHO on board before PA when possible, to avoid increases in automated insulin delivery **(consensus D)**. For individuals aiming to consume uncovered CHO before PA, one strategy is to consume CHO after suspending and disconnecting the iLet system, to avoid increases in automated insulin delivery **(consensus D)**.

### CamDiab mylife CamAPS FX

The mylife CamAPS FX system allows a glucose target between 4.4 and 11.0 mmol/l to be set, with a default target of 5.8 mmol/l. Insulin delivery using auto-modulated insulin release based on the algorithm and manual correction doses is possible in auto-mode but is not recommended unless following an infusion set occlusion or similar.

In this system, any bolus insulin given through the bolus calculator (correction or meal related) counts towards IOB (displayed as ‘Active Insulin’). The active insulin time that is displayed to the user can be set between 2 and 8 h; however, the real active insulin time used by the algorithm is subject to adaptive learning and is automatically adjusted. Basal rate or algorithm-directed insulin delivery does not count towards IOB, and the programmed duration of insulin action does not affect the algorithm-directed insulin delivery. A realistic view of IOB can be visualised by turning the mobile phone to landscape (horizontal mode), which allows the last bolus dosing and pharmacokinetic profile of the basal rate of insulin delivery to be seen.

Two additional features are available in the mylife CamAPS FX system: the ‘Ease-off’ mode, which delivers less insulin, raises the glucose target and suspends insulin delivery if glucose levels are <7.0 mmol/l; and the ‘Boost’ mode, which increases the algorithm responsiveness to higher glucose levels by up to ~35% while maintaining the same glucose target.

#### Evidence on glucose management during PA with the mylife CamAPS FX system

Previous studies performed in children and adolescents with type 1 diabetes using the mylife CamAPS FX system demonstrated that use of the Ease-off mode for PA resulted in safe glucose levels during PA [31, 34]. Specifically, increasing the glucose target to 8.3 mmol/l and simultaneously starting the Ease-off mode 2 h before maximum cardiopulmonary exercise testing resulted in stable glucose levels in young people with type 1 diabetes (start 10.7±3.1 mmol/l vs end 10.5±3.1 mmol/l; *p*=0.69) [34]. In a ski camp study performed in children and adolescents with type 1 diabetes, it was also shown that starting the Ease-off mode 2 h before exercise was suitable for avoiding hypoglycaemia [31].

#### Recommendations for glucose management around PA with the mylife CamAPS FX system

**Fig. 3:**
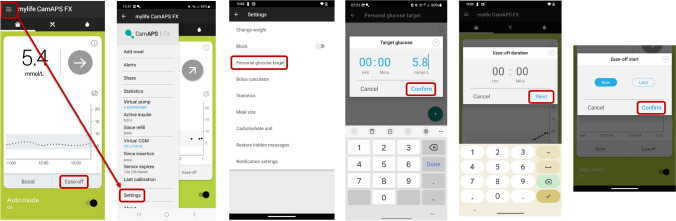
Illustration of how to set a new personal glucose target and how to set the Ease-off (now or later) mode when using the mylife CamAPS FX system. See ESM 1 for version of this figure with glucose concentrations in mg/dl. This figure is available as part of a downloadable slideset

To reduce the risk of hypoglycaemia during activity, we suggest setting the Ease-off mode and/or increasing the glucose target 1–2 h before PA [31, 34] **(C)**; this may be especially relevant in instances of high IOB or during aerobic exercise [40] **(D)**. We recommend using the Boost mode if an increase in glucose is expected during PA [8] **(D)** (e.g. during high-intensity sprinting in the fasted state [41] **(D)**). If deemed useful by the user, caregiver or healthcare professional, both the Ease-off and the Boost mode can be pre-programmed in this system to automatically start and end at a predefined time when PA is expected, as described in Fig. 3.

For unplanned, low- to moderate-intensity PA, where a decrease in glucose levels is expected and the glucose level is already in a reasonable target range for PA (e.g. 5.0–7.0 mmol/l), the Ease-off mode and/or a higher glucose target should be set immediately, followed by consumption of 10–20 g of CHO at exercise onset [7, 42] **(D)**. The suggestion is to announce this meal or snack as ‘hypoglycaemia treatment’ in the ‘Add meal’ function and not as a regular meal, otherwise the system will likely deliver insulin [31, 34] **(C)**. As with any AID system, more CHO can be consumed during prolonged PA based on observed glucose trends and for performance reasons [43]. In contrast, for instances where a rise in glucose levels is expected during PA (e.g. during high-intensity PA in the overnight fasted state) [41], we recommend starting the Boost mode with the regular or lower glucose target at the onset of PA to help limit activity-related hyperglycaemia. We advise not starting the Boost mode well in advance of the onset of the activity, as this might result in pre-exercise hypoglycaemia **(consensus D)**.

**Fig. 4:**
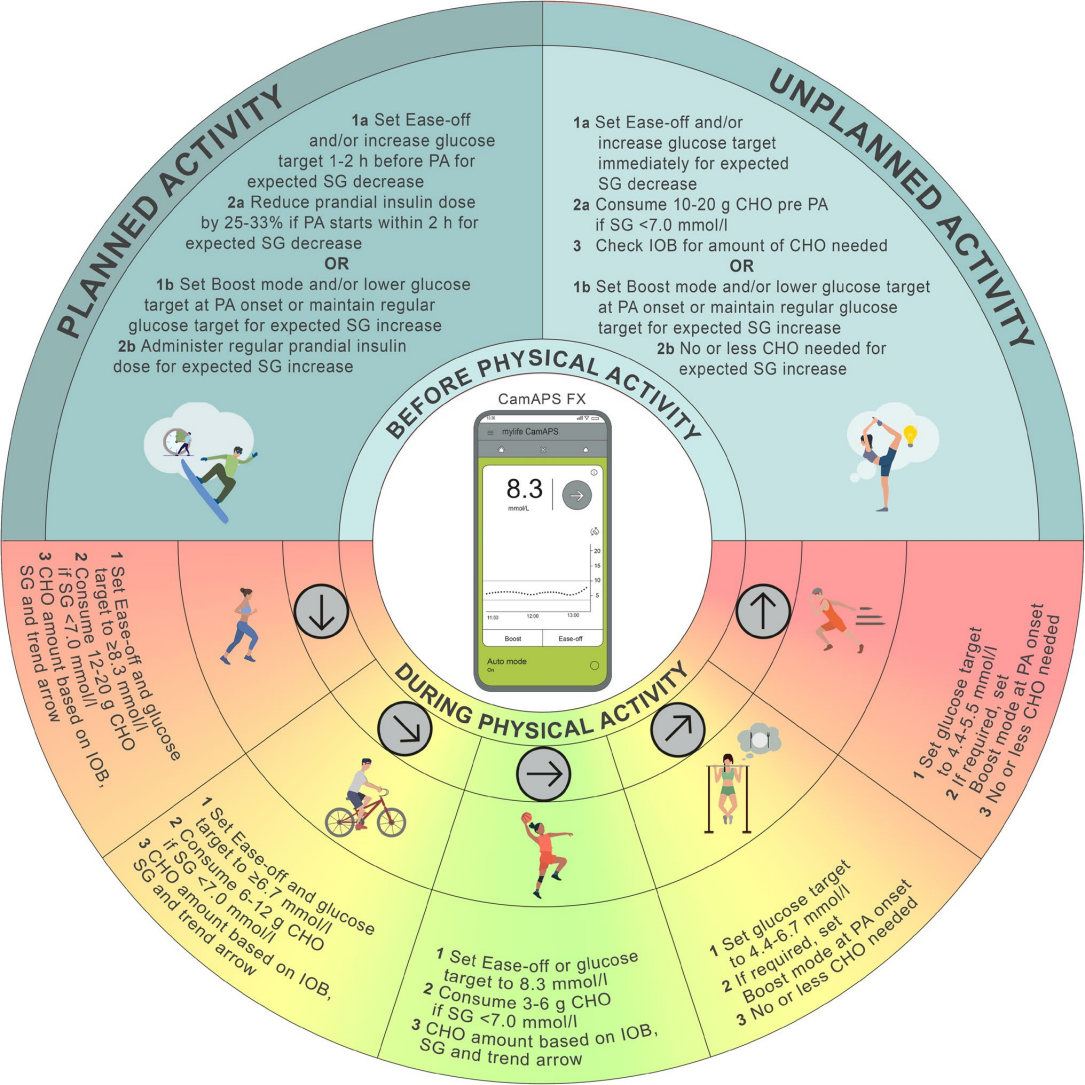
Recommendations for use of the mylife CamAPS FX system to manage glucose outcomes during PA. Insulin delivery suspension with or without disconnection for prolonged periods (up to 120 min) may be required under some circumstances (e.g. swimming, diving, contact sports), although it is generally not recommended for most activities. SG, sensor glucose. Glucose values: 4.4 mmol/l = 80 mg/dl, 5.6 mmol/l = 100 mg/dl, 6.7 mmol/l = 120 mg/dl, 7.0 mmol/l = 126 mg/dl, 8.3 mmol/l = 150 mg/dl. See ESM 1 for version of this figure with glucose concentrations in mg/dl. This figure is available as part of a downloadable slideset

As the Ease-off and Boost modes contribute to a lesser extent to the algorithmic learning, these modes may be considered for individuals who perform more irregular PA **(consensus D)**. For individuals who exercise more regularly with respect to specific days and times (e.g. Mon, Wed, Fri and Sun at ~17:00), one option may be to set a specific glucose target depending on the time of day and type of PA that is typically performed, as described in Fig. 3**(consensus D)**. For example, when glucose levels are expected to decrease during PA, consider setting a glucose target ≥8.3 mmol/l ~2 h before activity. We also recommend that users set an individualised glucose target for PA when they are using either the Ease-off mode (i.e. higher glucose target) or the Boost mode (i.e. lower glucose target) to help achieve their desired glucose level [34] **(D)**. All recommended adaptations concerning the Ease-off and Boost modes, as well as glucose targets, are provided in Fig. 4.

### Diabeloop Generation 1

The Generation 1 (DBLG1) system’s default glucose target is 6.1 mmol/l, but the glucose target can be set between 5.6 and 7.2 mmol/l. The low glucose threshold when insulin delivery is stopped can be set between 3.3 and 4.7 mmol/l and the algorithm hyperglycaemia threshold is 10.0 mmol/l. The aggressiveness of insulin delivery of the DBLG1 system can be modified to deliver 59–147% of the typical basal rate when glucose is between 3.9 and 10.0 mmol/l. When sensor glucose is >10.0 mmol/l, the automated correction bolus can be set to deliver within the range of 43–186% of the typical automated correction bolus dose. The prandial insulin dose can also be set to deliver insulin in the range of 50–200% for breakfast, lunch and dinner. These functions may be used to adapt the prandial insulin dose for post-meal activity; however, this needs to be discussed, individualised and, in some cases, modified with support from the healthcare professional team.

In this system, IOB (displayed as ‘Active Insulin’), as shown in the interface, corresponds to the IOB provided by regulation, including any insulin source confirmed by the pump (basal rate and bolus insulin).

#### Evidence on glucose management during PA with the DBLG1 system

In a post hoc analysis of an RCT, glycaemic outcomes were compared between days with and days without PA in 56 adults with type 1 diabetes using the DBLG1 system for 12 weeks [44]. Participants announced PA at least 30 min before exercise, which reduced insulin delivery, and, if necessary, a certain amount of CHO was also recommended by the system to avoid hypoglycaemia. Time below range (<3.0 mmol/l; TBR<3.0) was not significantly different between days with and days without PA, independent of exercise duration and intensity (2.0±1.5% vs 2.2±1.1%; *p*>0.05). Ingested CHO as a preventative strategy against hypoglycaemia as recommended by the system were significantly higher on days with PA (41.1±35.5 vs 21.8±28.5 g/day; *p*<0.001), and the AID insulin dose was significantly lower on days with PA (31.5±10.5 vs 34.0±10.5 U/day; *p*<0.001). The time above range (>10.0 mmol/l; TAR>10.0) was 28.7±9.3% on days with PA compared with 26.8±8.6% on days without PA (*p*=0.017). Time in range (3.9–10.0 mmol/l; TIR3.9–10.0) was 69.1±8.2% on days with PA vs 70.9±8.2% on days without PA (*p*=0.017). The coefficient of variation in glucose was higher on days with PA than days without (32.0±3.7% vs 30.9±3.7%; *p*=0.019), indicating increased glycaemic variability on exercise days.

Another study performed in adults with type 1 diabetes showed that the DBLG1 system was superior to open-loop insulin delivery with respect to TIR3.9–10.0 and TAR>10.0 when the ‘Physical Activity’ mode was set 30 min before the start of activity [45].

#### Recommendations for glucose management around PA with the DBLG1 system

**Fig. 5:**
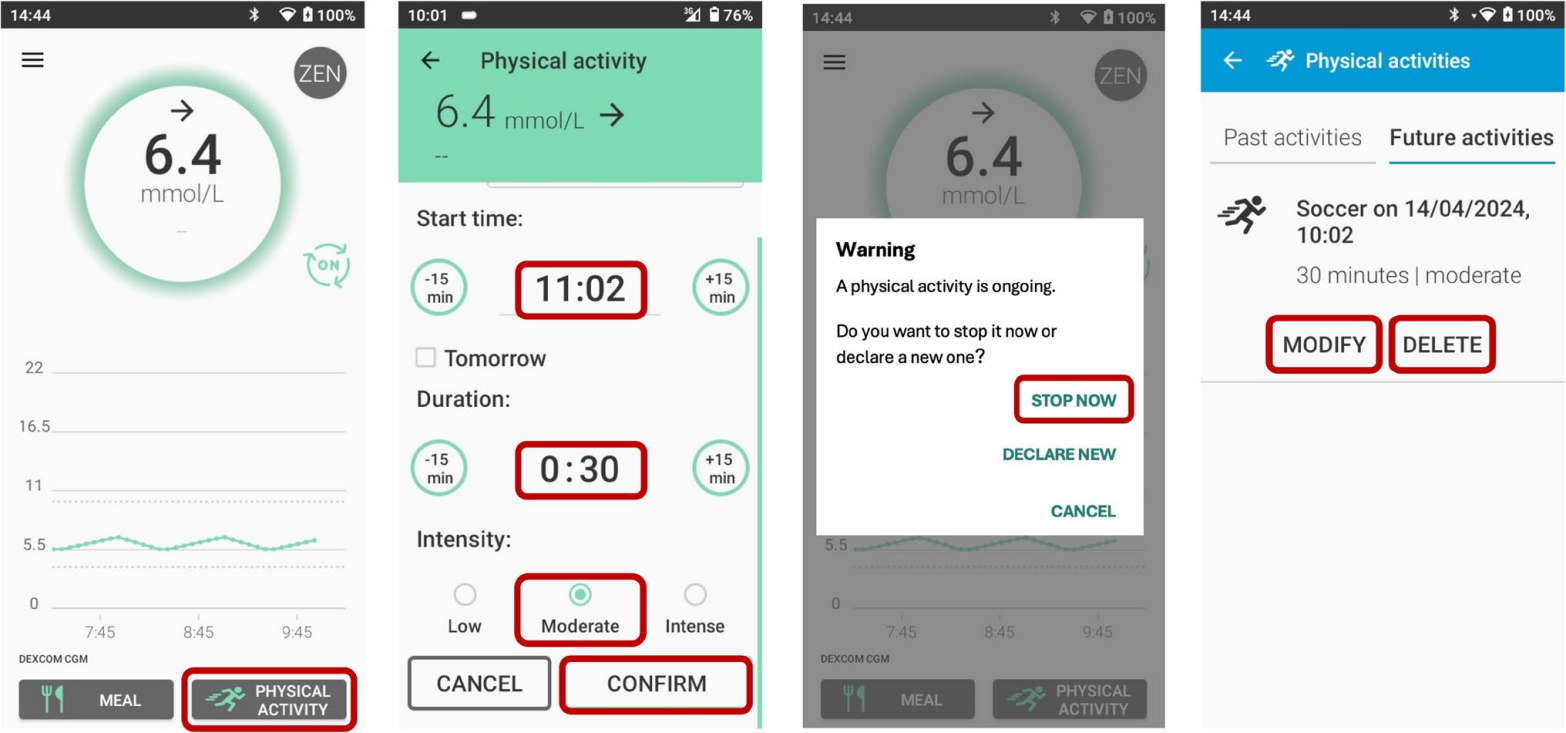
Illustration of how to start, stop and modify/delete the Physical Activity mode in the DBLG1 system. See ESM 1 for version of this figure with glucose concentrations in mg/dl. This figure is available as part of a downloadable slideset

The Physical Activity mode can be used to decrease the risk of hypoglycaemia during PA (Fig. 5). In this mode, the glucose target and hypoglycaemia threshold are increased by 3.9 mmol/l, which reduces the aggressiveness of insulin delivery. When the Physical Activity mode is used, the PA intensity can be set to low, moderate or intense and the planned duration of PA can be set. Both the duration and intensity are considered as a matrix, with coefficients modulating the insulin basal rate, corrective bolus or meal bolus. Another feature of the DBLG1 system is the ‘ZEN’ mode, which increases the glucose target by an increment that is between 0.6 and 2.2 mmol/l for a period of 1–8 h [8] **(D)**.

**Fig. 6:**
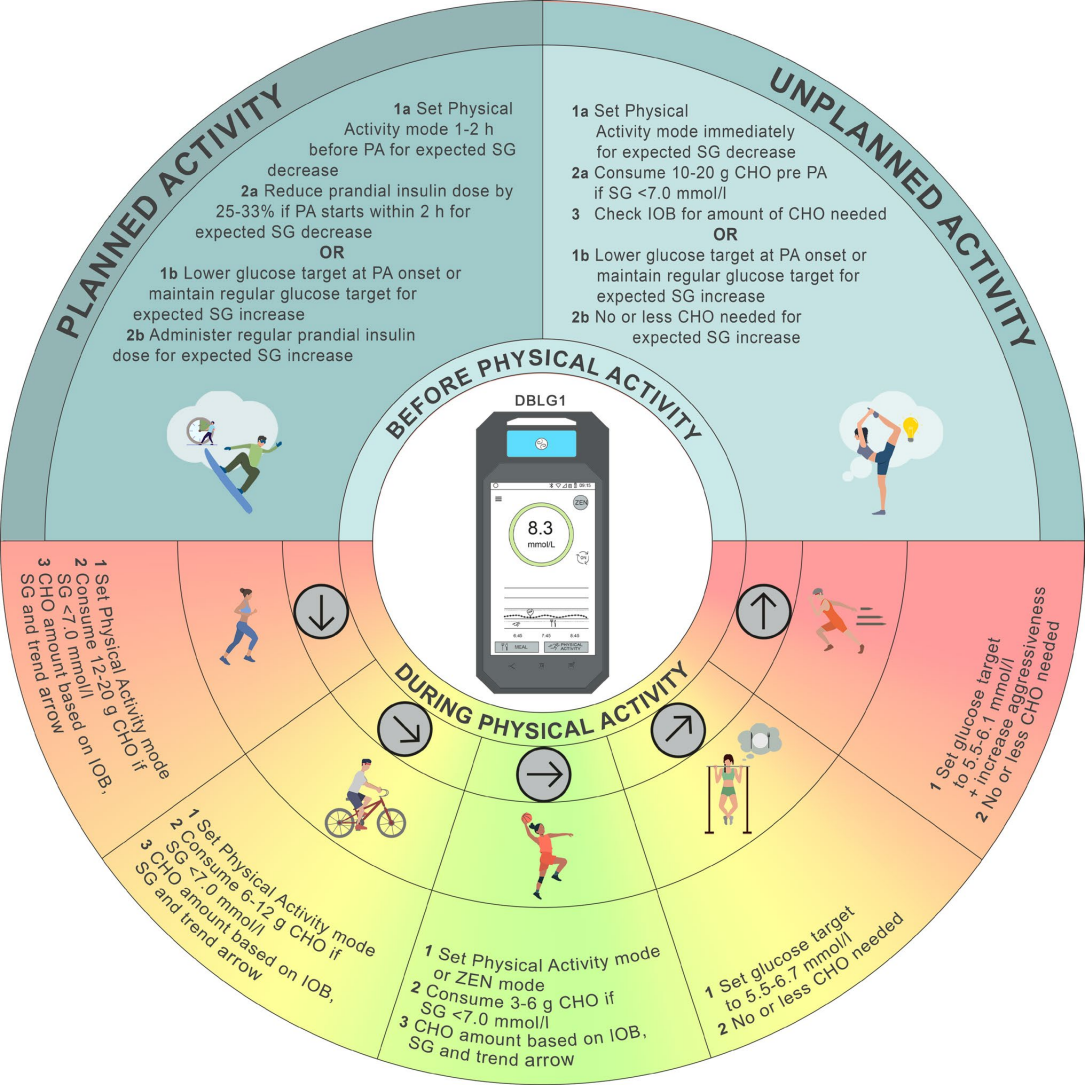
Recommendations for use of the DBLG1 system to manage glucose outcomes during PA. Insulin delivery suspension with or without disconnection for prolonged periods (up to 120 min) may be required under some circumstances (e.g. swimming, diving, contact sports), although it is generally not recommended for most activities, as several of these strategies cannot be implemented and/or require modification. SG, sensor glucose. Glucose values: 5.6 mmol/l = 100 mg/dl, 6.1 mmol/l = 110 mg/dl, 6.7 mmol/l = 120 mg/dl, 7.0 mmol/l = 126 mg/dl. See ESM 1 for version of this figure with glucose concentrations in mg/dl. This figure is available as part of a downloadable slideset

We recommend starting Physical Activity mode at least 30 min before PA, as the DBLG1 system suggests consuming a specific amount of CHO to avoid hypoglycaemia [44] **(C)**. However, it is also beneficial to start Physical Activity mode earlier (e.g. between 1 and 2 h before the start of PA), as this has been shown to reduce the risk of hypoglycaemia (Fig. 6) [31, 46] **(D)**. When PA is announced more than 1 h before the start of the activity, the target glucose is increased by 3.9 mmol/l and the system aims to raise blood glucose prior to the start of PA. However, if glucose levels are <8.9 mmol/l 15 min before the start of PA, a specific CHO intake is recommended by the system. When PA is announced closer to the start of the activity, the system only provides a recommendation for CHO intake 15 min before PA if glucose is <8.9 mmol/l.

Furthermore, the DBLG1 system automatically reduces the basal rate of insulin delivery for 16 h after Physical Activity mode is enabled to help mitigate the risk of post-exercise hypoglycaemia caused by increased insulin sensitivity. Physical Activity mode also allows the user to name and save the PA session (e.g. football) and provide the duration and intensity (e.g. low, moderate, intense). Fig. 6 provides recommendations for managing glucose levels during PA using the DBLG1 system.

### Insulet Omnipod 5

The Omnipod 5 system is a tubeless AID system [8, 47] that uses SmartAdjust technology to predict glucose values 60 min in advance and dynamically adjusts basal insulin delivery every 5 min. SmartAdjust targets glucose levels between 6.1 and 8.3 mmol/l, with levels set by the user, caregiver or healthcare professional. Different targets can be programmed for different hours of the day. With each Pod change, usually occurring at least every 72 h, the Omnipod 5 system automatically calculates an adaptive basal rate based on a fading memory of insulin requirements over 6 days. Furthermore, Omnipod 5 is a waterproof patch pump (i.e. Pod) that can provide users with increased flexibility in daily activities, in particular, with water-based activities [48].

In this system, the IOB is the sum of the correction IOB (insulin remaining in the body from previous correction doses), meal IOB (insulin remaining in the body from previous meal boluses) and Omnipod 5 software IOB (i.e. all insulin delivered by the system). IOB is mainly determined by the ‘Duration of Insulin Action’ setting, which ranges from 2 to 6 h. Furthermore, the ‘Reverse Correction’ feature deducts the IOB from the bolus calculation when the current glucose value is below the target glucose value [47].

#### Evidence on glucose management during PA with the Omnipod 5 system

In the pivotal trial of the Omnipod 5 system, an exercise study was conducted in 59 adults with type 1 diabetes. Participants underwent three, 60 min moderate-intensity treadmill exercise sessions in which (1) the ‘Activity’ feature (higher glucose target) was set 30 min prior to exercise; (2) the Activity feature was set 60 min prior to exercise; and (3) usual automated insulin delivery was continued with no adjustment made for exercise [49]. Not surprisingly, at the start of exercise in sessions (1) and (2), insulin delivery was lower and glucose was higher with use of the Activity feature than with usual automated insulin delivery.

#### Recommendations for glucose management around PA with the Omnipod 5 system

**Fig. 7:**
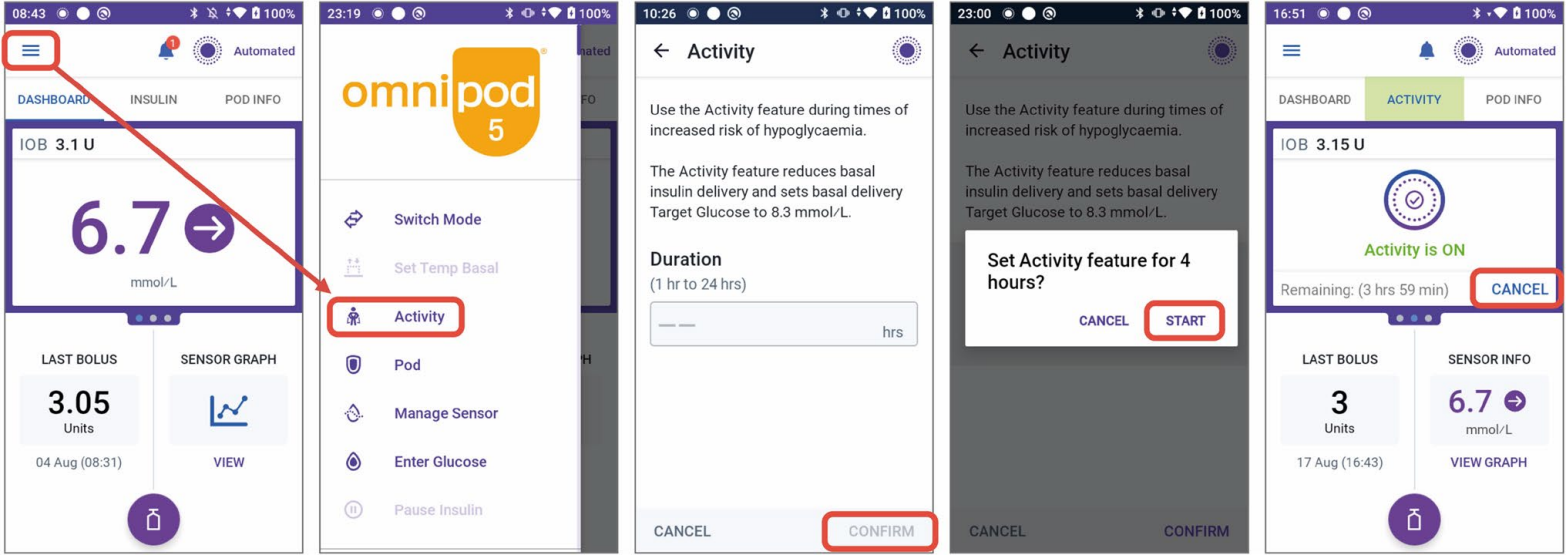
Illustration of how to set and cancel the Activity feature on the Omnipod 5 system. See ESM 1 for version of this figure with glucose concentrations in mg/dl. This figure is available as part of a downloadable slideset

For PA, the higher glucose target in the Omnipod 5 system is 8.3 mmol/l. This target attenuates automated insulin delivery and can be programmed to last from 1 to 24 h [6]. For activities that lead to an increased risk of hypoglycaemia, the recommendation with this system is to set the Activity feature 1–2 h before PA until the end of the activity (Fig. 7).

**Fig. 8:**
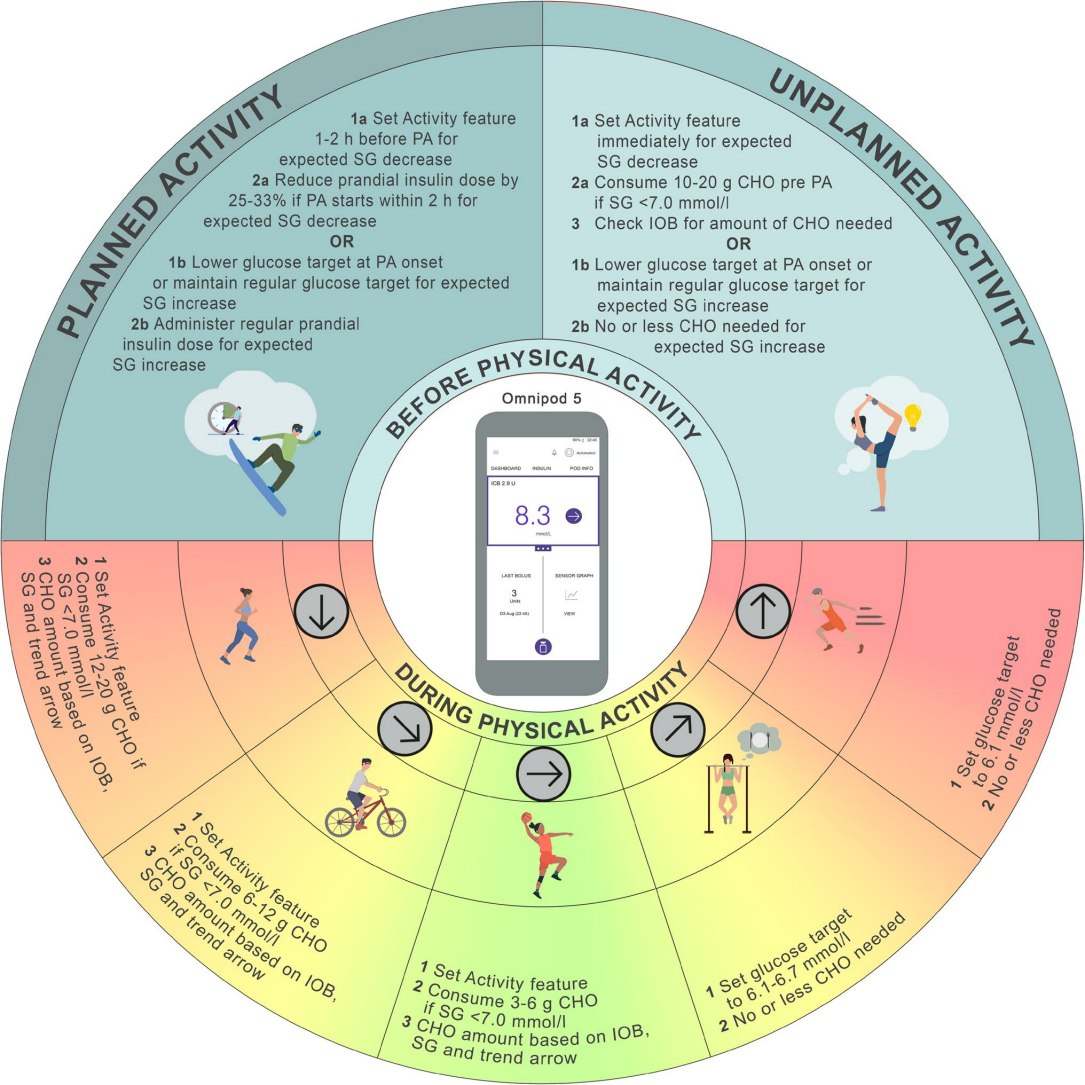
Recommendations for use of the Omnipod 5 system to manage glucose outcomes during PA. SG, sensor glucose. Glucose values: 6.1 mmol/l = 110 mg/dl, 6.7 mmol/l = 120 mg/dl, 7.0 mmol/l = 126 mg/dl. See ESM 1 for version of this figure with glucose concentrations in mg/dl. This figure is available as part of a downloadable slideset

If the usual glucose target is set to 6.7, 7.2, 7.8 or 8.3 mmol/l, and glucose is expected to increase during PA (e.g. fasted, high-intensity PA), we recommend lowering the usual glucose target to 6.1 mmol/l prior to the onset of PA and resuming the usual glucose target after the PA event **(consensus D)** (Fig. 8). With Omnipod 5, up to eight different targets can be programmed throughout the day, so there is some flexibility around what glucose target is set and when. Therefore, for school-aged children, higher glucose targets can be leveraged to account for usual after-school sports by setting the target higher 1–2 h prior to the scheduled activity until the end of the activity **(consensus D)**.

A more general consideration for healthcare professionals is how the ‘Reverse Correction’ feature might impact insulin delivery at the meal before PA. With the Reverse Correction feature on, the prandial bolus dose will be reduced if the pre-meal glucose level is below target. If this feature is combined with a manual prandial bolus insulin reduction initiated by the user (e.g. 25–33% reduction) prior to activity, then glucose will likely rise and result in automated insulin delivery by the system, thereby increasing hypoglycaemia risk during PA. To date, there are no published studies to support specific guidance on using Reverse Correction around PA.

If PA is planned <2 h following a meal and a drop in glucose is anticipated, a 25–33% reduction in prandial bolus insulin is generally recommended [26, 50] **(C)**. Another option is to turn the Reverse Correction feature off when applying a prandial bolus insulin reduction before PA **(consensus D)**. For the meal prior to the onset of PA, the bolus insulin amount on the Omnipod 5 system can be reduced by either (1) entering fewer CHO into the system than the amount being consumed or (2) decreasing the recommended bolus insulin amount by 25% up to 100% (i.e. no bolus) [8]. Importantly, research trials on the amount and timing of prandial bolus insulin reductions before PA in children and adults with type 1 diabetes using Omnipod 5 are not currently available. Fig. 8 provides recommendations for managing glucose levels during PA using the Omnipod 5 system.

### Medtronic MiniMed 780G

The MiniMed 780G system using SmartGuard technology can set glucose targets of 5.5, 6.1, 6.7 and 8.3 mmol/l (‘Temp Target’, exercise mode). One of the major safety features of the Temp Target is the prevention of automatic bolus correction doses in response to rising glucose levels from ingestion of CHO immediately before or during PA. Without this feature, there is likely to be a significant increase in IOB during PA when CHO are given, which can result in a recurrent cycle of hypoglycaemic episodes. The auto-correction bolus, when enabled, automatically delivers bolus insulin doses when the algorithm has been delivering auto-basal insulin at the maximum insulin limit, the sensor glucose value is >6.7 mmol/l and the calculated correction bolus is >10% of the maximum insulin limit. Furthermore, the auto-correction bolus can be switched off in the SmartGuard settings. When SmartGuard technology is used for calculating the bolus insulin dose for CHO, the dose suggestion is increased or decreased based on the actual glucose value and the total IOB.

The IOB (displayed as ‘Active Insulin’) accounts for bolus insulin, including meal boluses, manual correction boluses and automatic correction boluses. Basal insulin, either from a pre-programmed basal rate or from SmartGuard auto-basal insulin delivery, is excluded from active insulin. The displayed IOB is affected by the Active Insulin time settings (2–8 h, adjustable). Active Insulin is also used in the calculation of correction boluses (both manual and automated).

#### Evidence on glucose management during PA with the MiniMed 780G system

The Medtronic MiniMed AID systems are suitable for use during PA in people with type 1 diabetes and are the systems with the largest body of published literature related to PA [7]. In a trial of ten adults with type 1 diabetes, it was shown that transitioning from open-loop systems to the MiniMed 780G system did not significantly alter glucose levels during and after 45 min of moderate-intensity exercise [51].

McCarthy et al demonstrated in adults with type 1 diabetes that glucose levels may be optimised during exercise when using the MiniMed 780G system by reducing the pre-exercise prandial bolus insulin dose by 25% for meals consumed up to 90 min before exercise [26]. This study also showed that increasing the glucose target at the onset of exercise or 45 min prior to the start of exercise was less effective for avoiding hypoglycaemia than setting a higher glucose target 90 min before exercise when prandial insulin was reduced by 25%. In a study of youth with type 1 diabetes using the MiniMed 780G system, it was determined that, independent of the type of insulin used (faster-acting insulin aspart vs standard insulin aspart), exercise was safe, with a TBR (<3.9 mmol/l glucose) of 2.8% vs 2.5%, respectively, when the Temp Target was set at least 1 h before exercise [27].

In a preliminary, controlled, in-clinic research study by Lee et al, TIR3.9–10.0 was 100% for 45 min of high-intensity exercise or moderate-intensity exercise when the Temp Target on the MiniMed 670G system was started 2 h prior to the start of exercise in adults with type 1 diabetes who also had impaired awareness of hypoglycaemia [46]. Use of the MiniMed advanced hybrid closed-loop (AHCL) system with different insulins (faster-acting insulin aspart and insulin aspart) did not significantly alter the risk of nocturnal hypoglycaemia on exercise days compared with non-exercise days [33, 52]. Furthermore, when comparing different types of exercise (high-intensity exercise, resistance exercise, moderate-intensity exercise), there were no differences in glycaemic outcomes [46, 53] or risk of nocturnal post-exercise hypoglycaemia [32].

#### Recommendations for glucose management around PA with the MiniMed 780G system

**Fig. 9:**
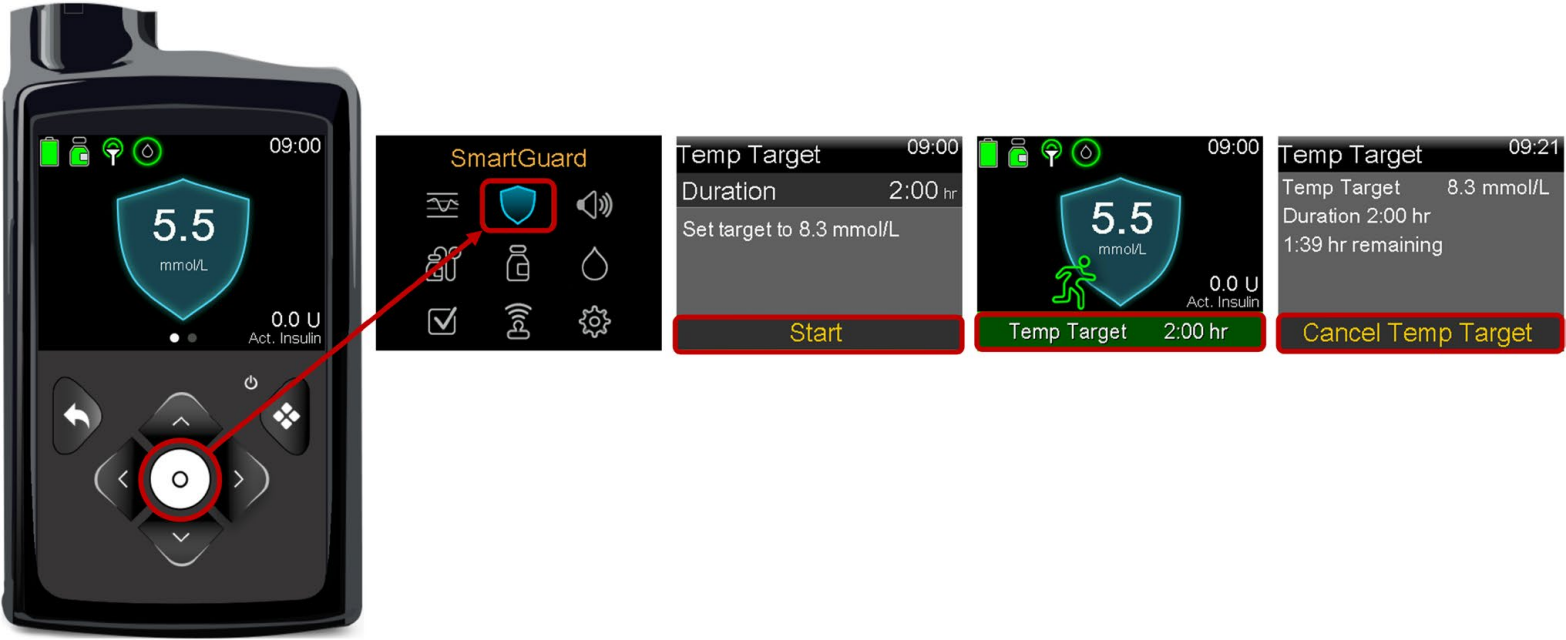
Illustration of how to set and cancel the Temp Target on the MiniMed 780G system. See ESM 1 for version of this figure with glucose concentrations in mg/dl. This figure is available as part of a downloadable slideset

**Fig. 10:**
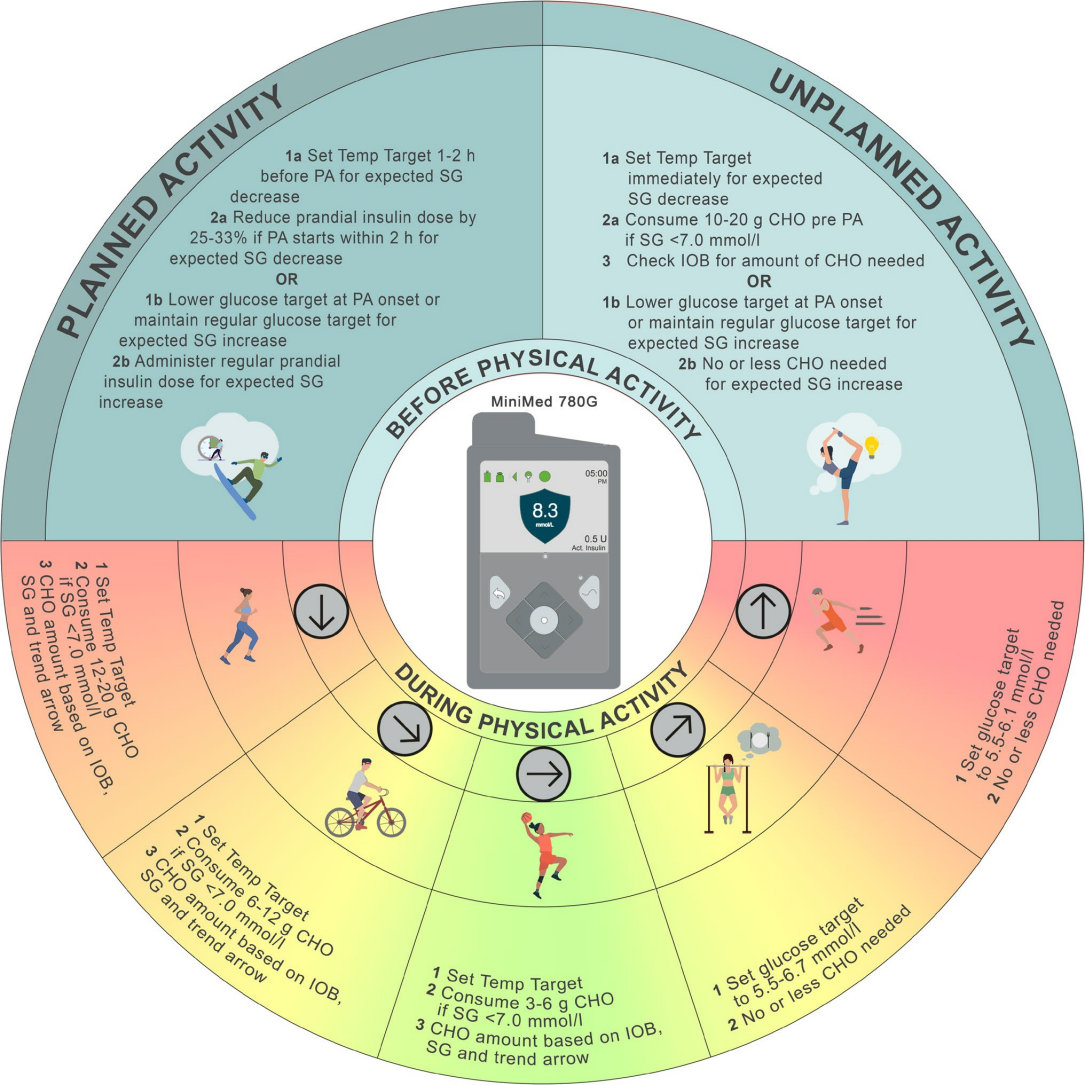
Recommendations for use of the MiniMed 780G system to manage glucose outcomes during PA. Insulin delivery suspension with or without disconnection for prolonged periods (up to 120 min) may be required under some circumstances (e.g. swimming, diving, contact sports), although it is generally not recommended for most activities, as several of these strategies cannot be implemented and/or require modification. SG, sensor glucose. Glucose values: 5.6 mmol/l = 100 mg/dl, 6.1 mmol/l = 110 mg/dl, 6.7 mmol/l = 120 mg/dl, 7.0 mmol/l = 126 mg/dl. See ESM 1 for version of this figure with glucose concentrations in mg/dl. This figure is available as part of a downloadable slideset

When using the MiniMed 780G system, we recommend adjusting the glucose target based on the anticipated glucose response to exercise [53–55] **(D)**. In instances where a glucose decrease is expected during planned PA, one option is to initiate the Temp Target 1–2 h before PA [26, 46]), which will automatically stop after a set duration (Figs 9 and 10). In general, the Temp Target should be timed to stop near the end of the activity [32, 33]. For unplanned PA, when a glucose decrease is expected, CHO consumption (e.g. 10–20 g) will likely be necessary, particularly if glucose levels at exercise onset are <7.0 mmol/l [42] **(C)**. In addition, the Temp Target should be turned on immediately prior to CHO consumption [8] **(D)** (Fig. 10).

If glucose levels are expected to rise during PA, a lower target glucose may be more appropriate (e.g. 5.5 mmol/l), as this will likely result in greater insulin delivery than when a higher target is set. However, setting glucose targets should be based on an individual’s average glucose responses, which may vary depending on PA type, time of day, CHO fuelling strategies, menstrual cycle phase and other factors [56] **(D)** (Fig. 10).

In general, we recommend keeping the MiniMed 780G system in automated mode during PA when a glucose decrease is expected, in addition to setting a Temp Target, and reducing prandial bolus insulin by 25–33% to minimise hypoglycaemia [50, 51].

If there is going to be a reduction in the prandial bolus insulin for the meal preceding PA, this should always be implemented in conjunction with the Temp Target, which disables the automated bolus function. Otherwise, the meal recognition software will signal that food has been eaten and the device will try to address the initial rise in glucose levels with automated bolus insulin.

### Tandem t:slim X2 with Control-IQ

The t:slim X2 insulin pump using Control-IQ technology predicts glucose levels 30 min ahead and adjusts insulin delivery accordingly, including the delivery of automated correction boluses up to once per hour if needed. The ‘Personal Profile’ settings have a standard glucose target of 6.1 mmol/l, but the system targets a glucose range between 6.2 and 8.9 mmol/l. A higher glucose range between 7.8 and 8.9 mmol/l can be set for PA (referred to as ‘Exercise’ mode). In ‘Sleep’ mode, the target shifts to a tighter range (between 6.3 and 6.7 mmol/l), using basal adjustments, and this mode does not perform any auto-correction boluses. While this feature is designed for overnight glucose management, individuals can create sleep schedules at other times of the day to automatically leverage the transition to this tighter target range.

Non-customisable parameters in the Control-IQ system include the duration of active insulin and the glucose target [57]. If Control-IQ technology is enabled, IOB (displayed as ‘Insulin on Board’) includes all basal insulin delivered above and below the programmed basal rate, in addition to all bolus insulin delivered (not adjustable; set to 5 h). Up to six Personal Profiles can be programmed in which the individual can adjust the basal insulin doses, insulin-to-CHO ratio and insulin sensitivity factor settings. Thus, for those performing different types of PA, different settings can be programmed. In addition, a correction dose (up to 60% of the dose determined by the correction factor or insulin sensitivity factor) is delivered a maximum of once per hour if the predicted glucose value 30 min later is anticipated to be >10.0 mmol/l, the system is not in Sleep mode and there has been no user-initiated bolus in the past hour [58]. Recently, a new pump design from Tandem called the Tandem Mobi pump has been released. The Mobi pump uses the Control-IQ algorithm, but has the benefit of a smaller physical footprint and is fully controllable from a user’s phone via a mobile app (with a button available on the pump to permit bolus delivery without the app).

#### Evidence on glucose management around PA with the t:slim X2 Control-IQ system

The Tandem Control-IQ Artificial Pancreas system was the first AID system tested in adolescents and children (aged 6–18 years) with type 1 diabetes in an outpatient exercise setting (i.e. during winter sports, particularly skiing) [59–61]. These real-world studies demonstrated that Control-IQ technology improved glycaemic metrics and reduced hypoglycaemia risk during prolonged winter sporting activities in this group compared with a non-AID system. Mameli et al recently evaluated TIR3.9–10.0 during 2 h of outdoor physical activity, planned 90 min after lunch, in youth aged 9–18 years using the t:slim X2 pump with Control-IQ technology [62]. In this study, group A underwent endurance activities for 60 min (1000 m run, jump circuit) followed by power activities for 60 min (80 m run and a long jump), and group B underwent power activities for 60 min followed by endurance activities for 60 min. A higher glucose target (Exercise mode, see below) was set 90 min before exercise until dinner time and pre-exercise prandial bolus insulin was reduced by 50%. In this study, group A and group B participants had similar TIR3.9–10.0 during the 2 h of activity (50.4%, 95% CI 33.8, 75.0 vs 39.6%, 95% CI 26.9, 58.3; *p*=0.39). No TBR<3.0 occurred during the 2 h of activity (both the endurance and the power workout) [62].

#### Recommendations for glucose management around PA with the t:slim X2 Control-IQ system

**Fig. 11:**
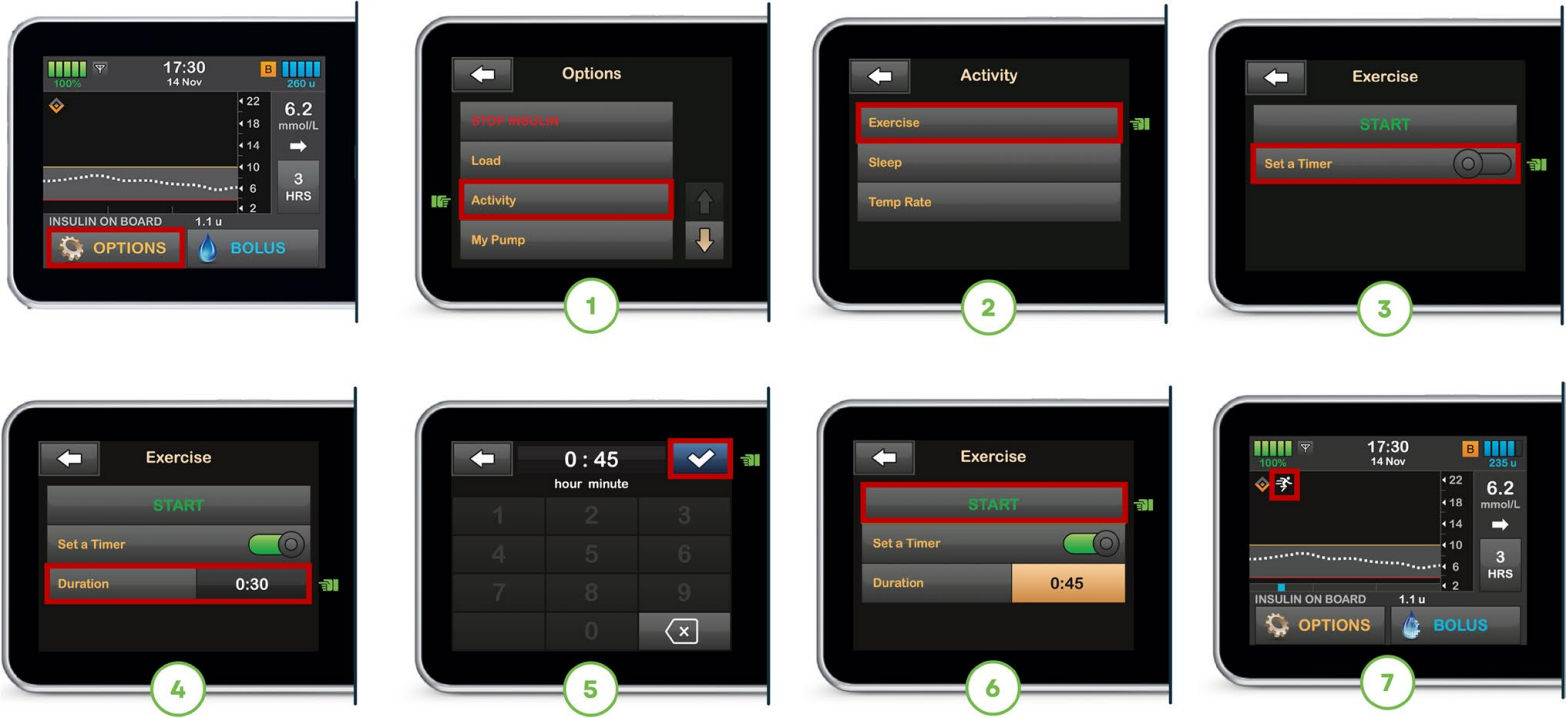
Illustration of how to set the Exercise mode on the t:slim X2 Control-IQ system. See ESM 1 for version of this figure with glucose concentrations in mg/dl. This figure is available as part of a downloadable slideset

Individuals with type 1 diabetes using this system can announce PA using Control-IQ’s Exercise mode, which aims to maintain glucose levels between 7.8 and 8.9 mmol/l (Fig. 11). When using Exercise mode, Control-IQ technology will decrease the algorithm-derived insulin delivery rate (or basal insulin delivery rate) when the glucose level is predicted to be <7.8 mmol/l 30 min in the future and will increase the insulin delivery rate when the glucose is predicted to be >8.9 mmol/l 30 min in the future [6]. If the glucose prediction 30 min in the future is expected to exceed 10.0 mmol/l, an automated correction bolus equivalent to 60% of the dose calculated by the insulin sensitivity factor will be delivered up to once per hour, even if Exercise mode is active [58]. This may increase the risk for activity-related hypoglycaemia in some settings where a rise in glucose occurs prior to PA (e.g. from unannounced CHO intake). Importantly, an automated correction bolus will not occur within 60 min of any bolus (of any amount) that has been delivered or cancelled. With software version 7.7 (introduced in some countries in January 2024), Exercise mode can be set for a duration of 30 min to 8 h. Otherwise, for software versions below version 7.7, the user will need to manually turn Exercise mode off after PA.

If CHO are given during PA to treat pending or actual hypoglycaemia, the rise in glucose from treatment can trigger insulin delivery even during Exercise mode. Based on the current evidence, the general recommendation for this AID system is the same as that for other AID systems that have a higher glucose target for PA: to minimise excessive CHO feeding before and during the activity. We recommend that Exercise mode is enabled 1–2 h before the start of PA until the end of the activity to decrease IOB and reduce the risk of hypoglycaemia during activity [46] **(D)**.

**Fig. 12:**
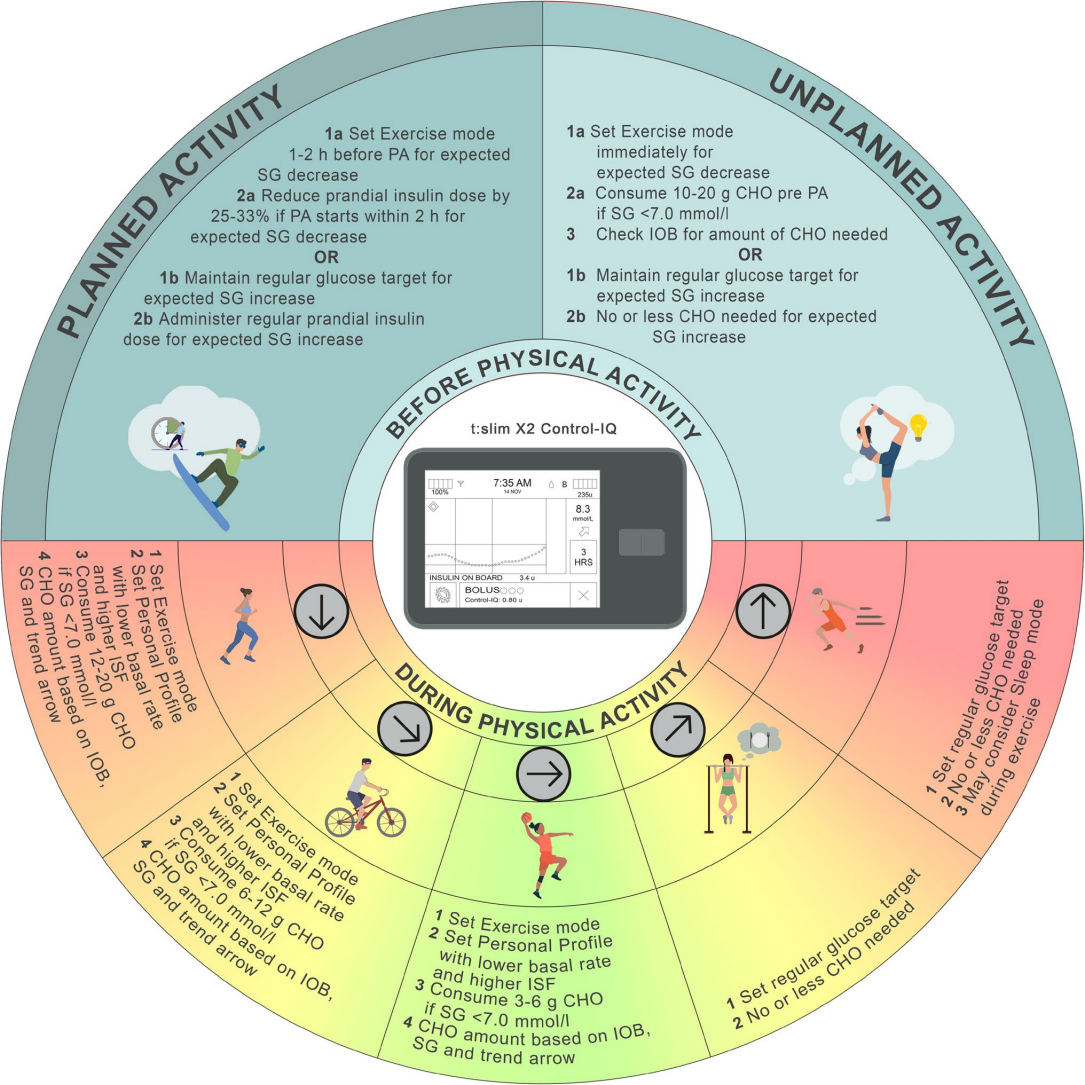
Recommendations for use of the t:slim X2 Control-IQ system to manage glucose outcomes during PA. Consider adding a minimal manual bolus dose (e.g. 0.05 U) close to the onset of exercise to block the system from administering auto-correction doses for the next 60 min. Insulin delivery suspension with or without disconnection for prolonged periods (up to 120 min) may be required under some circumstances (e.g. swimming, diving, contact sports), although it is generally not recommended for most activities, as several of these strategies cannot be implemented and/or require modification. ISF, insulin sensitivity factor; SG, sensor glucose. Glucose value: 7.0 mmol/l = 126 mg/dl. See ESM 1 for version of this figure with glucose concentrations in mg/dl. This figure is available as part of a downloadable slideset

It may also be helpful for individuals with type 1 diabetes who regularly engage in PA and who may benefit from changes to various pump settings for more physically active days or days with more prolonged activity periods to create different Personal Profiles [8] **(D)**. Personal profiles can be optimised in cases where glucose levels are expected to drop or rise during PA, and this may include adjustments to the basal insulin doses, insulin-to-carbohydrate ratio and/or insulin sensitivity factor. For instances where glucose levels are expected to decrease during PA (e.g. walking, running, cycling), a Personal Profile can be created that can reduce insulin delivery (e.g. lower basal insulin doses; higher insulin sensitivity factor; lower insulin-to-carbohydrate ratio), which can be selected when deemed appropriate (Fig. 12).

Based on **consensus D**, another possible strategy for this AID system is to consider adding a small manual bolus insulin dose (note that the minimum bolus is 0.05 U for this system) close to the onset of PA, which then disables the auto-bolus feature for the next 60 min, even if CHO are consumed and a rise in glucose occurs.

In other situations where glucose levels are expected to rise during PA (e.g. during high-intensity sprinting), a different Personal Profile can be created (e.g. higher basal insulin doses, lower insulin sensitivity factor) **(consensus D)**. Another option is to retain the usual AID settings (i.e. do not set ‘Exercise’ mode) or consider putting the pump into Sleep mode for PA and turning Sleep mode off after PA to resume the usual AID settings post-exercise **(consensus D)**. As a reminder, the system has a lower glucose target range in Sleep mode, but it does not deliver automated boluses during this time. As the targets are tighter, Sleep mode may be an option for instances when glucose levels tend to rise with PA, although research around the utility of this approach is warranted **(consensus D)**. Fig. 12 provides recommendations for managing glucose levels during PA using the t:slim X2 Control-IQ system.

## Other considerations for PA

There is a lack of evidence and recommendations on glucose management strategies during PA under special circumstances for people using AID technology [1–4]. To better address some of these unique situations, Table 4 provides a summary of special PA circumstances, important considerations and possible strategies to help individuals with type 1 diabetes using AID systems. For additional details on specific considerations for PA, see ESM 1.

**Table 4:** Summary of special PA circumstances, important considerations and possible strategies for individuals with type 1 diabetes using AID systems **(consensus D)**

Special circumstances	Considerations	Possible strategies to limit PA dysglycaemia **(consensus D)**
Long-duration PA events (e.g. ultramarathon, ironman, triathlon, road bike racing, prolonged trekking/hiking)	• Low insulin delivery • Increased risk of ketones • Risk of hypoglycaemia during exercise • Risk of hyperglycaemia	• Consider regular CHO feeding throughout activity • To prevent insulin deficiency during PA, may avoid higher glucose target for full duration of event • Avoid large, fully uncovered snacks during event that may drive the AID system to increase automated insulin delivery • Monitor CGM closely and consider setting higher alert thresholds on the CGM device where possible
Prolonged insulin pump disconnection during PA (e.g. ≥120 min pump disconnection for contact sports and/or water-based activities as described below)	• Low insulin delivery • Increased risk of ketones • Risk of hyperglycaemia	• Suspend the AID system to inform algorithm of reduced insulin delivery, which will be viewable in data reports • If suspending the AID system, consider reconnecting the pump every hour for small amount of insulin delivery • If disconnected from the AID system, to prevent insulin deficiency during PA, consider reconnecting the pump and delivering ~50% of the ‘usual basal’ dose every hour • May need to switch to manual mode • May consider ‘un-tethered’ approach combining long-acting insulin dose by injection/pen and pump (removed during PA) and/or consider switching to a patch pump
Competition stress (e.g. football game)	• Stress response • Hyperglycaemia before and during event • Delayed hypoglycaemia following event	• Monitor IOB near competition start because the AID system is likely to increase insulin delivery with stress response and rising glucose levels • Stay hydrated • May want to avoid setting higher glucose target 1–2 h before competition • If glucose rises to >15.0 mmol/l with competition stress, consider a manual partial bolus correction (e.g. 50% of usual correction dose)
Water-based activities (e.g. swimming, surfing, scuba diving)	• Lack of device communication between CGM and AID controller (little to no automation) • Hypoglycaemia must be strictly avoided for safety	• For the Omnipod 5 system, consider keeping the Pod and sensor closer together to permit increased Bluetooth communication and continued automation • Avoid high IOB at start of water-based activities • Consider insulin delivery suspension with or without disconnecting the AID system where possible • If suspending the AID system, consider reconnecting the pump every hour for small amount of insulin delivery • Note increased risk of hypoglycaemia with water-based sports; have fast-acting glucose readily available
Contact sports (e.g. wrestling, rugby, football, jiu-jitsu, water polo, platform diving)	• Stress response • Devices falling off or being knocked off • Lack of comfort	• Consider location of device wear for increased comfort • Consider insulin delivery suspension with or without disconnecting the AID system where possible if concerned about damaging the pump • Use overlay patches and tape to secure the CGM and AID system in place • If disconnected from the AID system, to prevent insulin deficiency during PA, consider reconnecting the pump and delivering ~50% of the ‘usual basal’ dose every hour • May want to avoid setting higher glucose target 1–2 h before PA if hyperglycaemia occurs with contact sports
High ambient temperature (e.g. hiking in the heat)	• Increased perspiration • Increased insulin absorption • Increased energy expenditure • Increased risk of hypoglycaemia • Possible impact on CGM accuracy	• Consider fingerstick blood glucose monitoring in cases where CGM accuracy may be impacted • Set higher glucose target 1–2 h before exercise until end of activity • Reduce mealtime or snack bolus insulin dose before and during activity (e.g. 25–33% reduction) • Use overlay patches and tape to secure the CGM and AID system in place
Low ambient temperature (e.g. skiing, cold exposure)	• Constriction of peripheral blood vessels, minimal sweat production and increased metabolic heat production (e.g. shivering) • May be less risk for hypoglycaemia based on stress response to cold, but likely depends on duration and intensity of activity	• Concerns over glucose gel and/or insulin in pump freezing due to temperature so keep these items close to the body • Consider carrying hypoglycaemia treatment that cannot freeze (e.g. glucose tablets) • Loss of signal is possible with glucose meter and/or CGM so may need to warm up devices prior to testing • Monitor CGM closely and consider setting higher alert thresholds on the CGM device where possible
High-altitude environments (e.g. skiing, trekking, snowboarding)	• Hypoxia may impact decision-making • Impact on counterregulatory hormones • Increased dysglycaemia from hypoxia • Possible impact on CGM accuracy	• Blood glucose meters may be inaccurate at high altitude; therefore, CGM could be recommended for combined use • Stay hydrated • Lack of evidence on strategies for using AID systems at high altitude • Short-term exposure to high altitude might induce insulin resistance through sympathetic stimulation, but insulin resistance will decrease after several days of exposure • Above 5000 m, more cortisol will be produced and this may increase the risk of hyperglycaemia

Under some circumstances (e.g. prolonged periods of time in the water with pump suspension that can increase the risk of diabetic ketoacidosis), people may choose to switch to multiple daily injection (MDI) therapy for a certain period of time. However, this should be carried out only under the guidance of healthcare professionals and the care team

## Conclusion

In this joint EASD/ISPAD position statement, we provide both general strategies and AID device-specific recommendations to help healthcare professionals and people with type 1 diabetes use these emerging technologies more effectively for planned and unplanned PA. We stress that these recommendations should serve as a starting point for PA and that individual responses to activity should be learned and discussed with the healthcare professional team. Strategies often require fine-tuning and individuals should be prepared for unpredictable glucose responses to PA, even after these strategies have been implemented. While there is individual variation in glycaemic responses to the diverse types of activities that individuals with type 1 diabetes perform, we hope that these evidence-informed recommendations can help individuals optimise glucose self-management in various PA settings.

## Supplementary Information

Below is the link to the electronic supplementary material.

ESM (PDF 4.54 MB)

Slideset of figures (PPTX 5.31 MB)

## Data Availability

All data used within this position statement are included in the manuscript.

## Abbreviations

AID: Automated insulin delivery
CGM: Continuous glucose monitoring
CHO: Carbohydrates
IOB: Insulin on board
ISPAD: International Society for Pediatric and Adolescent Diabetes
PA: Physical activity and exercise
TAR>10.0: Time above range (>10.0 mmol/l)
TBR<3.9: Time below range (<3.9 mmol/l)
TBR<3.0: Time below range (<3.0 mmol/l)
TIR3.9–10.0: Time in range (3.9–10.0 mmol/l)
