# Supplementary material for: The use of automated insulin delivery around physical activity and exercise in type 1 diabetes: a position statement of the European Association for the Study of Diabetes (EASD) and the International Society for Pediatric and Adolescent Diabetes (ISPAD)
Source: Diabetologia. 2024 Dec 10;68(2):255–80. doi: 10.1007/s00125-024-06308-z (PMC11732933; doi:10.1007/s00125-024-06308-z)
Supplement: Supplementary file 1 — ESM (PDF 4.54 MB) [file 125_2024_6308_MOESM1_ESM.pdf]

ESM 1: The Use of Automated Insulin Delivery Around Physical Activity and Exercise in Type 1 Diabetes: A Position Statement of the European Association for the Study of Diabetes (EASD) and the International Society for Pediatric and Adolescent Diabetes (ISPAD)

Emerging Automated Insulin Delivery Technology for Type 1 Diabetes

Tidepool Loop

Tidepool Loop with the Sequel twiist pump (Sequel Med Tech, Manchester, NH) has received FDA 510(k) clearance as an alternate controller enabled (ACE) infusion pump. They have announced partnership with Tidepool to bring the FDA-cleared Loop algorithm to market in the United States for people ≥6 years. The Tidepool Loop algorithm is the commercial version of the previously Open-Source Automated Insulin Delivery (OS-AID) Loop algorithm. It was studied in a prospective real-world observational study in 558 adults and children with type 1 diabetes who initiated Loop either on their own or with community-developed resources [1]. The Tidepool Loop algorithm is a hybrid closed-loop controller using model prediction that anticipates future glucose based on the effects of delivered insulin, user-entered CHO, and two methods of short-term adaptation called “glucose momentum” and “retrospective correction.” The CHO effects are derived from the insulin-to-carbohydrate (I:C) ratio and the insulin sensitivity factor (ISF), and the insulin effect is derived solely from the ISF. The algorithm then modulates basal insulin delivery to drive the sensor glucose value to a user specified target glucose. The algorithm also accounts for user-specified CHO absorption times. Users may set basal rates, I:C, ISF, target glucose, and insulin pharmacodynamic models. To date, no overt exercise studies have been performed on users of the Tidepool Loop algorithm and studies have not yet begun on the commercial build with the Sequel twiist pump.

Data Sources, Searches, and Study Selection

Systematic reviews and meta-analyses were included as additional information for the use of AID technology. Reference lists from each relevant publication were screened to identify additional articles pertinent to the topic. Papers were grouped per theme by device and the lead authors reviewed the evidence. In this position statement, we discuss each commercially available AID system separately (listed in alphabetical order by company), and provide specific recommendations for each system, where possible. Although the recommendations presented in this position statement are based on research evidence, they are the opinions of the authors.

The level of evidence was set according to the Memorandum of Understanding (MOU) between EASD and the writing group of this position statement. Level of evidence is thus expressed as: Ia) evidence from meta-analysis of randomized controlled trials, Ib) evidence from at least one randomized controlled trial, IIa) evidence from at least one controlled study without randomization, IIb) evidence from at least one other type of quasi-experimental study, III) evidence from non-experimental descriptive studies, such as comparative studies, correlation studies, and case-control studies, IV) evidence from expert committee reports or opinions, or both. If recommendations are given within this position statement, the strength of those recommendations are expressed as: (A) directly based on category I evidence; (B) directly based on category II evidence or extrapolated recommendation from category I evidence; (C) directly based on category III evidence or extrapolated recommendation from category I or II evidence; (D) directly based on category IV evidence or extrapolated recommendation from category I, II, or III evidence date for scheduled review or expiry of the guidelines is given; (consensus D) clinical experience of respected authorities.

General Principles of AID, Physical Activity, and Exercise

Insulin on board (IOB) refers to the insulin that is still active in the body from a previous bolus dose [2]. In general, IOB is estimated based on the amount and type of insulin used, and the duration of insulin action; however, there are inconsistencies in how IOB is calculated and displayed on insulin pumps [3]. Moreover, some AID systems allow users to set the duration of insulin action (e.g., between 2-8 hours), which also influences how IOB is reported as well as the aggressiveness of insulin delivery [4]. A shorter duration of IOB (e.g., ≤3 hours) makes the AID algorithm more aggressive and is generally associated with higher TIR3.9-10.0 [5], but likely underrepresents “true” IOB based on the known duration of insulin action for rapid-acting insulin analogues [6]. Conversely, a longer duration of IOB (e.g., >3 hours) generally provides a more accurate IOB display but is associated with lower TIR3.9-10.0. If there is IOB present at the onset of activity, there is a heightened risk of hypoglycaemia during activity, with higher IOB levels predicting greater hypoglycaemia risk [7–9]. Several strategies proposed in this position statement focus on reducing IOB at the onset of exercise **(Table 1)**.

In addition, most AID systems have the option to temporarily set a higher glucose target value (or range) before PA&E, sometimes called the *exercise mode*, *activity mode*, *activity feature*, *physical activity mode*, or *temporary target*, but for simplicity, we use “higher glucose target” for this position statement (more details by each system provided below). Unless a specific AID system is being discussed, for consistency across all AID systems, the general term “higher glucose target” will be used throughout this joint EASD ISPAD position statement. In this position statement, when referring to standardized CGM metrics including glucose time in range (TIR; 3.9-10.0 mmol/L), time below range (TBR level 1 and 2; <3.9 and <3.0 mmol/L), and time above range (TAR level 1 and 2; >10.0 and >13.9 mmol/L), the following abbreviations are used throughout, unless otherwise specified: TIR3.9-10.0, TBR<3.9, TBR<3.0, TAR>10.0, and TAR>13.9.

The suggested CHO amounts above include overlapping ranges since these provide general guidance that lack precision based on several factors such as blood glucose concentrations, IOB, bodyweight, etc. We recommend using absolute amounts of CHO to prevent hypoglycaemia, as this is generally an easier concept to follow during activity. However, based on previous consensus reports, bodyweight-relativized CHO treatments can be useful, especially for children and adolescents during PA&E (~0.2–0.5 g CHO/kg bodyweight) [10, 11]. Furthermore, the amount of CHO recommended to treat hypoglycaemia during PA&E may be lower with AID systems as compared to CSII and MDI therapy [12] **(D).**

Other Considerations for Physical Activity and Exercise

Current consensus guidelines on PA&E self-management may not be ideal for females with type 1 diabetes, as much of the research to date has been conducted on males [13]. However, some recent studies have focused on incorporating more data from females exclusively [14] and a more equal sex distribution [15]. There may be gender-related differences in PA&E preferences, reasons for being physically active, and strategies used to mitigate dysglycaemia with activity [16]. Moreover, glycaemia in individuals with type 1 diabetes is known to be affected by the menstrual cycle [17, 18], which may have further implications on glucose levels during and after activity [19]. For example, glycaemia and insulin needs may be higher in the late luteal phase (last week before menses) relative to the follicular phase [19]. This relative hyperinsulinaemia could set the stage for a greater drop in glycaemia during exercise in the late luteal phase when glucose transport is also independent of insulin [19]. Similarly, it has been observed that insulin needs tend to decrease during the menopause transition [20], yet how these changes affect the required glucose targets for safe exercise has yet to be determined [21]. More research is needed

to understand the influence of sex and menstrual phase on glycaemia before, during, and after PA&E.

Another consideration is that many forms of PA&E may include a competitive element. For some individuals engaging in group or individual competitive events, managing glycaemia is much more difficult, regardless of insulin modality. Perhaps because of stress hormones or other factors, a fear of dysglycaemia may also impact performance [22]. The challenge can be further amplified by the need for CHO to fuel for optimizing PA&E performance. In one large paediatric study, competitive events were associated with higher glucose levels (or less of a drop in glucose during an activity) compared to non-competitive events [23].

A major challenge with AID systems is the inconsistency in insulin levels before and during PA&E due to automated algorithmic adjustments. Users might follow the same pre-exercise routine but start their exercise with varying insulin levels, leading to differing glucose responses. In contrast, with MDI or OL pump therapy, IOB remains relatively consistent, enabling a somewhat more predictable glycaemic response, making it easier to learn and follow a successful plan. In some instances, experienced exercisers with proven routines may choose to switch to manual mode before PA&E, then reactivate the AID algorithm post-exercise to optimize glycaemic outcomes. When considering this approach, it is important to adjust the manual basal rate accordingly and ensure that basal rates are accurate, especially for youth with type 1 diabetes. In addition, other adjunctive agents such as SGLT2 inhibitors and GLP-1 receptor analogues tend to lower insulin requirements in type 1 diabetes but may increase the risk of diabetic ketoacidosis (DKA) [24], however, their impact with PA&E is currently unknown.

Additional figures

Exercise intensity and mode	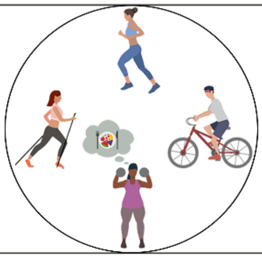		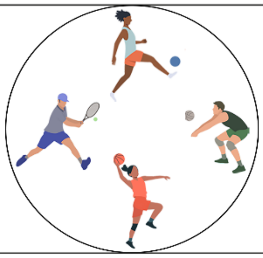	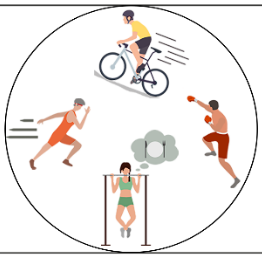	
	Postprandial, continuous, sustained, prolonged, aerobic exercise; low stress hormone response		Mixed activity; individual and team sports	Overnight fasted, burst, explosive, competitive, anaerobic exercise; high stress hormone response	
Average glucose response to exercise	↓	↘	→	↗	↑
Exogenous insulin requirements around exercise	↓	↘	→	↗	↑
Carbohydrate intake requirements around exercise	↑	↗	→	↘	↓

ESM Fig. 1: This figure provides a general overview of glucose trends and exogenous insulin and carbohydrate intake requirements in response to PA in people with type 1 diabetes and does not completely reflect the variability that may exist within and between each individual and in different PA types. The average glucose responses to exercise (top row) are highly variable based on several factors including insulin on board, baseline glucose, glucose rate of change, time of day, fitness level, prandial state, fasted state and menstrual cycle phase. People with type 1 diabetes should understand their individual responses to different types of activity and in different settings (e.g. morning vs afternoon, practice vs competition). Strategies can then be individualised based on their average glucose responses. No one PA can be associated with one glucose trend; however, activities shown in the upper left panel tend to result in the glucose trends in the first two columns; activities shown in the upper right panel tend to result in the glucose trends in the last two columns; and activities shown in the middle panel can result in the glucose trends in the middle three columns. When considering an increase in insulin dose around PA, this should be discussed with the healthcare professional and care team, as only a few studies have investigated higher insulin doses for exercise

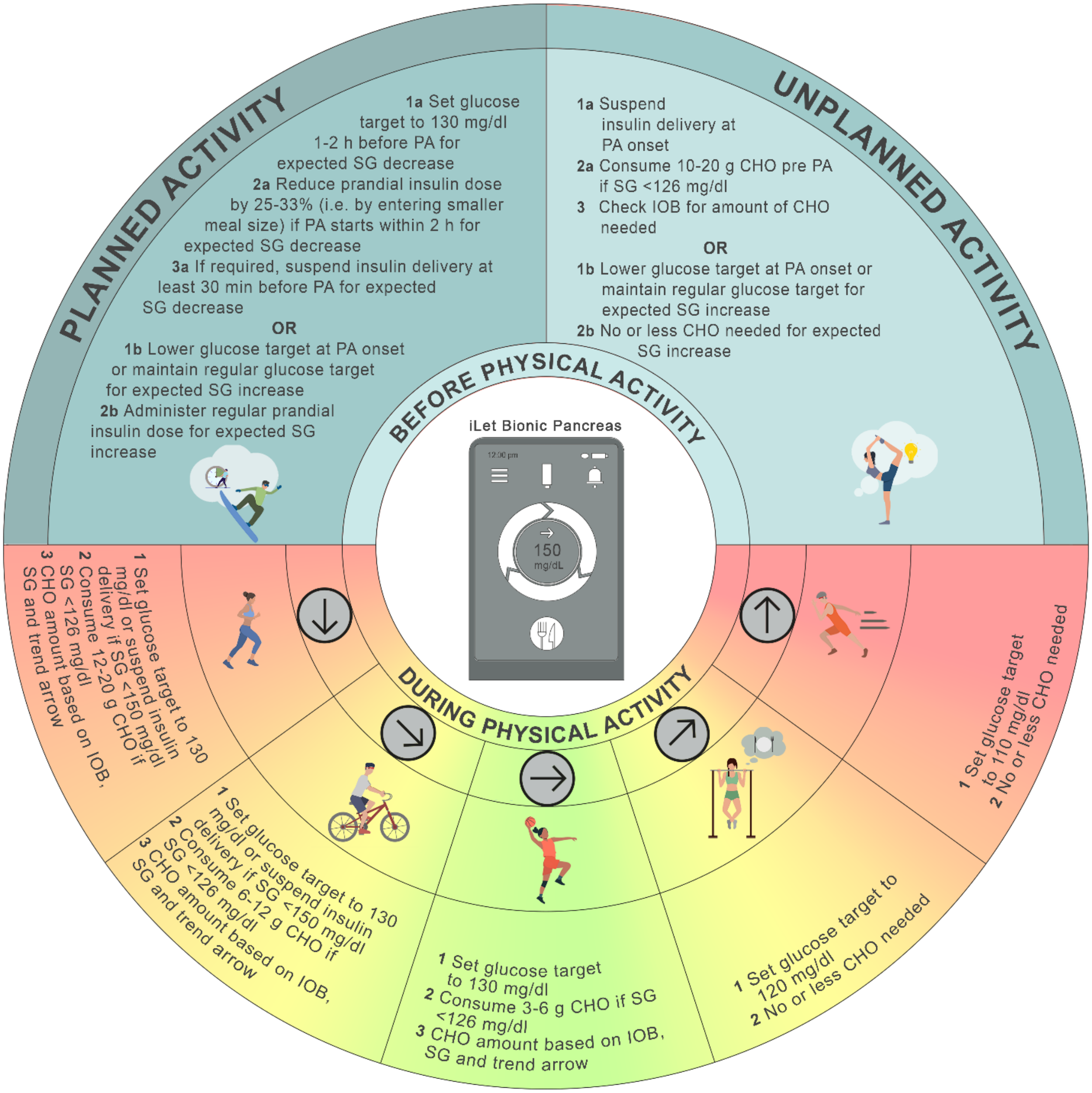

ESM Fig. 2: Recommendations for use of the iLet Bionic Pancreas system to manage glucose outcomes during PA. Consider insulin delivery suspension with or without disconnecting the iLet system 30 min prior to activity to help mitigate hypoglycaemia risk. If ingesting undeclared CHO and disconnecting the iLet before activity, ensure that the device is already suspended and disconnected prior to CHO ingestion. The prandial bolus insulin dose can be reduced only by 'underestimating' CHO (i.e. entering a smaller meal size). SG, sensor glucose. Glucose values: 110 mg/dl = 6.1 mmol/l, 120 mg/dl = 6.7 mmol/l, 126 mg/dl = 7.0 mmol/l, 130 mg/dl = 7.2 mmol/l, 150 mg/dl = 8.3 mmol/l

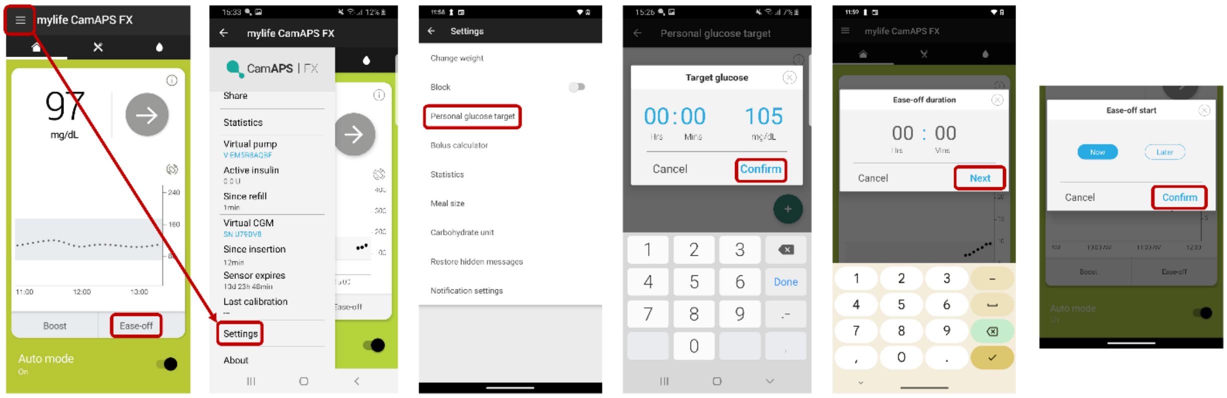

ESM Fig. 3: Illustration of how to set a new personal glucose target and how to set the Ease-off (now or later) mode when using the mylife CamAPS FX system

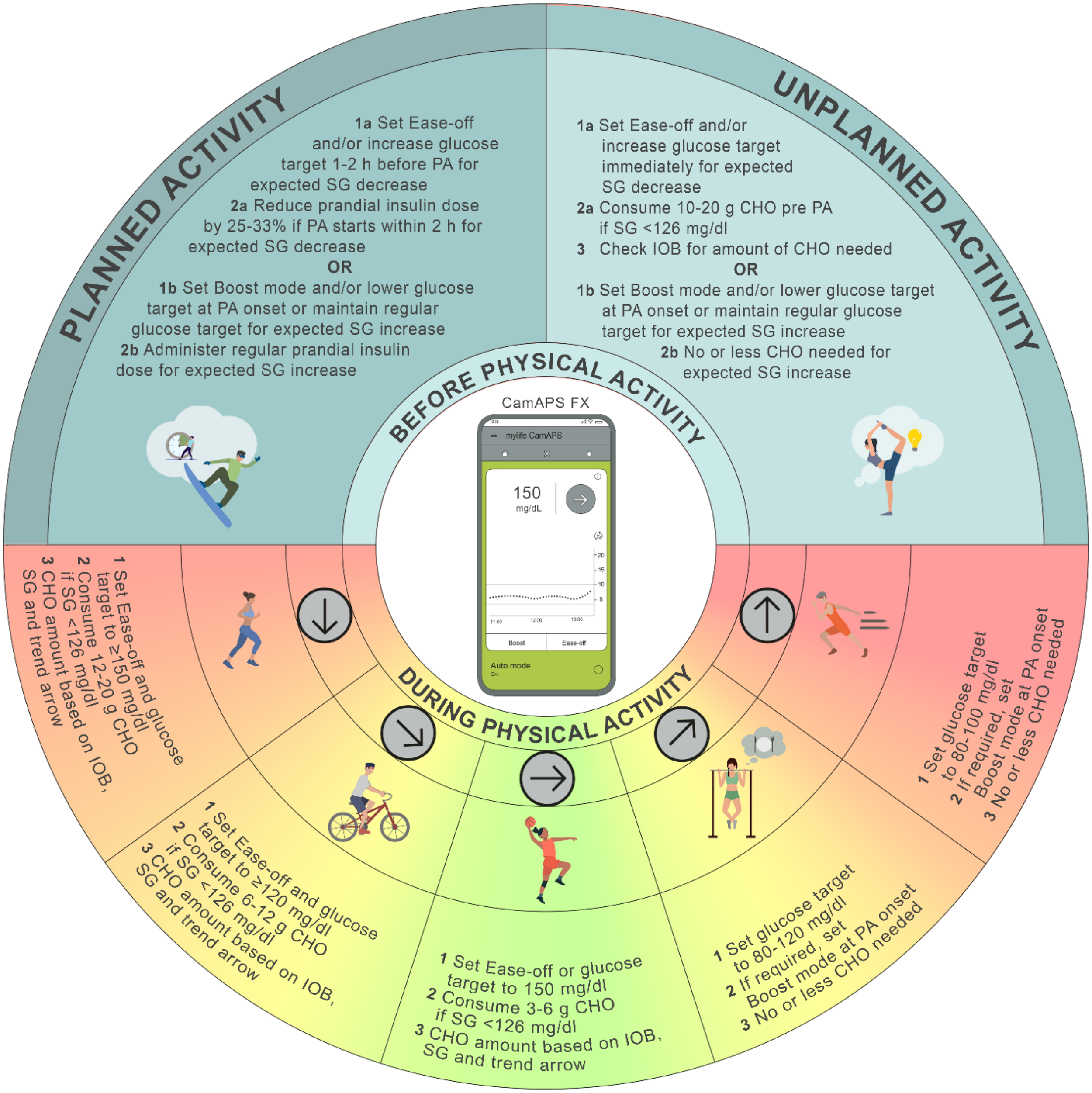

ESM Fig. 4: Recommendations for use of the mylife CamAPS FX system to manage glucose outcomes during PA. Insulin delivery suspension with or without disconnection for prolonged periods (up to 120 min) may be required under some circumstances (e.g. swimming, diving, contact sports), although it is generally not recommended for most activities. SG, sensor glucose. Glucose values: 80 mg/dl = 4.4 mmol/l, 100 mg/dl = 5.6 mmol/l, 120 mg/dl = 6.7 mmol/l, 126 mg/dl = 7.0 mmol/l, 150 mg/dl = 8.3 mmol/l

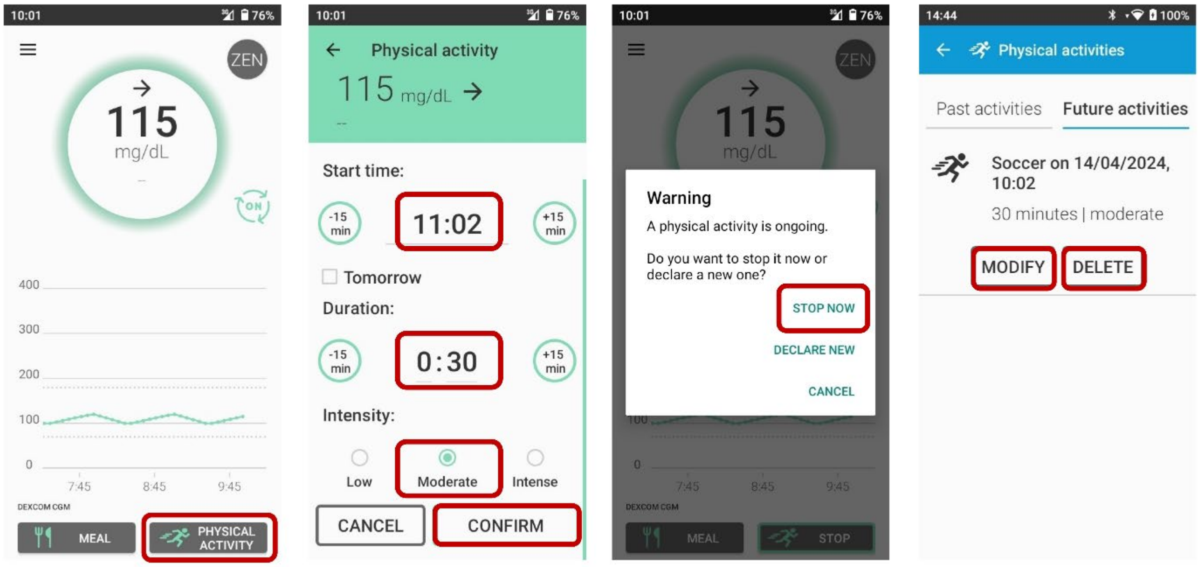

ESM Fig. 5: Illustration of how to start, stop and modify/delete the Physical Activity mode in the DBLG1 system

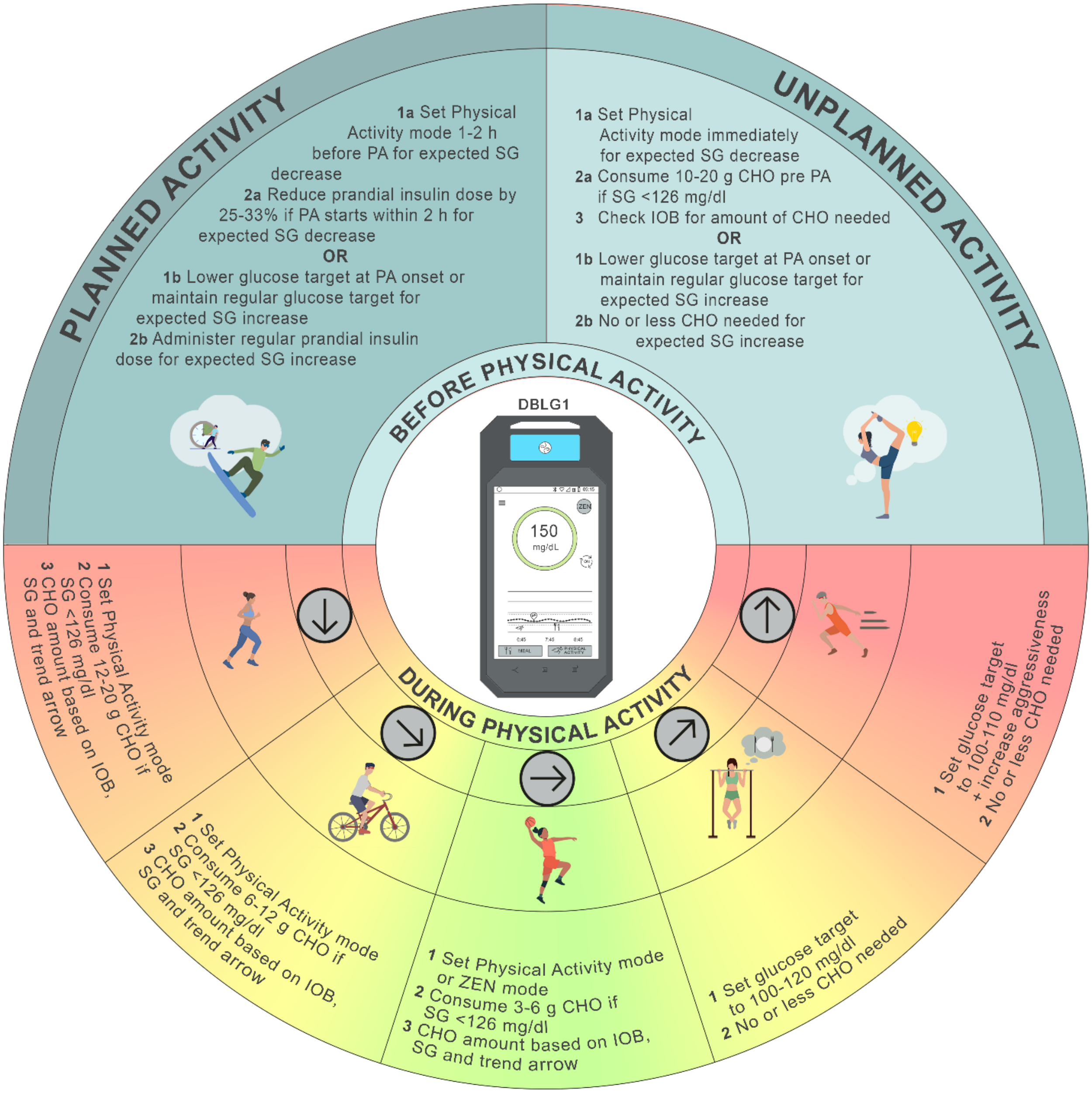

ESM Fig. 6: Recommendations for use of the DBLG1 system to manage glucose outcomes during PA. Insulin delivery suspension with or without disconnection for prolonged periods (up to 120 min) may be required under some circumstances (e.g. swimming, diving, contact sports), although it is generally not recommended for most activities, as several of these strategies cannot be implemented and/or require modification. SG, sensor glucose. Glucose values: 100 mg/dl = 5.6 mmol/l, 110 mg/dl = 6.1 mmol/l, 120 mg/dl = 6.7 mmol/l, 126 mg/dl = 7.0 mmol/l

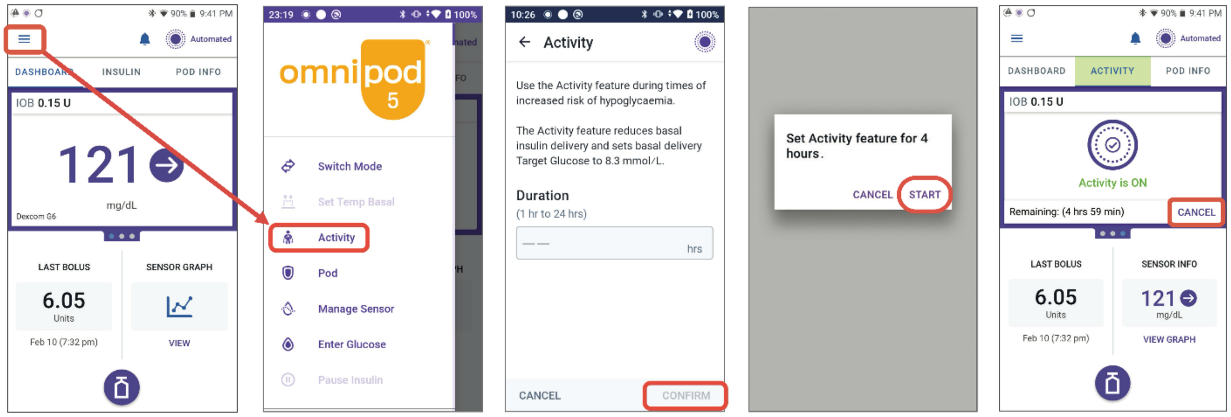

ESM Fig. 7: Illustration of how to set and cancel the Activity feature on the Omnipod 5 system

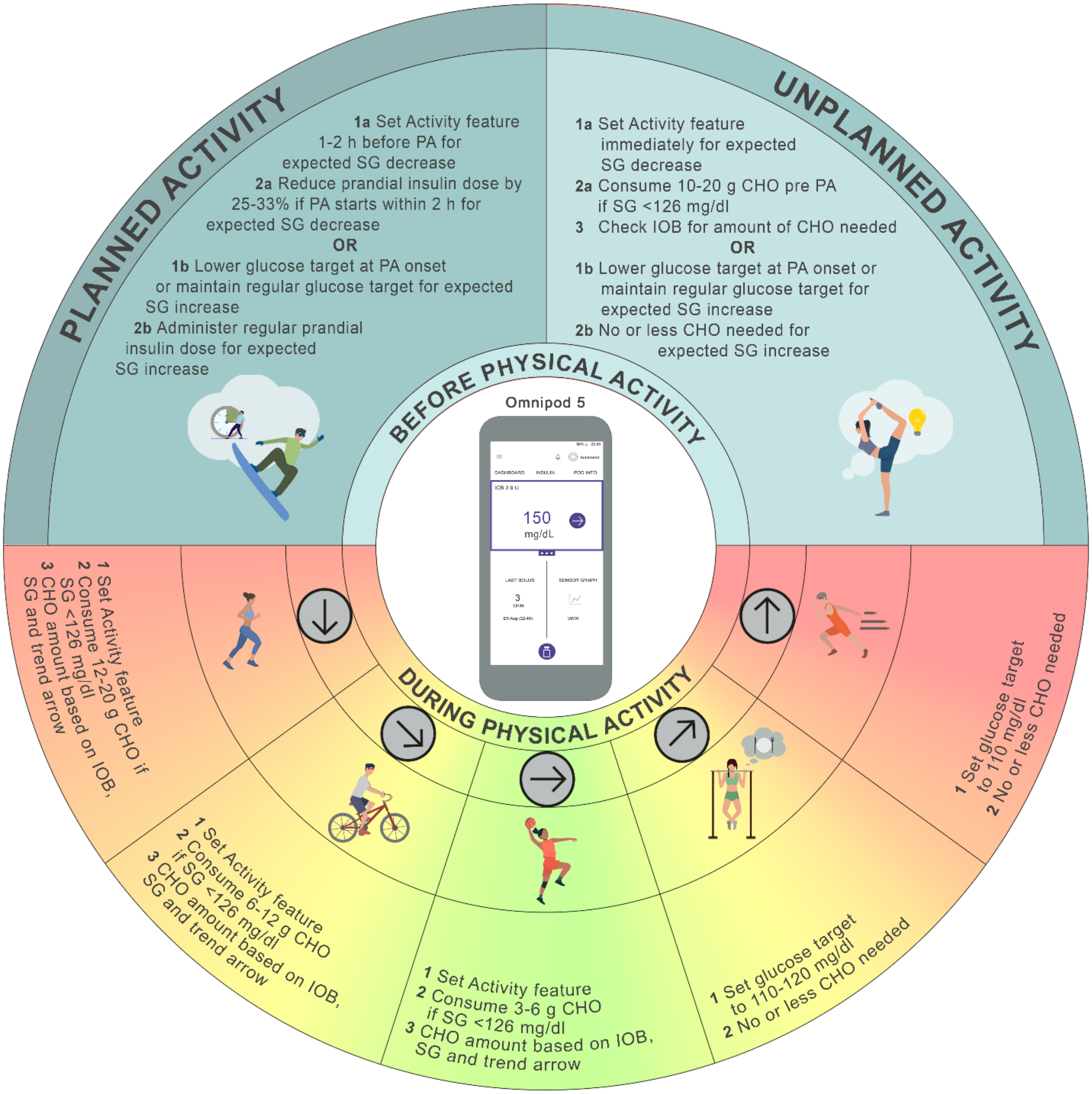

ESM Fig. 8: Recommendations for use of the Omnipod 5 system to manage glucose outcomes during PA. SG, sensor glucose. Glucose values: 110 mg/dl = 6.1 mmol/l, 120 mg/dl = 6.7 mmol/l, 126 mg/dl = 7.0 mmol/l

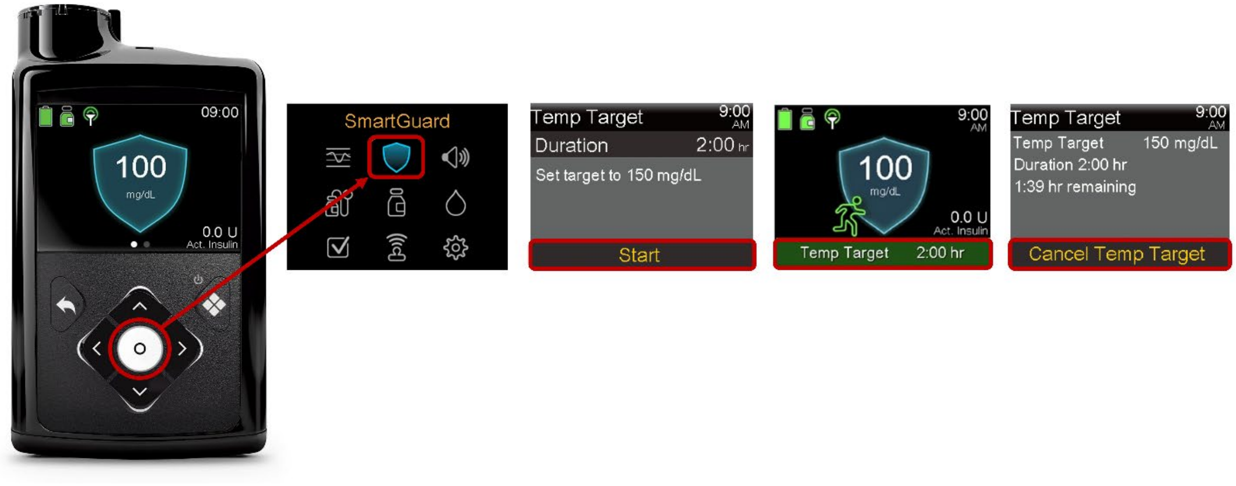

ESM Fig. 9: Illustration of how to set and cancel the Temp Target on the MiniMed 780G system

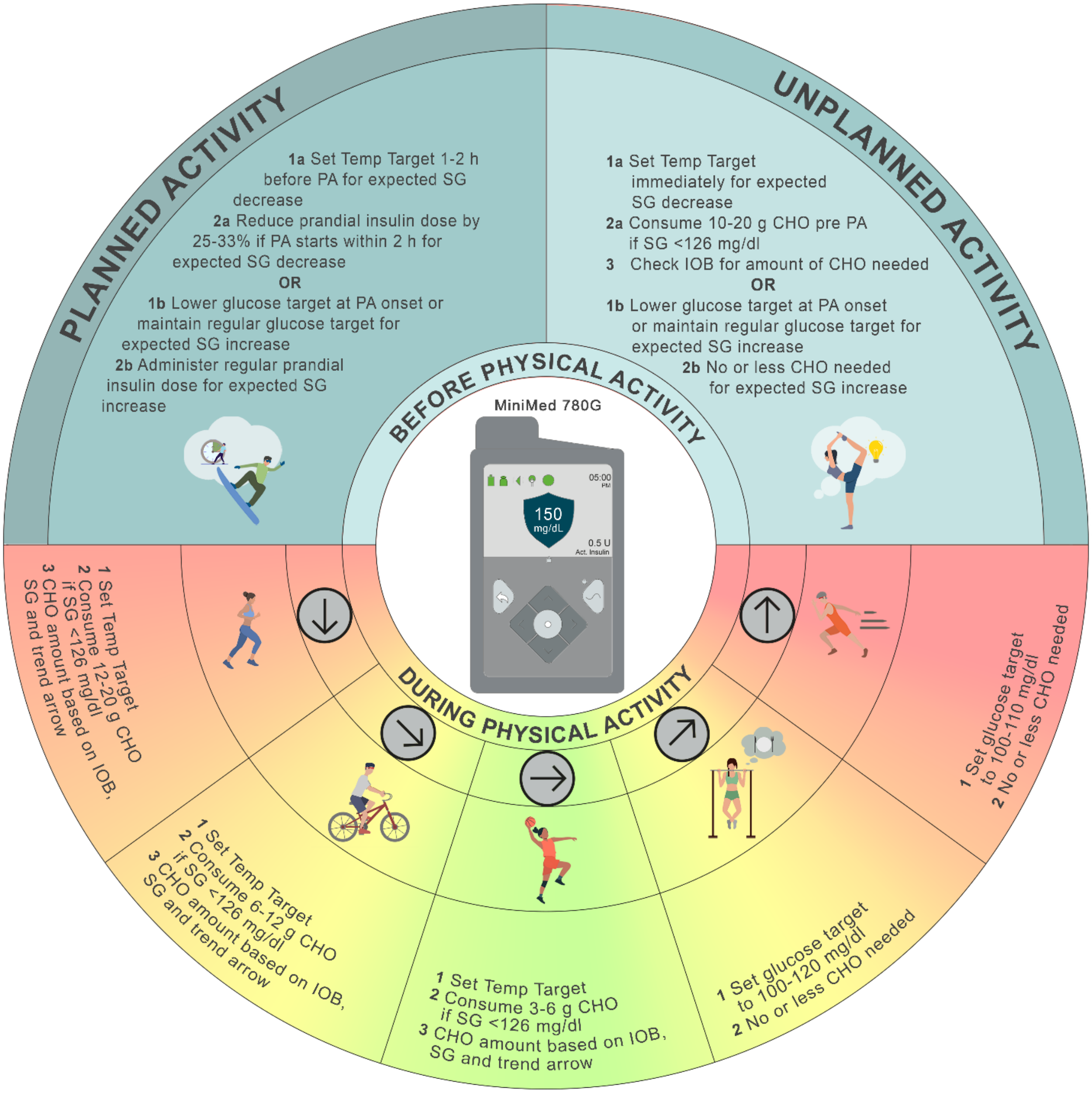

ESM Fig. 10: Recommendations for use of the MiniMed 780G system to manage glucose outcomes during PA. Insulin delivery suspension with or without disconnection for prolonged periods (up to 120 min) may be required under some circumstances (e.g. swimming, diving, contact sports), although it is generally not recommended for most activities, as several of these strategies cannot be implemented and/or require modification. SG, sensor glucose. Glucose values: 100 mg/dl = 5.6 mmol/l, 110 mg/dl = 6.1 mmol/l, 120 mg/dl = 6.7 mmol/l, 126 mg/dl = 7.0 mmol/l

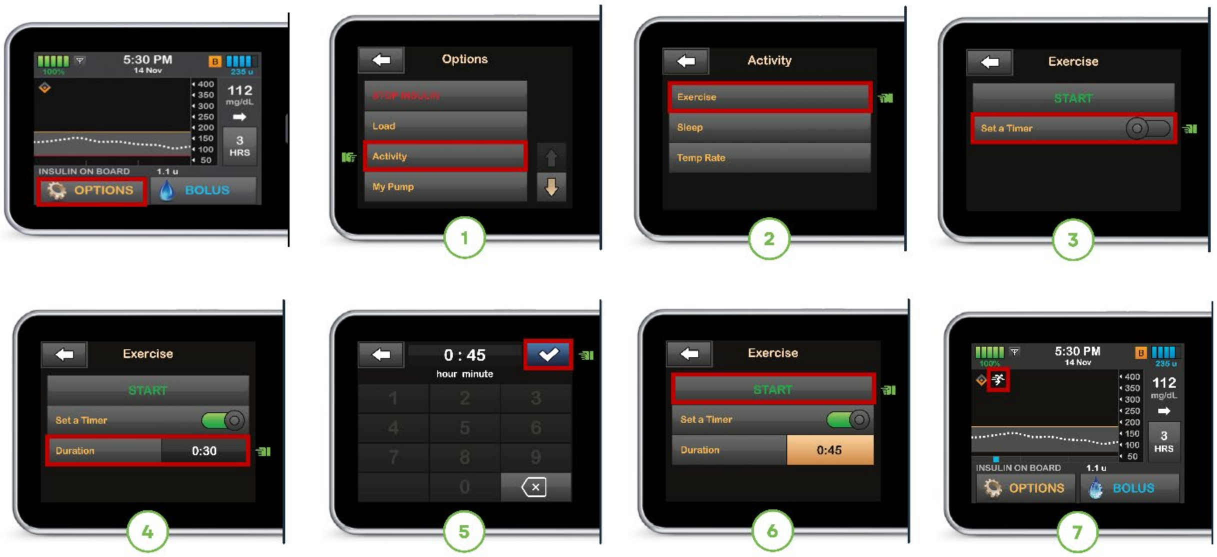

ESM Fig. 11: Illustration of how to set the Exercise mode on the t:slim X2 Control-IQ system

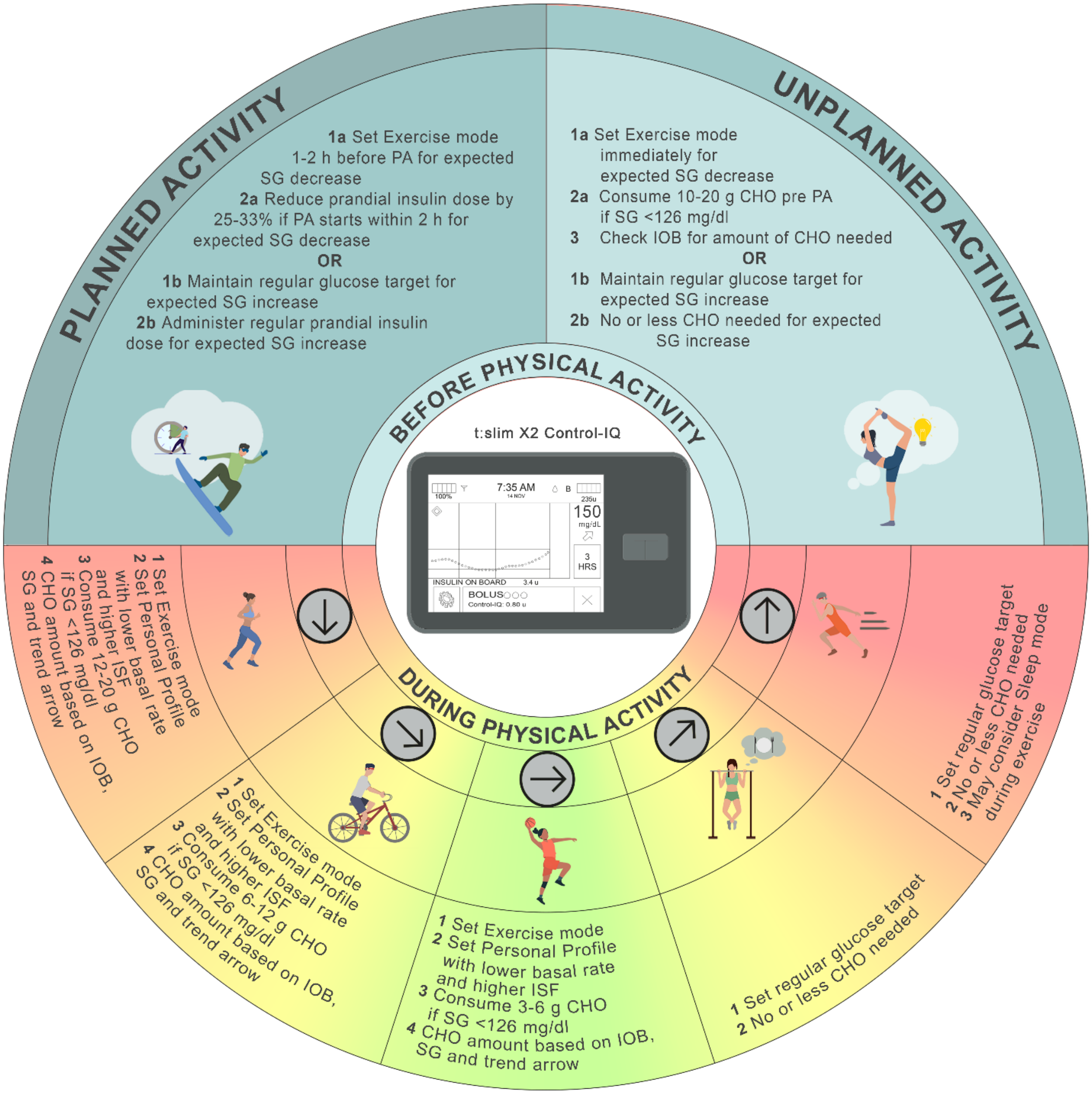

ESM Fig. 12: Recommendations for use of the t:slim X2 Control-IQ system to manage glucose outcomes during PA. Consider adding a minimal manual bolus dose (e.g. 0.05 U) close to the onset of exercise to block the system from administering auto-correction doses for the next 60 min. Insulin delivery suspension with or without disconnection for prolonged periods (up to 120 min) may be required under some circumstances (e.g. swimming, diving, contact sports), although it is generally not recommended for most activities, as several of these strategies cannot be implemented and/or require modification. SG, sensor glucose. Glucose value: 126 mg/dl = 7.0 mmol/l; ISF, insulin sensitivity factor

Reference List for ESM

1. Lum JW, Bailey RJ, Barnes-Lomen V, et al (2021) A Real-World Prospective Study of the Safety and Effectiveness of the Loop Open Source Automated Insulin Delivery System. Diabetes Technol Ther 23(5):367–375. https://doi.org/10.1089/DIA.2020.0535

2. Toffanin C, Zisser H, Doyle FJ, Dassau E (2013) Dynamic insulin on board: incorporation of circadian insulin sensitivity variation. J Diabetes Sci Technol 7(4):928–940. https://doi.org/10.1177/193229681300700415

3. Walsh J, Roberts R, Heinemann L (2014) Confusion Regarding Duration of Insulin Action: A Potential Source for Major Insulin Dose Errors by Bolus Calculators. J Diabetes Sci Technol 8(1):170–178. https://doi.org/10.1177/1932296813514319

4. Choudhary P, Arrieta A, Heuvel T van den, Castañeda J, Smaniotto V, Cohen O (2024) Celebrating the Data from 100,000 Real-World Users of the MiniMedTM 780G System in Europe, Middle East, and Africa Collected Over 3 Years: From Data to Clinical Evidence. Diabetes Technol Ther 26(S3):32–37. https://doi.org/10.1089/DIA.2023.0433

5. Carlson AL, Sherr JL, Shulman DI, et al (2022) Safety and Glycemic Outcomes During the MiniMedTM Advanced Hybrid Closed-Loop System Pivotal Trial in Adolescents and Adults with Type 1 Diabetes. Diabetes Technol Ther 24(3):178–189. https://doi.org/10.1089/DIA.2021.0319

6. Wong EY, Kroon L (2021) Ultra-Rapid-Acting Insulins: How Fast Is Really Needed? Clin Diabetes 39(4):415–423. https://doi.org/10.2337/CD20-0119

7. Riddell MC, Li Z, Gal RL, et al (2023) Examining the Acute Glycemic Effects of Different Types of Structured Exercise Sessions in Type 1 Diabetes in a Real-World Setting: The Type 1 Diabetes and Exercise Initiative (T1DEXI). Diabetes Care 46(4):704–713.

https://doi.org/10.2337/DC22-1721

8. Bergford S, Riddell MC, Jacobs PG, et al (2023) The Type 1 Diabetes and EXercise Initiative: Predicting Hypoglycemia Risk During Exercise for Participants with Type 1 Diabetes Using Repeated Measures Random Forest. Diabetes Technol Ther 25(9). https://doi.org/10.1089/DIA.2023.0140

9. Bergford S, Riddell MC, Gal RL, et al (2024) Predicting Hypoglycemia and Hyperglycemia Risk During and After Activity for Adolescents with Type 1 Diabetes. Diabetes Technol Ther. https://doi.org/10.1089/DIA.2024.0061

10. Adolfsson P, Taplin CE, Zaharieva DP, et al (2022) ISPAD Clinical Practice Consensus Guidelines 2022: Exercise in children and adolescents with diabetes. Pediatr Diabetes 23(8):1341–1372. https://doi.org/10.1111/PEDI.13452

11. Moser O, Riddell MC, Eckstein ML, et al (2020) Glucose management for exercise using continuous glucose monitoring (CGM) and intermittently scanned CGM (isCGM) systems in type 1 diabetes: position statement of the European Association for the Study of Diabetes (EASD) and of the International Society f. Diabetologia 63(12):2501–2520. https://doi.org/10.1007/s00125-020-05263-9

12. O’Neal DN, Zaharieva DP, Morrison D, McCarthy O, Nørgaard K (2024) Exercising Safely with the MiniMedTM 780G Automated Insulin Delivery System. Diabetes Technol Ther 26(S3):84–96. https://doi.org/10.1089/dia.2023.0420

13. Yardley JE, Govette A, Laesser CI, et al (2024) Sex influences health: reporting on female characteristics should be mandatory in exercise and physical activity-related diabetes research. Diabetologia 67(1):209–210. https://doi.org/10.1007/S00125-023-06022-2

14. Sarah McGaugh, Dessi Zaharieva, Rubin Pooni, Ninoschka D'Souza, Jane Yardley MR 683-P: Menstrual Cycle and Glycemic Differences during Prolonged, Fasting Exercise in Females with Type 1 Diabetes. Diabetes, 2020; 69 (Suppl 1) 683-P

15. Momeni Z, Yardley J 243-OR: Effect of Menstrual Cycle on Glycemic Responses to Exercise in Female Participants with Type 1 Diabetes. Diabetes Care

16. Prévost MS, Rabasa-Lhoret R, Talbo MK, Yardley JE, Curry EG, Brazeau AS (2022) Gender Differences in Strategies to Prevent Physical Activity-Related Hypoglycemia in Patients With Type 1 Diabetes: A BETTER Study. Diabetes Care 45(3):e51–e53. https://doi.org/10.2337/DC21-1899

17. Tatulashvili S, Baptiste Julla J, Sritharan N, et al (2022) Ambulatory Glucose Profile According to Different Phases of the Menstrual Cycle in Women Living With Type 1 Diabetes. J Clin Endocrinol Metab 107(10):2793–2800. https://doi.org/10.1210/CLINEM/DGAC443

18. Brown SA, Jiang B, McElwee-Malloy M, Wakeman C, Breton MD (2015) Fluctuations of Hyperglycemia and Insulin Sensitivity Are Linked to Menstrual Cycle Phases in Women With T1D. J Diabetes Sci Technol 9(6):1192–1199. https://doi.org/10.1177/1932296815608400

19. Toor S, Yardley JE, Momeni Z (2023) Type 1 Diabetes and the Menstrual Cycle: Where/How Does Exercise Fit in? Int J Environ Res Public Health 20(4). https://doi.org/10.3390/IJERPH20042772

20. Melmer A, Zürrer I, Kopp F, et al (2021) Differences in insulin dosing in women with type 1 diabetes before and after the menopause. Swiss Med Wkly 151(3940). https://doi.org/10.4414/SMW.2021.W30025

21. Momeni Z, Logan JE, Sigal RJ, Yardley JE (2021) Can Resistance Exercise Be a Tool for Healthy Aging in Post-Menopausal Women with Type 1 Diabetes? Int J Environ Res Public Health 18(16). https://doi.org/10.3390/IJERPH18168716

22. Riddell MC, Scott SN, Fournier PA, et al (2020) The competitive athlete with type 1 diabetes. Diabetologia 63:1475–1490

23. Riddell MC, Gal RL, Bergford S, et al (2023) The Acute Effects of Real-World Physical Activity on Glycemia in Adolescents With Type 1 Diabetes: The Type 1 Diabetes Exercise Initiative Pediatric (T1DEXIP) Study. Diabetes Care. https://doi.org/10.2337/DC23-1548

24. Edwards K, Li X, Lingvay I (2023) Clinical and Safety Outcomes With GLP-1 Receptor Agonists and SGLT2 Inhibitors in Type 1 Diabetes: A Real-World Study. J Clin Endocrinol Metab 108(4):920–930. https://doi.org/10.1210/CLINEM/DGAC618

ESM 2: Members of the writing group

Number	Author	Region	Role	Gender	Research/Work
1	Othmar Moser	EU	First Author	M	Physiology (Adults & Paediatrics)
2	Dessi Zaharieva	US	First Author	F	Exercise Physiology (Adults & Paediatrics)
3	Michael C Riddell	CAN	Last Author	M	Exercise Physiology (Adults & Paediatrics)
4	Peter Adolfsson	EU	Coauthor	M	Paediatrician (Diabetes)
5	Martin Tauschmann	EU	Coauthor	M	Paediatrician (Diabetes)
6	Sabine Hofer	EU	Coauthor (ISPAD)	F	Paediatrician (Diabetes)
7	Tadej Battelino	EU	Coauthor	M	Paediatrician (Diabetes)
8	Klemen Dovc	EU	Coauthor	M	Paediatrician (Diabetes)
9	Jennifer Sherr	US	Coauthor	F	Paediatrician (Diabetes)
10	Richard Bracken	UK	Coauthor	M	Exercise Physiology (Adults)
11	Peter Jacobs	US	Coauthor	M	Engineer (AID)
12	Kirsten Nørgaard	EU	Coauthor	F	Diabetologist/Endocrinologist
13	Julia Mader	EU	Coauthor (EASD)	F	Diabetologist/Endocrinologist
14	Harald Sourij	EU	Coauthor	M	Diabetologist/Endocrinologist
15	Nick S Oliver	UK	Coauthor	M	Diabetologist/Endocrinologist
16	Chantal Matthieu	EU	Coauthor (EASD)	F	Diabetologist/Endocrinologist
17	Roman Hovorka	UK	Coauthor	M	Mathematician (AID)
18	Elizabeth Davis	AUS	Coauthor	F	Paediatrician (Diabetes)
19	Gregory Forlenza	US	Coauthor	M	Paediatrician (Diabetes)
20	Pieter Gillard	EU	Coauthor	M	Diabetologist/Endocrinologist
21	Thomas Danne	EU	Coauthor	M	Paediatrician (Diabetes)
22	John Pemberton	UK	Coauthor	M	Nurse Scientist (paediatrics)

23	Bruce Buckingham	US	Coauthor	M	Paediatrician (Diabetes)
24	Jane Yardley	CAN	Coauthor	F	Exercise Physiology (Adults)
25	Remi Rabasa-Lhoret	CAN	Coauthor	M	Diabetologist/Endocrinologist
26	David O'Neal	AUS	Coauthor	M	Diabetologist/Endocrinologist

ESM 3: Systematic search strategy: The Use of Automated Insulin Delivery (AID) Around Physical Activity and Exercise in Type 1 Diabetes: A Position Statement of the European Association for the Study of Diabetes (EASD) and the International Society for Pediatric and Adolescent Diabetes (ISPAD). Date last search was performed: 08.06.2024.

Search	Actions	Details	Query	Results	Time
#46	···		Search: ((((((((((((((((((diabetes mellitus, type 1 [mh])) OR ((type 1 diabetes mellitus [tw]))) OR ((type 1 diabetes [tw]))) OR (("type i" diabetes mellitus [tw]))) OR (("type-i" diabetes mellitus [tw]))) OR (("insulin-dependent" diabet* [tw]))) OR ((Artificial pancreas [mh]))) OR ((Bioartificial Organs [mh] AND (pancreas [tw] OR insulin [tw] OR diabet* [tw])))) OR ((Bionics [mh] AND (pancreas [tw] OR insulin [tw] OR diabet* [tw])))) OR (("synthetic pancreas" [tw] AND (insulin [tw] OR diabet* [tw])))) OR (("artificial endocrine pancreas" [tw] AND (insulin [tw] OR diabet* [tw])))) OR (("artificial endocrine pancreas" [tw] AND (insulin [tw] OR diabet* [tw])))) OR ((artificial beta cell* [tw] OR artificial b cell* [tw] OR artificial b-cell* [tw]))) OR ((closed- loop* [tw] AND (pancreas [tw] OR insulin [tw] OR diabet* [tw])))) OR (("closed loop*" AND (pancreas [tw] OR insulin [tw] OR diabet* [tw])))) OR (("bioartificial pancreas" [tw] OR "bio-artificial pancreas" [tw]))) AND ((((((((insulin pump [tw])) OR ((insulin delivery system* [tw]))) OR ((insulin [tw]))) OR ((Infusion Pumps, Implantable [mh]))) OR ((Insulin Infusion System [mh]))) OR ((continuous subcutaneous insulin infusion [tw]))) OR ((csii [tw])))) AND ((((((((glucose [tw] AND (sensor* [tw] OR sensing*))) OR (("sensed glucose" [tw]))) OR ((CGM [tw]))) OR ((CGMS [tw]))) OR ((RTCGM [tw]))) OR ((RTCGMS [tw]))) OR ((ICGM [tw])))) AND (((((((Exercise [mh])) OR ((Resistance Training [mh]))) OR ((High-Intensity Interval Training [mh]))) OR ((high-intensity training [tw]))) OR ((high intensity training [tw]))) OR ((physical activity [tw]))) Filters: Full text, English, Humans Sort by: Most Recent	135	09:08:32
#45	···		Search: ((((((((((((((((((diabetes mellitus, type 1 [mh])) OR ((type 1 diabetes mellitus [tw]))) OR ((type 1 diabetes [tw]))) OR (("type i" diabetes mellitus [tw]))) OR (("type-i" diabetes mellitus [tw]))) OR (("insulin-dependent" diabet* [tw]))) OR ((Artificial pancreas [mh]))) OR ((Bioartificial Organs [mh] AND (pancreas [tw] OR insulin [tw] OR	155	09:08:25

Search	Actions	Details	Query	Results	Time
			diabet* [tw])))) OR ((Bionics [mh] AND (pancreas [tw] OR insulin [tw] OR diabet* [tw])))) OR (("synthetic pancreas" [tw] AND (insulin [tw] OR diabet* [tw])))) OR (("artificial endocrine pancreas" [tw] AND (insulin [tw] OR diabet* [tw])))) OR (("artificial endocrine pancreas" [tw] AND (insulin [tw] OR diabet* [tw])))) OR ((artificial beta cell* [tw] OR artificial b cell* [tw] OR artificial b-cell* [tw]))) OR ((closed- loop* [tw] AND (pancreas [tw] OR insulin [tw] OR diabet* [tw])))) OR (("closed loop*" AND (pancreas [tw] OR insulin [tw] OR diabet* [tw])))) OR (("bioartificial pancreas" [tw] OR "bio-artificial pancreas" [tw]))) AND (((((((insulin pump [tw])) OR ((insulin delivery system* [tw]))) OR ((insulin [tw]))) OR ((Infusion Pumps, Implantable [mh]))) OR ((Insulin Infusion System [mh]))) OR ((continuous subcutaneous insulin infusion [tw]))) OR ((csii [tw])))) AND (((((((glucose [tw] AND (sensor* [tw] OR sensing*))) OR (("sensed glucose" [tw]))) OR ((CGM [tw]))) OR ((CGMS [tw]))) OR ((RTCGM [tw]))) OR ((RTCGMS [tw]))) OR ((ICGM [tw])))) AND ((((((Exercise [mh])) OR ((Resistance Training [mh]))) OR ((High-Intensity Interval Training [mh]))) OR ((high-intensity training [tw]))) OR ((high intensity training [tw]))) OR ((physical activity [tw]))) Filters: Full text, English Sort by: Most Recent		
#44	...		Search: ((((((((((((((((((diabetes mellitus, type 1 [mh])) OR ((type 1 diabetes mellitus [tw]))) OR ((type 1 diabetes [tw]))) OR (("type i" diabetes mellitus [tw]))) OR (("type-i" diabetes mellitus [tw]))) OR (("insulin-dependent" diabet* [tw]))) OR ((Artificial pancreas [mh]))) OR ((Bioartificial Organs [mh] AND (pancreas [tw] OR insulin [tw] OR diabet* [tw])))) OR ((Bionics [mh] AND (pancreas [tw] OR insulin [tw] OR diabet* [tw])))) OR (("synthetic pancreas" [tw] AND (insulin [tw] OR diabet* [tw])))) OR (("artificial endocrine pancreas" [tw] AND (insulin [tw] OR diabet* [tw])))) OR (("artificial endocrine pancreas" [tw] AND (insulin [tw] OR diabet* [tw])))) OR ((artificial beta cell* [tw] OR artificial b cell* [tw] OR artificial b-cell* [tw]))) OR ((closed- loop* [tw] AND (pancreas [tw] OR insulin [tw] OR diabet* [tw])))) OR (("closed loop*" AND (pancreas [tw] OR insulin [tw] OR diabet* [tw])))) OR (("bioartificial pancreas" [tw] OR "bio-artificial pancreas" [tw]))) AND (((((((insulin pump [tw])) OR ((insulin delivery system* [tw]))) OR ((insulin [tw]))) OR ((Infusion Pumps, Implantable [mh]))) OR ((Insulin Infusion System [mh]))) OR ((continuous subcutaneous insulin infusion [tw]))) OR ((csii [tw])))) AND (((((((glucose [tw] AND (sensor* [tw] OR sensing*))) OR (("sensed glucose" [tw]))) OR ((CGM [tw]))) OR ((CGMS [tw]))) OR ((RTCGM [tw]))) OR ((RTCGMS [tw]))) OR ((ICGM [tw])))) AND ((((((Exercise [mh])) OR ((Resistance Training [mh]))) OR ((High-Intensity Interval Training [mh]))) OR ((high-intensity training [tw]))) OR ((high intensity training [tw]))) OR ((physical activity [tw]))) Filters: Full text Sort by: Most Recent	156	09:08:19
#43	...		Search: ((((((((((((((((((diabetes mellitus, type 1 [mh])) OR ((type 1 diabetes mellitus [tw]))) OR ((type 1 diabetes [tw]))) OR (("type i" diabetes mellitus [tw]))) OR (("type-i" diabetes mellitus [tw]))) OR (("insulin-dependent" diabet* [tw]))) OR ((Artificial pancreas [mh]))) OR ((Bioartificial Organs [mh] AND (pancreas [tw] OR insulin [tw] OR diabet* [tw])))) OR ((Bionics [mh] AND (pancreas [tw] OR insulin [tw] OR diabet* [tw])))) OR (("synthetic pancreas" [tw] AND (insulin [tw] OR diabet* [tw])))) OR (("artificial endocrine pancreas" [tw] AND (insulin [tw] OR diabet* [tw])))) OR (("artificial endocrine pancreas" [tw] AND (insulin [tw] OR diabet* [tw])))) OR ((artificial beta cell* [tw] OR artificial b cell* [tw] OR artificial b-cell* [tw]))) OR ((closed- loop* [tw] AND (pancreas [tw] OR insulin [tw] OR diabet* [tw])))) OR (("closed loop*" AND (pancreas [tw] OR insulin [tw] OR diabet* [tw])))) OR (("bioartificial pancreas" [tw] OR "bio-artificial pancreas" [tw]))) AND (((((((insulin pump [tw])) OR ((insulin delivery system* [tw]))) OR ((insulin [tw]))) OR ((Infusion Pumps, Implantable [mh]))) OR ((Insulin Infusion System [mh]))) OR ((continuous subcutaneous insulin infusion [tw]))) OR ((csii [tw])))) AND (((((((glucose [tw] AND (sensor* [tw] OR sensing*))) OR (("sensed glucose" [tw]))) OR ((CGM [tw]))) OR ((CGMS [tw]))) OR ((RTCGM [tw]))) OR ((RTCGMS [tw]))) OR ((ICGM [tw])))) AND ((((((Exercise [mh])) OR ((Resistance Training [mh]))) OR ((High-Intensity Interval Training [mh]))) OR ((high-	163	09:08:13

Search	Actions	Details	Query	Results	Time
			intensity training [tw]))) OR ((high intensity training [tw]))) OR ((physical activity [tw]))) Sort by: Most Recent		
#42	...		Search: ((((((Exercise [mh])) OR ((Resistance Training [mh]))) OR ((High-Intensity Interval Training [mh]))) OR ((high-intensity training [tw]))) OR ((high intensity training [tw]))) OR ((physical activity [tw])) Sort by: Most Recent	351,370	09:08:01
#41	...		Search: (physical activity [tw]) Sort by: Most Recent	159,685	09:07:39
#40	...		Search: (high intensity training [tw]) Sort by: Most Recent	1,042	09:07:33
#39	...		Search: (high-intensity training [tw]) Sort by: Most Recent	1,042	09:07:23
#38	...		Search: (High-Intensity Interval Training [mh]) Sort by: Most Recent	2,394	09:07:14
#37	...		Search: (Resistance Training [mh]) Sort by: Most Recent	13,023	09:07:05
#36	...		Search: (Exercise [mh]) Sort by: Most Recent	257,440	09:06:59
#35	...		Search: ((((((((((((((((diabetes mellitus, type 1 [mh])) OR ((type 1 diabetes mellitus [tw]))) OR ((type 1 diabetes [tw]))) OR (("type i" diabetes mellitus [tw]))) OR (("type-i" diabetes mellitus [tw]))) OR (("insulin-dependent" diabet* [tw]))) OR ((Artificial pancreas [mh]))) OR ((Bioartificial Organs [mh] AND (pancreas [tw] OR insulin [tw] OR diabet* [tw])))) OR ((Bionics [mh] AND (pancreas [tw] OR insulin [tw] OR diabet* [tw])))) OR (("synthetic pancreas" [tw] AND (insulin [tw] OR diabet* [tw])))) OR (("artificial endocrine pancreas" [tw] AND (insulin [tw] OR diabet* [tw])))) OR (("artificial endocrine pancreas" [tw] AND (insulin [tw] OR diabet* [tw])))) OR ((artificial beta cell* [tw] OR artificial b cell* [tw] OR artificial b-cell* [tw]))) OR ((closed- loop* [tw] AND (pancreas [tw] OR insulin [tw] OR diabet* [tw])))) OR (("closed loop*" AND (pancreas [tw] OR insulin [tw] OR diabet* [tw])))) OR (("bioartificial pancreas" [tw] OR "bio-artificial pancreas" [tw]))) AND ((((((((insulin pump [tw])) OR ((insulin delivery system* [tw]))) OR ((insulin [tw]))) OR ((Infusion Pumps, Implantable [mh]))) OR ((Insulin Infusion System [mh]))) OR ((continuous subcutaneous insulin infusion [tw]))) OR ((csii [tw])))) AND ((((((((glucose [tw] AND (sensor* [tw] OR sensing*))) OR (("sensed glucose" [tw]))) OR ((CGM [tw]))) OR ((CGMS [tw]))) OR ((RTCGM [tw]))) OR ((RTCGMS [tw]))) OR ((ICGM [tw]))) Sort by: Most Recent	2,954	09:06:48
#34	...		Search: (((((((glucose [tw] AND (sensor* [tw] OR sensing*))) OR (("sensed glucose" [tw]))) OR ((CGM [tw]))) OR ((CGMS [tw]))) OR ((RTCGM [tw]))) OR ((RTCGMS [tw]))) OR ((ICGM [tw])) Sort by: Most Recent	23,643	09:06:19
#33	...		Search: (ICGM [tw]) Sort by: Most Recent	27	09:05:41
#32	...		Search: (RTCGMS [tw]) Sort by: Most Recent	25	09:05:35
#31	...		Search: (RTCGM [tw]) Sort by: Most Recent	262	09:05:29
#30	...		Search: (CGMS [tw]) Sort by: Most Recent	1,014	09:05:23
#29	...		Search: (CGM [tw]) Sort by: Most Recent	4,640	09:05:14
#28	...		Search: ("sensed glucose" [tw]) Sort by: Most Recent	8	09:05:06
#27	...		Search: (glucose [tw] AND (sensor* [tw] OR sensing*)) Sort by: Most Recent	19,399	09:04:59
#26	...		Search: ((((((((((((((((diabetes mellitus, type 1 [mh])) OR ((type 1 diabetes mellitus [tw]))) OR ((type 1 diabetes [tw]))) OR (("type i" diabetes mellitus [tw]))) OR (("type-i" diabetes mellitus [tw]))) OR (("insulin-dependent" diabet* [tw]))) OR ((Artificial pancreas [mh]))) OR ((Bioartificial Organs [mh] AND (pancreas [tw] OR insulin [tw] OR diabet* [tw])))) OR ((Bionics [mh] AND (pancreas [tw] OR insulin [tw] OR diabet* [tw])))) OR (("synthetic pancreas" [tw] AND (insulin [tw] OR diabet* [tw])))) OR (("artificial endocrine pancreas" [tw] AND (insulin [tw] OR diabet* [tw])))) OR (("artificial endocrine pancreas" [tw] AND (insulin [tw] OR diabet* [tw])))) OR ((artificial beta cell*	64,844	09:04:47

Search	Actions	Details	Query	Results	Time
			[tw] OR artificial b cell* [tw] OR artificial b-cell* [tw]))) OR ((closed- loop* [tw] AND (pancreas [tw] OR insulin [tw] OR diabet* [tw])))) OR (("closed loop*" AND (pancreas [tw] OR insulin [tw] OR diabet* [tw])))) OR (("bioartificial pancreas" [tw] OR "bio-artificial pancreas" [tw]))) AND (((((((insulin pump [tw])) OR ((insulin delivery system* [tw]))) OR ((insulin [tw]))) OR ((Infusion Pumps, Implantable [mh]))) OR ((Insulin Infusion System [mh]))) OR ((continuous subcutaneous insulin infusion [tw]))) OR ((csii [tw]))) Sort by: Most Recent		
#25	...		Search: (((((((insulin pump [tw])) OR ((insulin delivery system* [tw]))) OR ((insulin [tw]))) OR ((Infusion Pumps, Implantable [mh]))) OR ((Insulin Infusion System [mh]))) OR ((continuous subcutaneous insulin infusion [tw]))) OR ((csii [tw])) Sort by: Most Recent	483,391	09:04:22
#24	...		Search: (csii [tw]) Sort by: Most Recent	1,871	09:03:55
#23	...		Search: (continuous subcutaneous insulin infusion [tw]) Sort by: Most Recent	2,362	09:03:49
#22	...		Search: (Insulin Infusion System [mh]) Sort by: Most Recent	6,630	09:03:42
#21	...		Search: (Infusion Pumps, Implantable [mh]) Sort by: Most Recent	3,860	09:03:31
#20	...		Search: (insulin [tw]) Sort by: Most Recent	479,975	09:03:25
#19	...		Search: (insulin delivery system* [tw]) Sort by: Most Recent	879	09:03:13
#18	...		Search: (insulin pump [tw]) Sort by: Most Recent	3,143	09:03:06
#17	...		Search: (((((((((((((((diabetes mellitus, type 1 [mh])) OR ((type 1 diabetes mellitus [tw]))) OR ((type 1 diabetes [tw]))) OR (("type i" diabetes mellitus [tw]))) OR (("type-i" diabetes mellitus [tw]))) OR (("insulin-dependent" diabet* [tw]))) OR ((Artificial pancreas [mh]))) OR ((Bioartificial Organs [mh] AND (pancreas [tw] OR insulin [tw] OR diabet* [tw])))) OR ((Bionics [mh] AND (pancreas [tw] OR insulin [tw] OR diabet* [tw])))) OR (("synthetic pancreas" [tw] AND (insulin [tw] OR diabet* [tw])))) OR (("artificial endocrine pancreas" [tw] AND (insulin [tw] OR diabet* [tw])))) OR (("artificial endocrine pancreas" [tw] AND (insulin [tw] OR diabet* [tw])))) OR ((artificial beta cell* [tw] OR artificial b cell* [tw] OR artificial b-cell* [tw]))) OR ((closed- loop* [tw] AND (pancreas [tw] OR insulin [tw] OR diabet* [tw])))) OR (("closed loop*" AND (pancreas [tw] OR insulin [tw] OR diabet* [tw])))) OR (("bioartificial pancreas" [tw] OR "bio-artificial pancreas" [tw])) Sort by: Most Recent	124,057	09:02:44
#16	...		Search: ("bioartificial pancreas" [tw] OR "bio-artificial pancreas" [tw]) Sort by: Most Recent	299	09:01:47
#15	...		Search: ("closed loop*" AND (pancreas [tw] OR insulin [tw] OR diabet* [tw])) Sort by: Most Recent	2,105	09:01:42
#14	...		Search: (closed-loop* [tw] AND (pancreas [tw] OR insulin [tw] OR diabet* [tw])) Sort by: Most Recent	2,087	09:01:36
#13	...		Search: (artificial beta cell* [tw] OR artificial b cell* [tw] OR artificial b-cell* [tw]) Sort by: Most Recent	163	09:01:29
#12	...		Search: ("artificial endocrine pancreas" [tw] AND (insulin [tw] OR diabet* [tw])) Sort by: Most Recent	200	09:01:21
#11	...		Search: ("synthetic pancreas" [tw] AND (insulin [tw] OR diabet* [tw])) Sort by: Most Recent	4	09:01:15
#10	...		Search: (Bionics [mh] AND (pancreas [tw] OR insulin [tw] OR diabet* [tw])) Sort by: Most Recent	37	09:01:05
#9	...		Search: (Bioartificial Organs [mh] AND (pancreas [tw] OR insulin [tw] OR diabet* [tw])) Sort by: Most Recent	76	09:00:55
#8	...		Search: (Artificial pancreas [mh]) Sort by: Most Recent	1,040	09:00:46
#7	...		Search: ("insulin-dependent" diabet* [tw]) Sort by: Most Recent	28,634	09:00:38

Search	Actions	Details	Query	Results	Time
#6	...		Search: ("type-i" diabetes mellitus [tw]) Sort by: Most Recent	9,713	09:00:28
#5	...		Search: ("type i" diabetes mellitus [tw]) Sort by: Most Recent	9,713	09:00:18
#4	...		Search: (type 1 diabetes [tw]) Sort by: Most Recent	104,131	09:00:08
#3	...		Search: (type 1 diabetes mellitus [tw]) Sort by: Most Recent	14,466	08:59:52
#2	...		Search: (diabetes mellitus, type 1 [mh]) Sort by: Most Recent	88,707	08:59:39

Showing 1 to 45 of 45 entries
