# Supplementary material for: The use of automated insulin delivery around physical activity and exercise in type 1 diabetes: a position statement of the European Association for the Study of Diabetes (EASD) and the International Society for Pediatric and Adolescent Diabetes (ISPAD)
Source: Diabetologia. 2024 Dec 10;68(2):255–80. doi: 10.1007/s00125-024-06308-z (PMC11732933; doi:10.1007/s00125-024-06308-z)
Supplement: Supplementary file 2 — Slideset of figures (PPTX 5.31 MB) [file 125_2024_6308_MOESM2_ESM.pptx]

## Slide 1
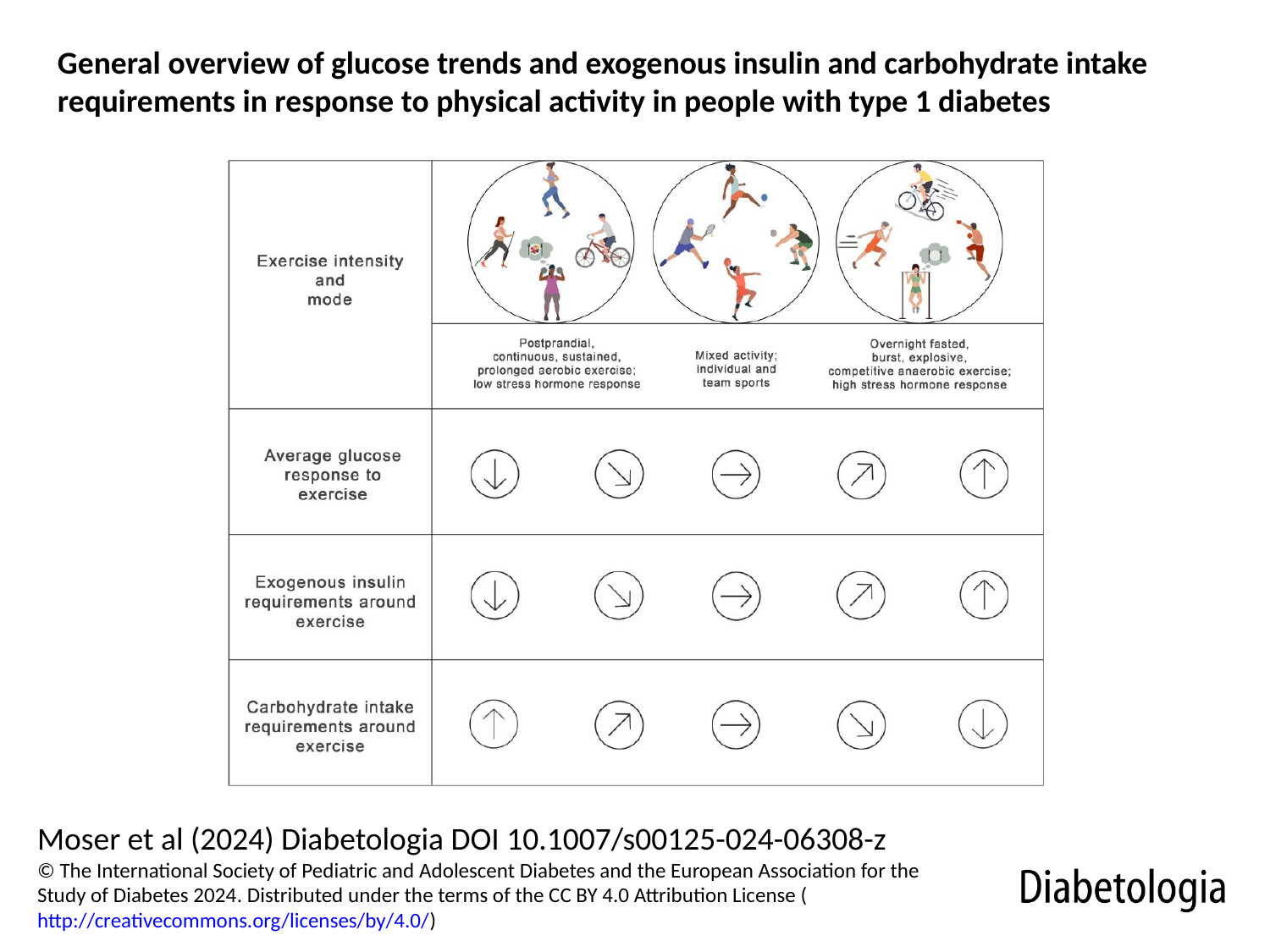

General overview of glucose trends and exogenous insulin and carbohydrate intake requirements in response to physical activity in people with type 1 diabetes
Moser et al (2024) Diabetologia DOI 10.1007/s00125-024-06308-z
© The International Society of Pediatric and Adolescent Diabetes and the European Association for the Study of Diabetes 2024. Distributed under the terms of the CC BY 4.0 Attribution License (http://creativecommons.org/licenses/by/4.0/)

## Slide 2
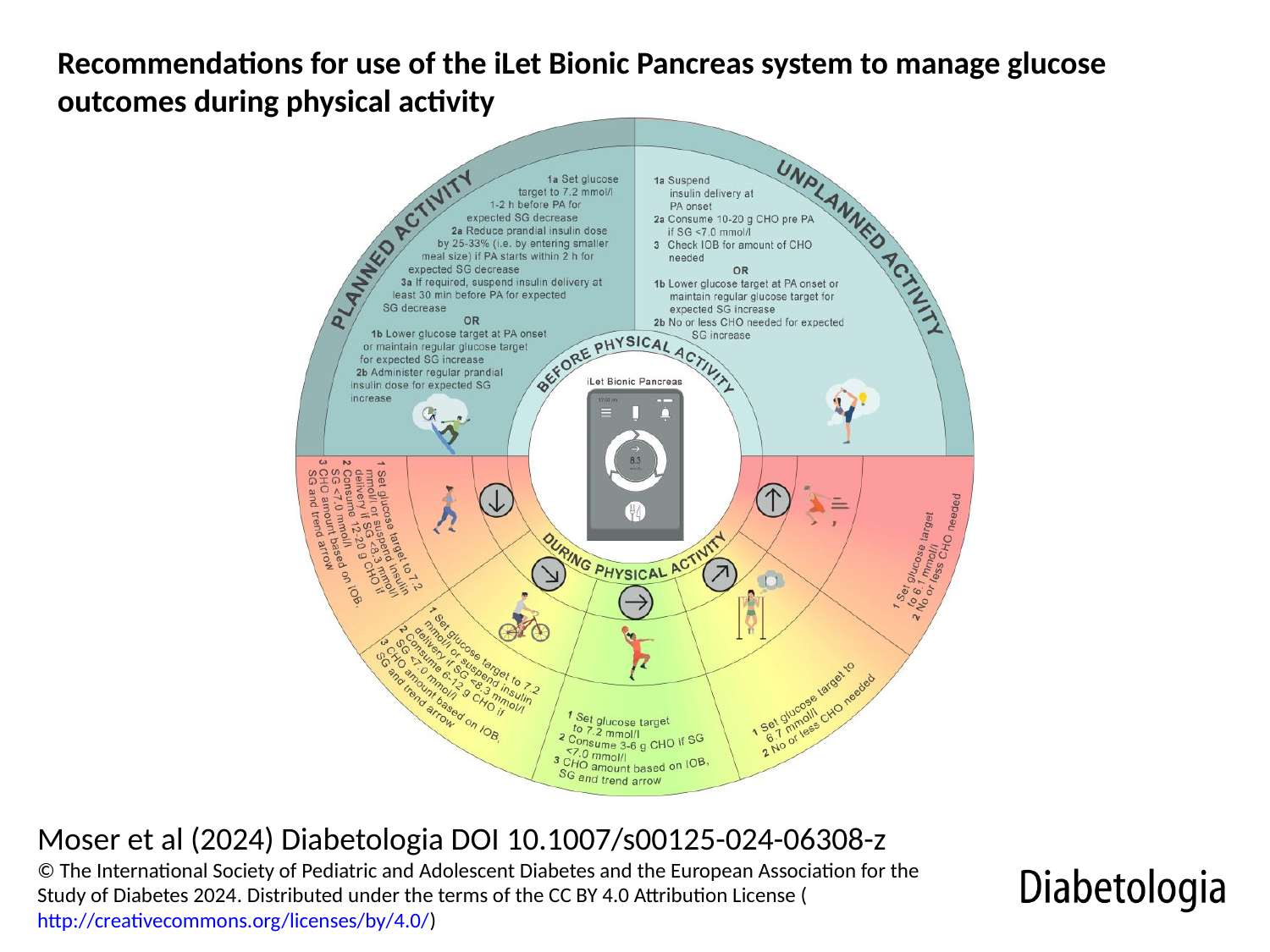

Recommendations for use of the iLet Bionic Pancreas system to manage glucose outcomes during physical activity
Moser et al (2024) Diabetologia DOI 10.1007/s00125-024-06308-z
© The International Society of Pediatric and Adolescent Diabetes and the European Association for the Study of Diabetes 2024. Distributed under the terms of the CC BY 4.0 Attribution License (http://creativecommons.org/licenses/by/4.0/)

## Slide 3
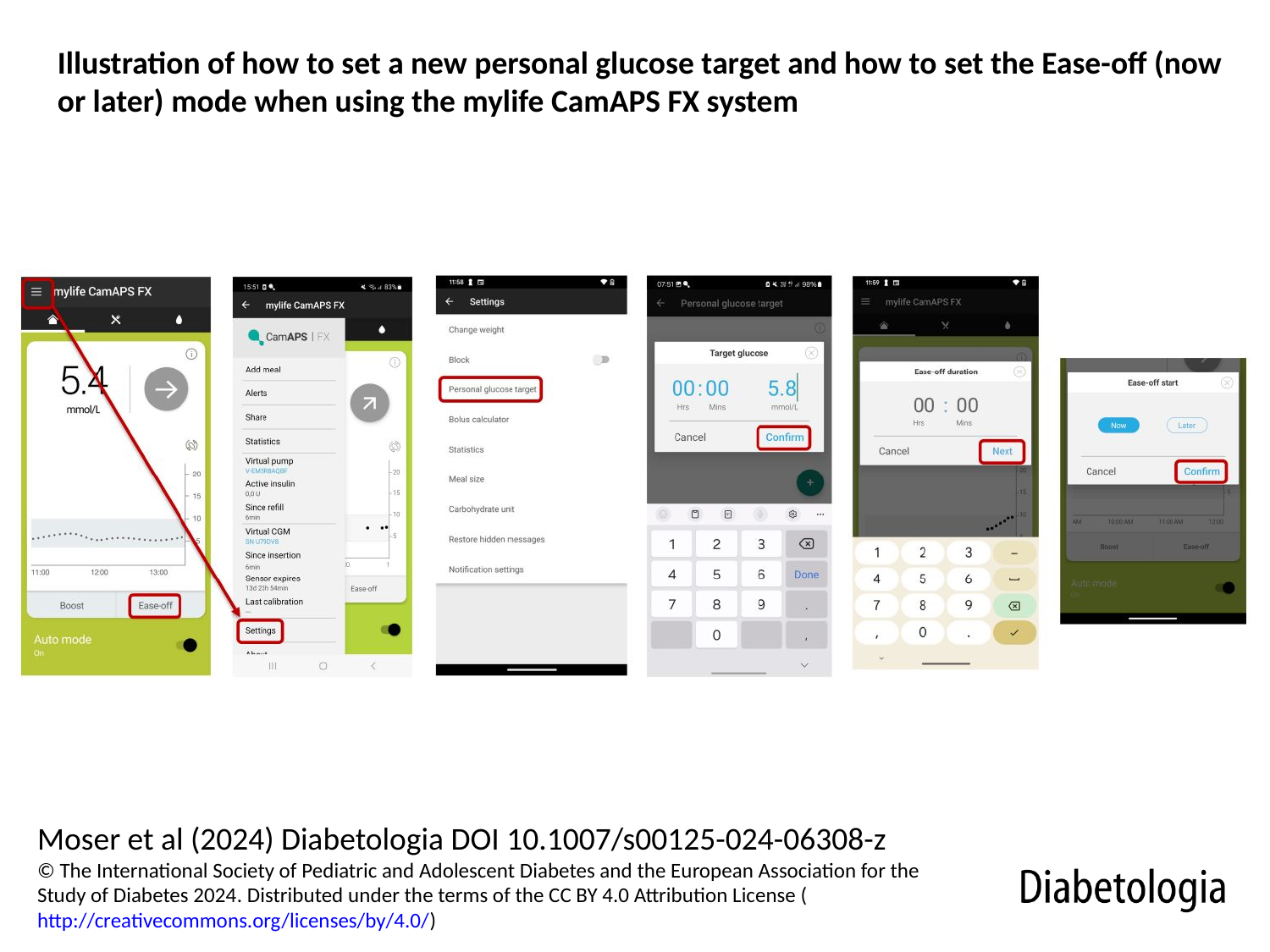

Illustration of how to set a new personal glucose target and how to set the Ease-off (now or later) mode when using the mylife CamAPS FX system
Moser et al (2024) Diabetologia DOI 10.1007/s00125-024-06308-z
© The International Society of Pediatric and Adolescent Diabetes and the European Association for the Study of Diabetes 2024. Distributed under the terms of the CC BY 4.0 Attribution License (http://creativecommons.org/licenses/by/4.0/)

## Slide 4
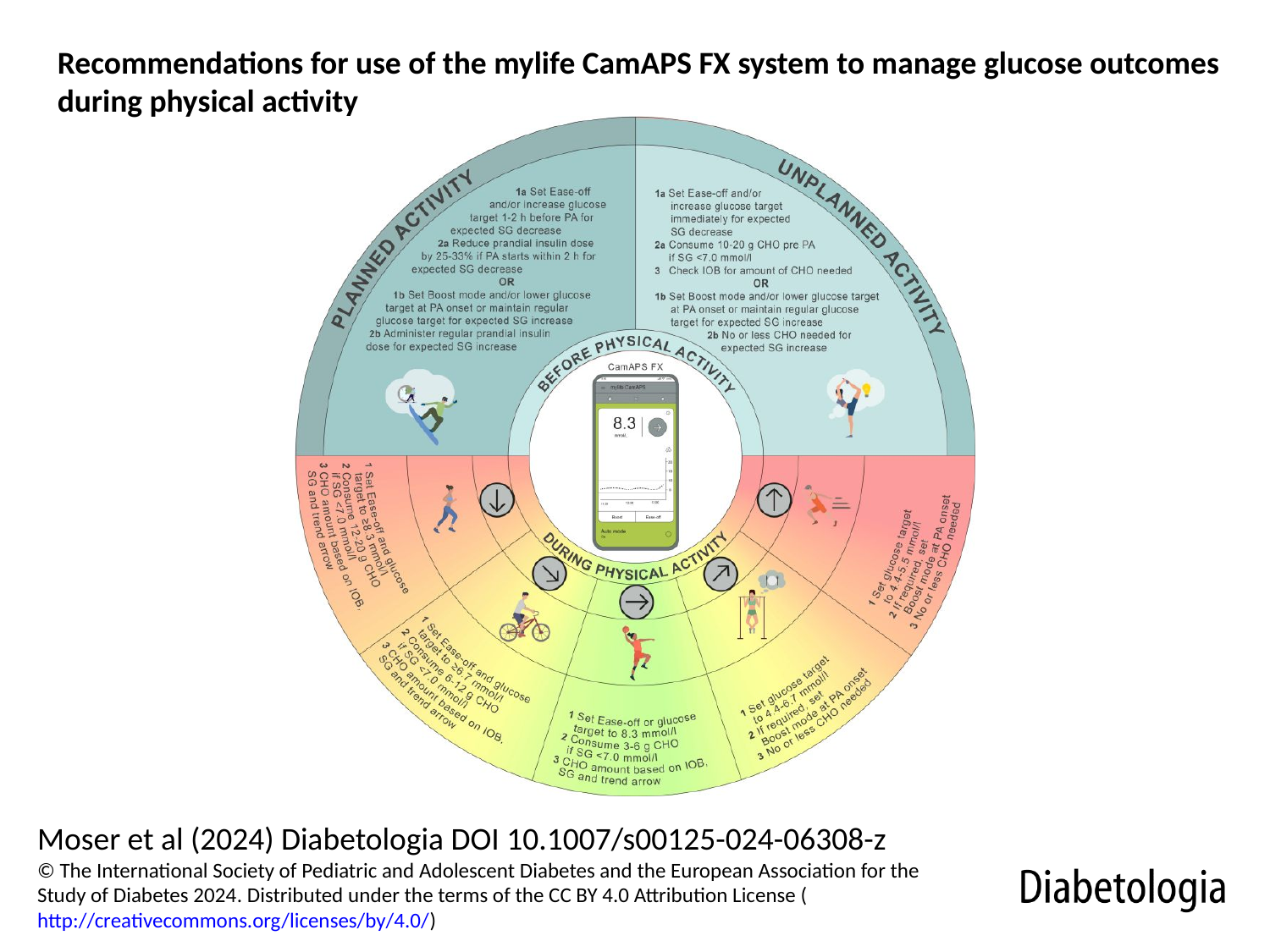

Recommendations for use of the mylife CamAPS FX system to manage glucose outcomes during physical activity
Moser et al (2024) Diabetologia DOI 10.1007/s00125-024-06308-z
© The International Society of Pediatric and Adolescent Diabetes and the European Association for the Study of Diabetes 2024. Distributed under the terms of the CC BY 4.0 Attribution License (http://creativecommons.org/licenses/by/4.0/)

## Slide 5
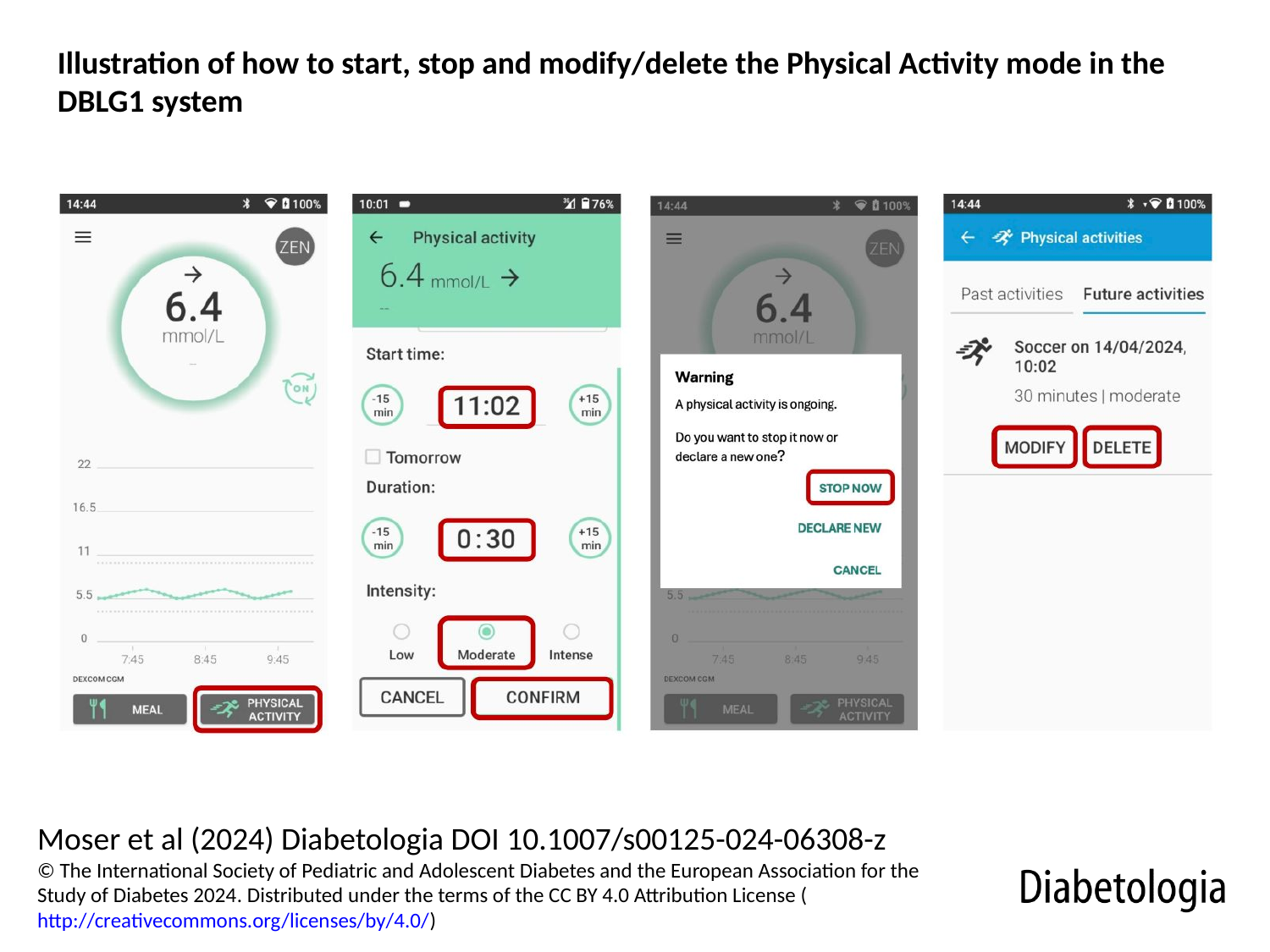

Illustration of how to start, stop and modify/delete the Physical Activity mode in the DBLG1 system
Moser et al (2024) Diabetologia DOI 10.1007/s00125-024-06308-z
© The International Society of Pediatric and Adolescent Diabetes and the European Association for the Study of Diabetes 2024. Distributed under the terms of the CC BY 4.0 Attribution License (http://creativecommons.org/licenses/by/4.0/)

## Slide 6
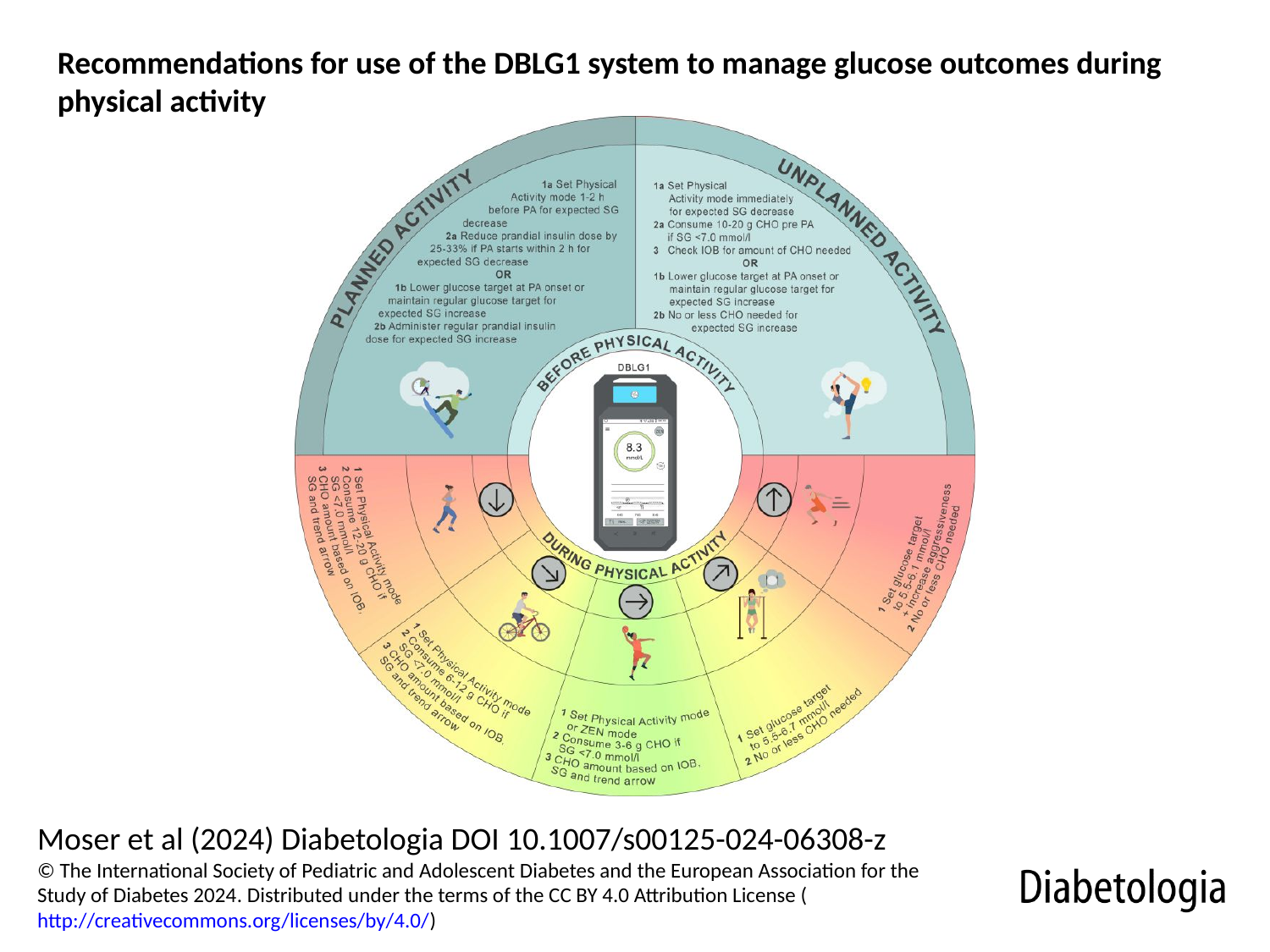

Recommendations for use of the DBLG1 system to manage glucose outcomes during physical activity
Moser et al (2024) Diabetologia DOI 10.1007/s00125-024-06308-z
© The International Society of Pediatric and Adolescent Diabetes and the European Association for the Study of Diabetes 2024. Distributed under the terms of the CC BY 4.0 Attribution License (http://creativecommons.org/licenses/by/4.0/)

## Slide 7
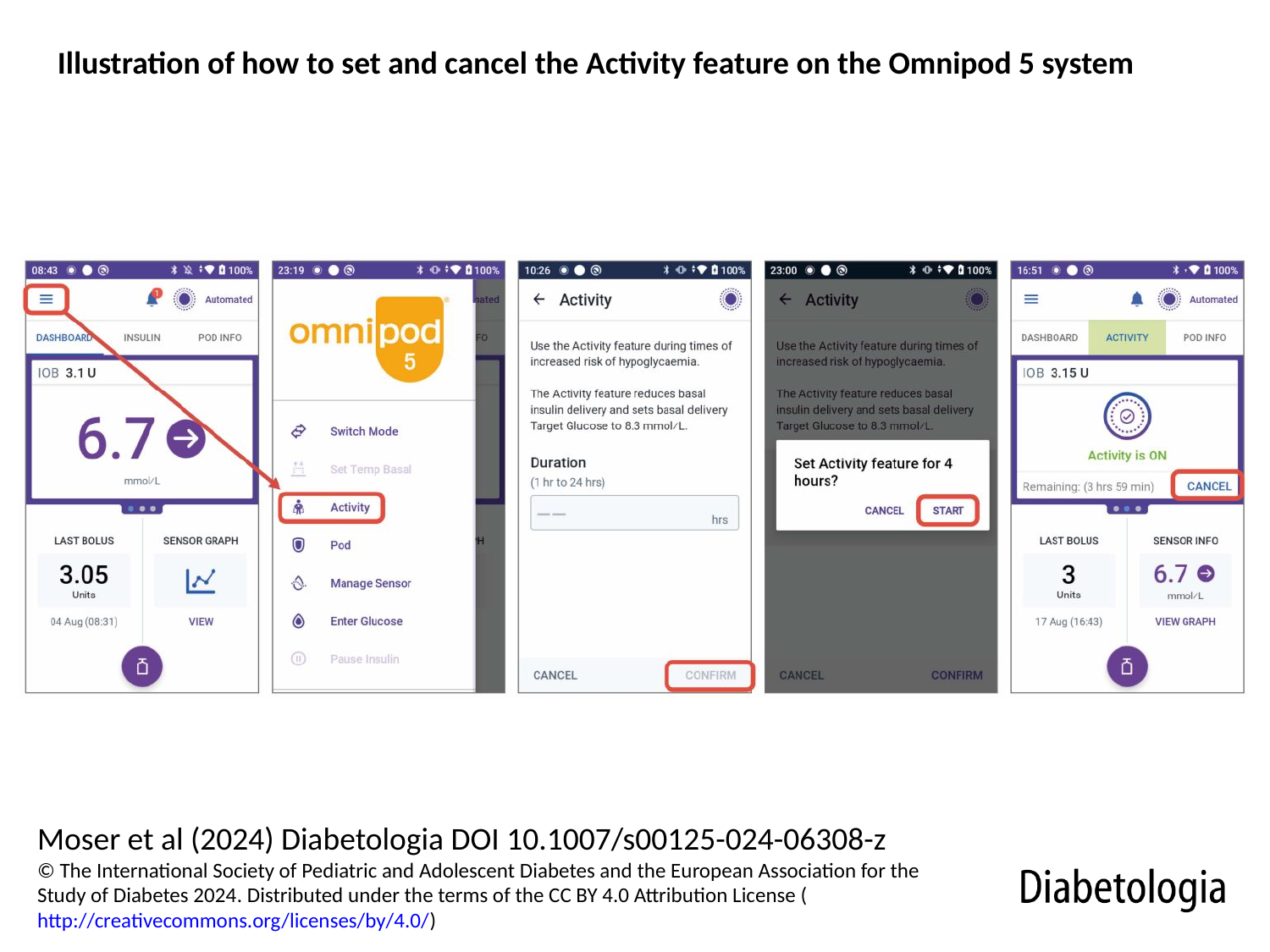

Illustration of how to set and cancel the Activity feature on the Omnipod 5 system
Moser et al (2024) Diabetologia DOI 10.1007/s00125-024-06308-z
© The International Society of Pediatric and Adolescent Diabetes and the European Association for the Study of Diabetes 2024. Distributed under the terms of the CC BY 4.0 Attribution License (http://creativecommons.org/licenses/by/4.0/)

## Slide 8
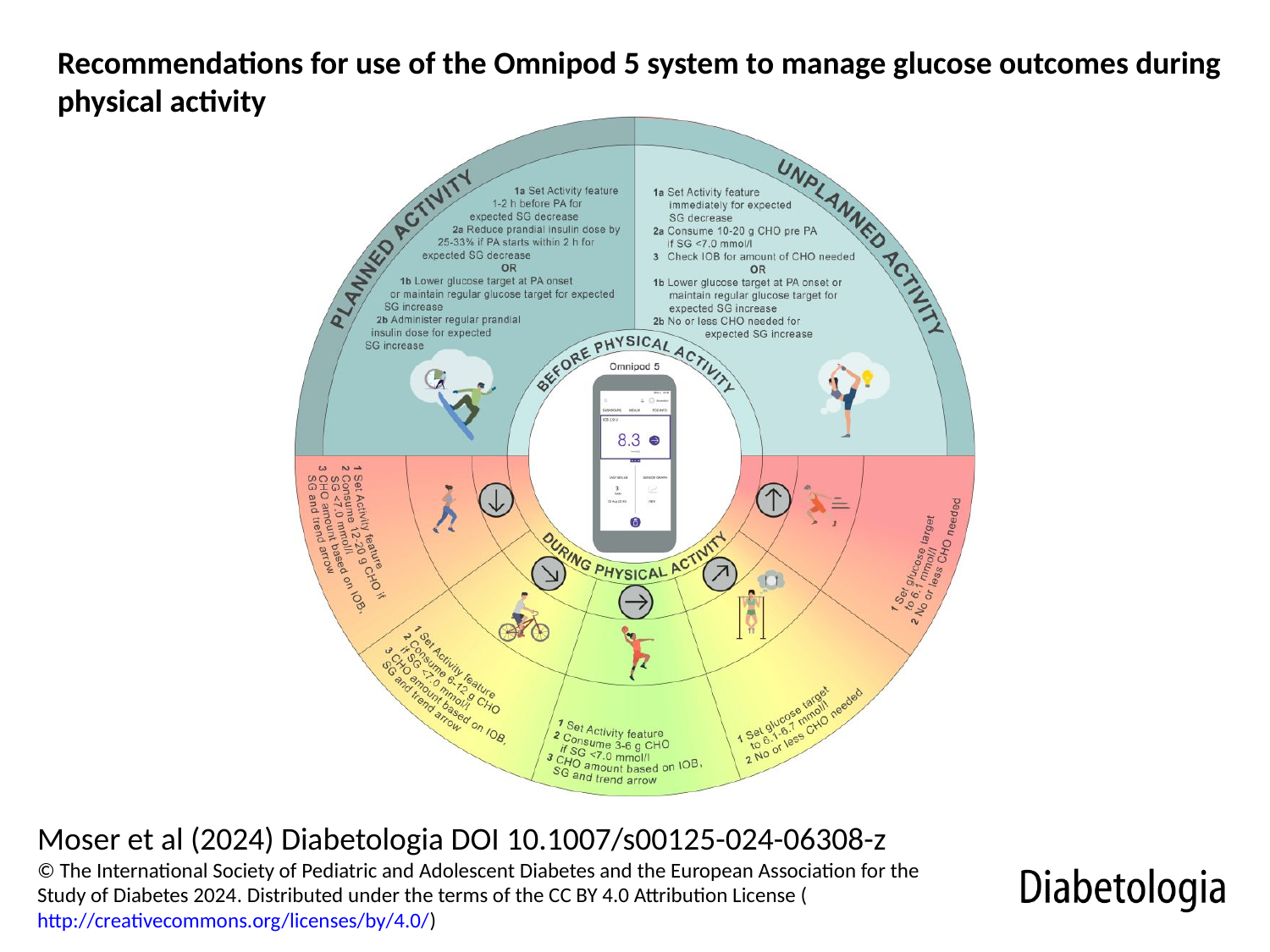

Recommendations for use of the Omnipod 5 system to manage glucose outcomes during physical activity
Moser et al (2024) Diabetologia DOI 10.1007/s00125-024-06308-z
© The International Society of Pediatric and Adolescent Diabetes and the European Association for the Study of Diabetes 2024. Distributed under the terms of the CC BY 4.0 Attribution License (http://creativecommons.org/licenses/by/4.0/)

## Slide 9
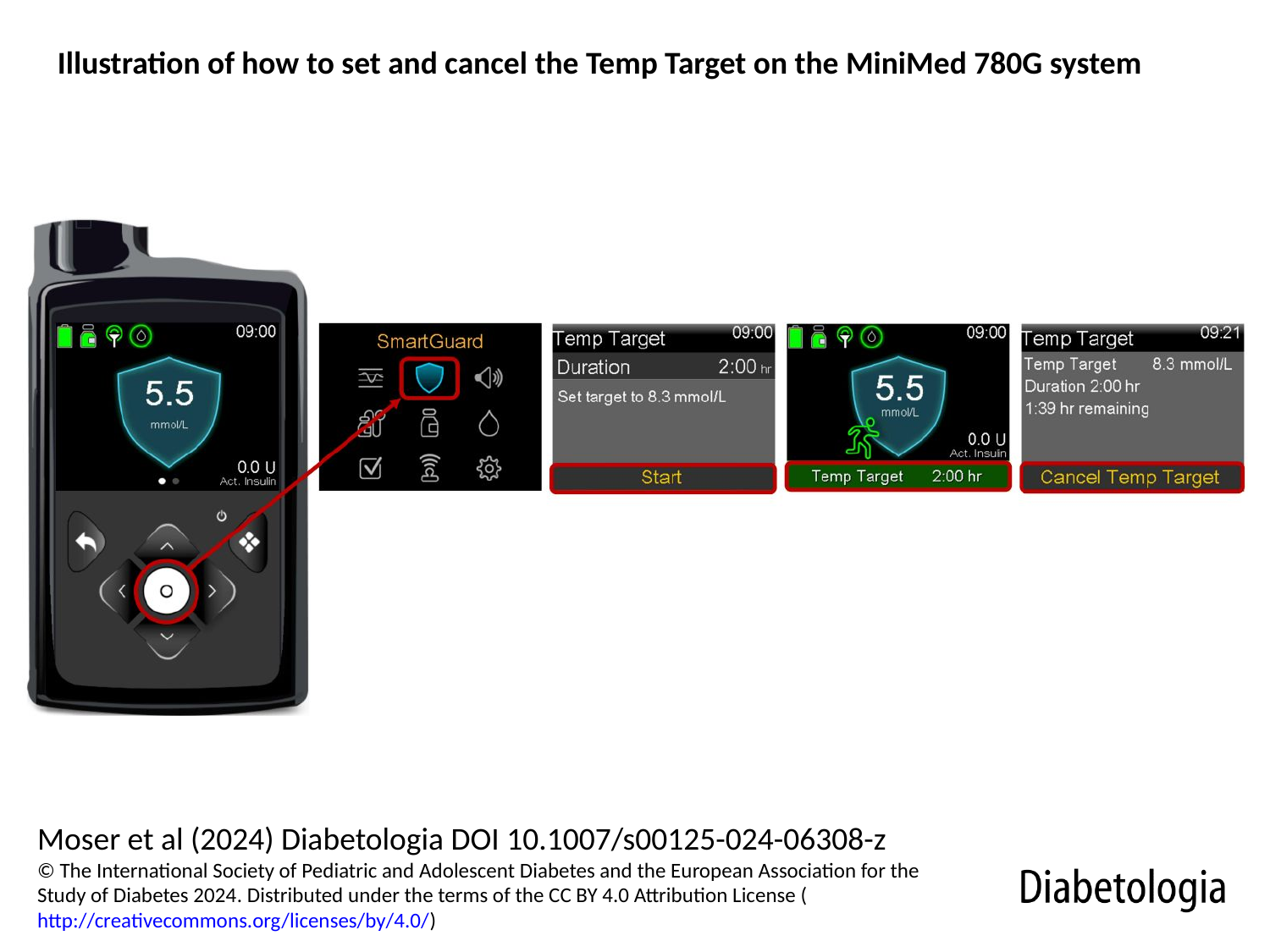

Illustration of how to set and cancel the Temp Target on the MiniMed 780G system
Moser et al (2024) Diabetologia DOI 10.1007/s00125-024-06308-z
© The International Society of Pediatric and Adolescent Diabetes and the European Association for the Study of Diabetes 2024. Distributed under the terms of the CC BY 4.0 Attribution License (http://creativecommons.org/licenses/by/4.0/)

## Slide 10
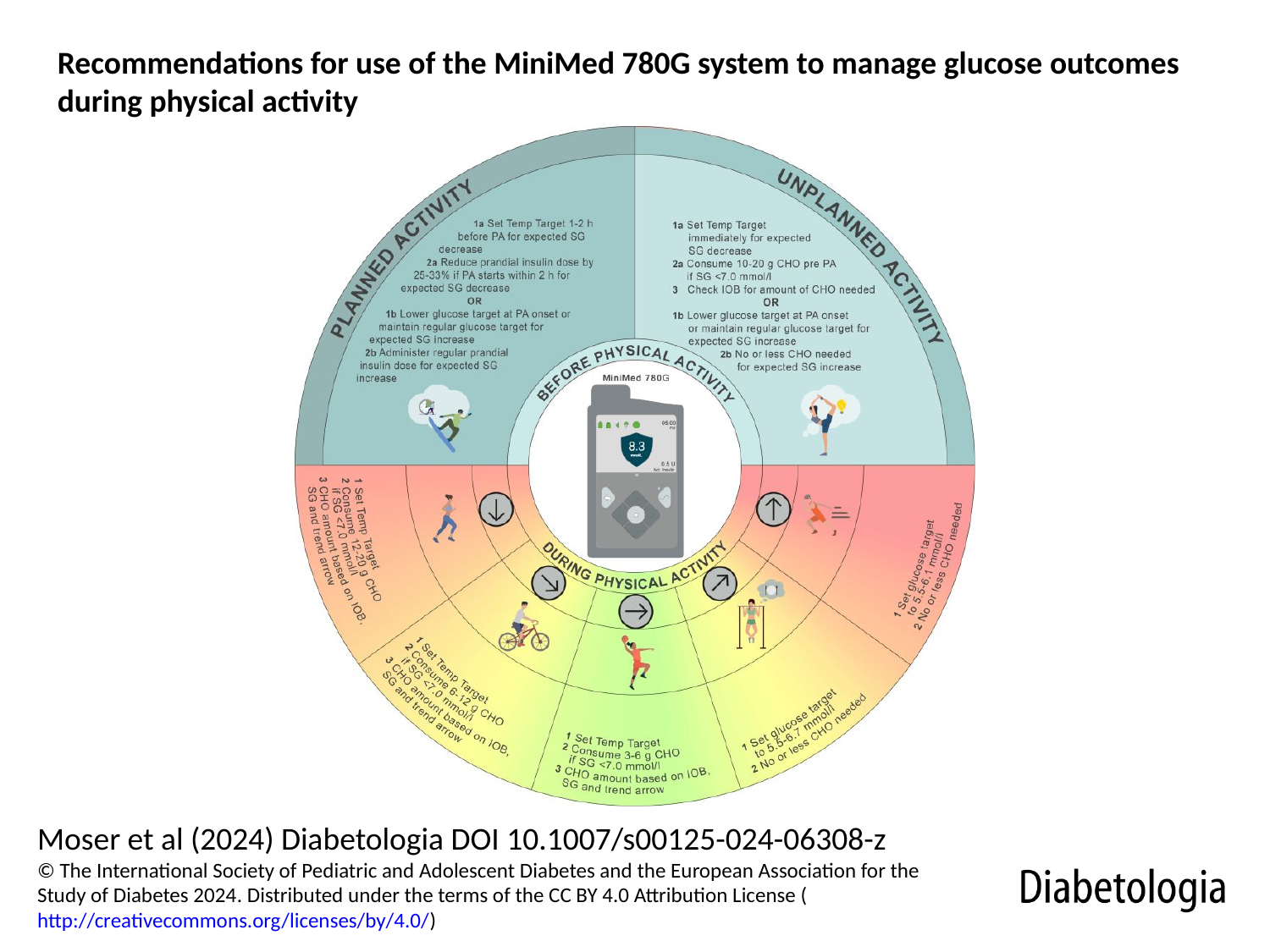

Recommendations for use of the MiniMed 780G system to manage glucose outcomes during physical activity
Moser et al (2024) Diabetologia DOI 10.1007/s00125-024-06308-z
© The International Society of Pediatric and Adolescent Diabetes and the European Association for the Study of Diabetes 2024. Distributed under the terms of the CC BY 4.0 Attribution License (http://creativecommons.org/licenses/by/4.0/)

## Slide 11
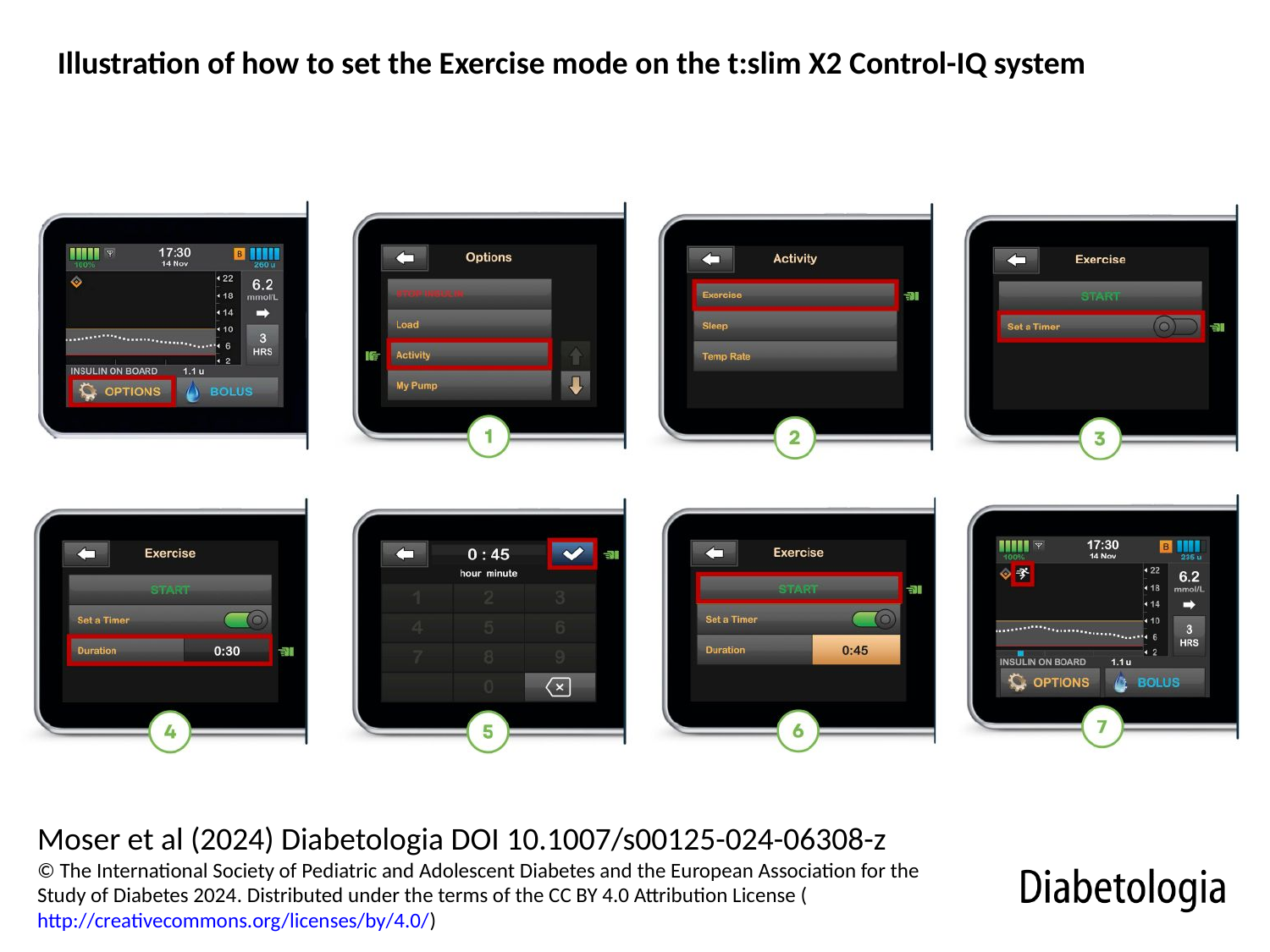

Illustration of how to set the Exercise mode on the t:slim X2 Control-IQ system
Moser et al (2024) Diabetologia DOI 10.1007/s00125-024-06308-z
© The International Society of Pediatric and Adolescent Diabetes and the European Association for the Study of Diabetes 2024. Distributed under the terms of the CC BY 4.0 Attribution License (http://creativecommons.org/licenses/by/4.0/)

## Slide 12
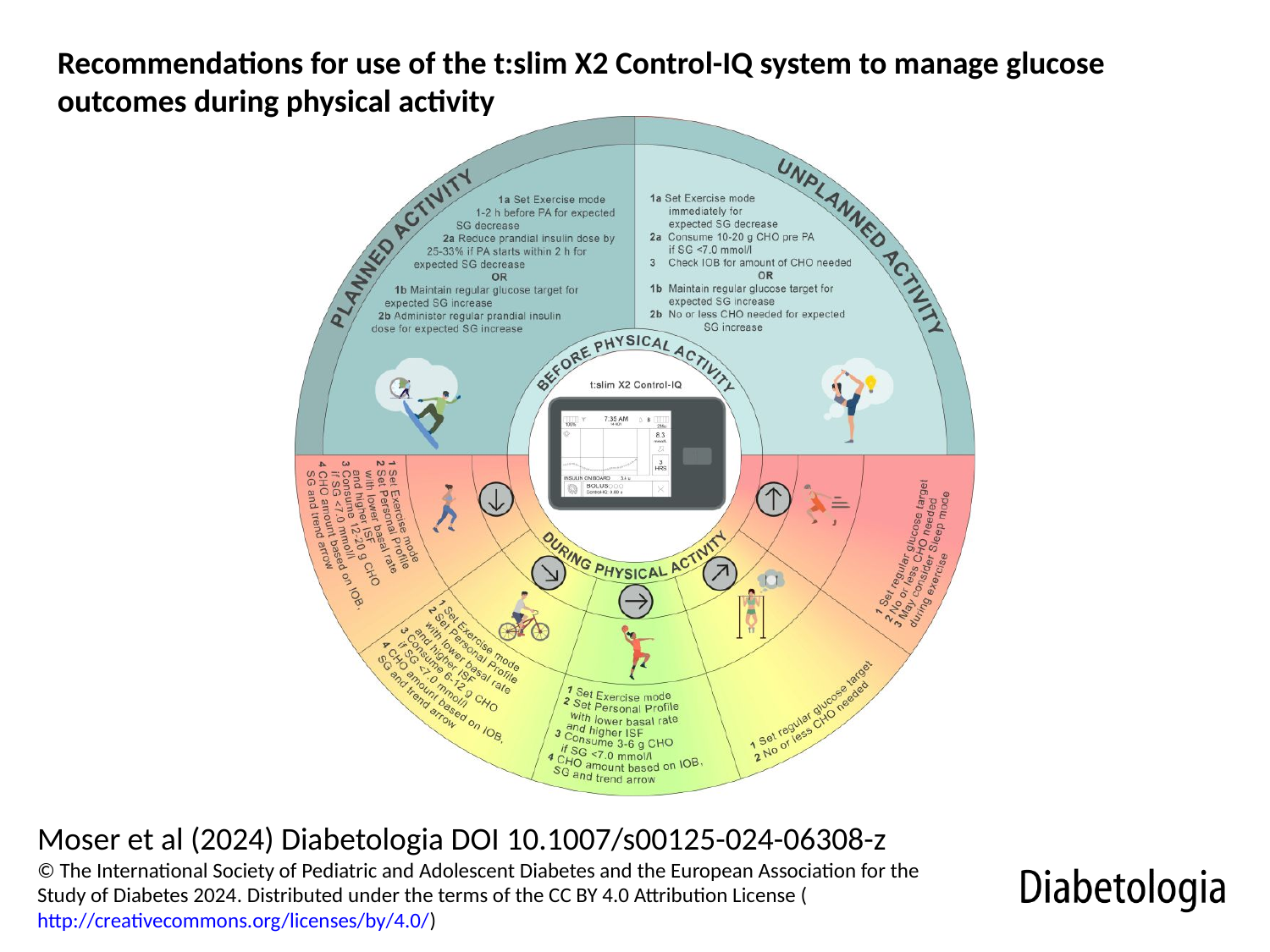

Recommendations for use of the t:slim X2 Control-IQ system to manage glucose outcomes during physical activity
Moser et al (2024) Diabetologia DOI 10.1007/s00125-024-06308-z
© The International Society of Pediatric and Adolescent Diabetes and the European Association for the Study of Diabetes 2024. Distributed under the terms of the CC BY 4.0 Attribution License (http://creativecommons.org/licenses/by/4.0/)
